# Rapamycin Plays a Pivotal Role in the Potent Antifungal Activity Exhibited Against *Verticillium dahliae* by *Streptomyces iranensis* OE54 and *Streptomyces lacaronensis* sp. nov. Isolated from Olive Roots

**DOI:** 10.3390/microorganisms13071622

**Published:** 2025-07-09

**Authors:** Carla Calvo-Peña, Marina Ruiz-Muñoz, Imen Nouioui, Sarah Kirstein, Meina Neumann-Schaal, José María Sánchez-López, Seyedehtannaz Ghoreshizadeh, Rebeca Cobos, Juan José R. Coque

**Affiliations:** 1 Instituto de Investigación de la Viña y el Vino, Escuela de Ingeniería Agraria, Universidad de León, 24009 León, Spain; ccalp@unileon.es (C.C.-P.); mruim@unileon.es (M.R.-M.); sghore00@estudiantes.unileon.es (S.G.); 2 German Collection of Microorganisms and Cell Cultures, 38124 Braunschweig, Germany; imen.nouioui@dsmz.de (I.N.); sarah.kirstein@dsmz.de (S.K.);; 3 Braunschweig Integrated Centre of Systems Biology (BRICS), Rebenring 56, 38106 Braunschweig, Germany; 4 Biomar Microbial Technologies, 24009 Armunia, Spain; jm.sanchez@biomarmt.com

**Keywords:** Verticillium wilt, olive, endophytic streptomycetes, biocontrol, antifungal compounds, rapamycin

## Abstract

Verticillium wilt, caused by *Verticillium dahliae*, poses a significant threat to olive trees (*Olea europaea* L.). The isolation of endophytic *Streptomyces* strains from olive roots has led to the discovery of several strains showing strong antifungal activity against *V. dahliae*, as demonstrated through in vitro and small-scale soil experiments. Molecular analyses confirmed that strain OE54 belongs to *Streptomyces iranensis*. The main antifungal compound identified in this strain was rapamycin. Rapamycin displayed potent antifungal effects, notably inhibiting conidiospore germination (IC_50_ = 87.36 μg/mL) and the hyphal growth of *V. dahliae*, with a minimum inhibitory concentration (MIC_50_) of 3.91 ng/mL. Additionally, a second rapamycin-producing strain, OE57^T^, was isolated. Phenotypic and genotypic analyses indicated that OE57^T^ represents a new species, which is proposed to be named *Streptomyces lacaronensis* sp. nov., with OE57^T^ designated as the type strain (=DSM 118741^T^; CECT 31164^T^). The discovery of two endophytic rapamycin-producing *Streptomyces* strains residing within olive roots is especially notable, given the rarity of rapamycin production among microorganisms. These findings highlight the potential of rapamycin-producing *Streptomyces* strains in developing biofertilizers to manage *V. dahliae* and reduce the impact of Verticillium wilt on olive trees and other crops.

## 1. Introduction

*Verticillium* is a genus of ascomycete fungi responsible for vascular wilt diseases, collectively termed Verticillium wilt, which affect a diverse range of plant hosts [1]. Verticillium wilt is widely regarded as one of the most devastating fungal diseases globally, posing a significant threat to numerous economically important crops, including alfalfa, almond, pistachio, peach [2,3], cotton, lettuce, ornamental plants, potato, strawberry [1], hops [4], and olive [3,5], among others. While different *Verticillium* species contribute to the disease, *Verticillium dahliae* is the most prevalent and virulent, largely due to its broad host range and extensive geographic distribution.

*Olea europaea* L. (olive) is one of the earliest domesticated and cultivated tree species, possessing substantial economic, historical, and social significance across many Mediterranean Basin countries. It serves primarily as the principal source of olive oil, a fundamental component of the Mediterranean diet [5]. However, in recent years, Verticillium wilt caused by *V. dahliae* has emerged as a severe threat to olive cultivation, with no effective control treatments currently available. This challenge is exacerbated by the pathogen’s ability to establish itself within plant tissues, rendering curative treatments ineffective. Given that *V. dahliae* is a soil-borne fungal pathogen that invades plants through their root systems, considerable attention has been directed toward developing biocontrol agents (BCAs) capable of colonizing the rhizosphere, or root tissues, as endophytic strains, thereby forming a protective barrier against infection [6]. Numerous studies have demonstrated the potential of various fungal BCAs in reducing the soil inoculum density and mitigating the disease severity, including *Fusarium oxysporum* [7,8], *Mucor* sp., *Rhizopus* sp., and *Phoma* sp. [9], as well as *Trichoderma asperellum* [10]. Additionally, entomopathogenic fungi such as *Beauveria bassiana* and *Metarhizium brunneum* have been investigated for their potential biocontrol efficacy [11]. Research has also explored the effectiveness of bacterial BCAs in managing *V. dahliae* infection, including various *Pseudomonas* strains [12,13] and members of the *Bacillales* order, such as *Paenibacillus alvei* K165 [14] and *Bacillus velezensis* [15].

Recent research underscores the potential efficacy of *Streptomyces* species as new and promising BCAs against Verticillium wilt in olive trees [16,17]. Members of the *Streptomyces* genus exhibit a range of distinctive properties that make them strong candidates for the development of BCAs targeting soil-borne fungal pathogens [6]. These characteristics include the following: (1) *Streptomyces* species are predominantly soil saprophytes and are ubiquitously distributed in diverse environments worldwide, including soils and sediments, where they are found in considerable abundance [18]. Some studies indicate that *Streptomyces* comprises approximately 0.06% of the bacterial abundance in soil, as determined from the 16S rRNA gene libraries [19]. (2) These species can effectively colonize the root environments of diverse plants, with a significant presence in both the rhizosphere [20] and the endosphere [21,22,23]. This colonization contributes to plant health and promotes growth, reinforcing their role in biological control strategies. (3) *Streptomyces* is the foremost genus in terms of bioactive secondary metabolite production, including numerous antibiotics and antifungal (AF) compounds [24]. Indeed, more than 50% of all clinically relevant antibiotics are derived from *Streptomyces* species [25]. (4) The antimicrobial properties of *Streptomyces* species extend beyond antibiotic production and are supported by additional mechanisms, such as the secretion of extracellular hydrolytic enzymes [26], the emission of volatile organic compounds [27], and the release of proteinaceous umbrella toxins [28]. (5) With over 755 validly named species (https://lpsn.dsmz.de/genus/streptomyces, accessed on 1 June 2025), *Streptomyces* constitutes one of the most taxonomically diverse bacterial genera. Ongoing discoveries of new species continue to expand their antimicrobial and AF capabilities, further reinforcing their importance in biological control applications.

This study describes the isolation and characterization of endophytic *Streptomyces* strains from the internal root tissues of olive plants, followed by their selection based on the potent AF activity against *Verticillium dahliae*. The two most effective isolates were identified as rapamycin producers, with one representing a new species within the *Streptomyces* genus. Ultimately, this research seeks to improve the health of vegetatively propagated olive plants in nursery settings by promoting root colonization by these beneficial microorganisms or developing biofertilizers to control this devastating disease.

## 2. Materials and Methods

### 2.1. Isolation of Culturable Endophytic Streptomycetes from Olive Roots

Root samples from three adult olive trees were collected in a plot belonging to the company Río Lacarón S.L. (La Garrovilla, Spain), at 215 m above sea level (38°55′08.1” N 6°31′27.7” W). Root samples were placed in sterile plastic bags and maintained at 4 °C until processing. Endophytic streptomycetes were isolated from the interior of root tissue samples. Roots were washed in sterile phosphate buffer (PBS) and sonicated (160 W; 2 min) to dislodge soil and organic matter from the sample surface. After drying at room temperature (RT), roots were cut into 2.0 cm-long fragments. Root fragments were surface-sterilized by immersing in 20 mL of Tween 20 (0.1%) for 30 s, followed by sodium hypochlorite (1%) for 6 min, and then Na_2_S_2_O_3_ (2.5%) for 10 min to remove the residual chlorine. Next, samples were washed three times with sterile water, submerged in 70% (*v*/*v*) ethanol for 6 min, followed by being subjected to three washes with sterile water and air-dried in a laminar flow hood (Telstar AV-100; Telstar, Terrassa, Spain). To confirm the effectiveness of the surface disinfection process, 0.2 mL of liquid from the final washing step was spread onto International *Streptomyces* Project 2 (ISP 2) [29] agar media and incubated at 28 °C. The isolation of endophytic streptomycetes was carried out from 5 g of previously disinfected root material. This material was cut into small fragments (using a scalpel under sterile conditions) that were later frozen in liquid nitrogen and ground into a fine powder using a mortar and pestle. Then, 20 mL of a solution containing yeast extract (6%, *w*/*v*) and sodium dodecyl sulfate (0.5%; *w*/*v*) were added to the samples and incubated at 40 °C for 15–30 min. Ten-fold serial dilutions of the samples were plated on starch-casein agar (SCA) [30]) and ISP 2 agar media, containing pimaricin (100 μg/mL) and nalidixic acid (50 μg/mL), to avoid fungi and Gram-negative bacterial growth, respectively. Plates were incubated at 28 °C for 3 to 7 days. Different isolates were selected based on their morphological and cultural characteristics, such as colony properties, presence/absence of aerial mycelia, spore mass color, distinctive reverse colony color, and production of diffusible pigments. The isolates were routinely cultivated and maintained the isolates on MEY (Maltose Yeast Extract) medium [31] at 4 °C. Spore-producing isolates were preserved as spore suspensions at −20 °C in glycerol (40%; *w*/*v*).

### 2.2. In Vitro Selection of Isolates Based on Antifungal Activity in Plate Assays

The AF activity of all isolated *Streptomyces* strains was checked using an in vitro plate assay [22]. Briefly, isolates were inoculated onto potato dextrose agar (PDA) plates (Sigma-Aldrich, Madrid, Spain) within a 1.0 cm^2^ area, with four isolates per plate, positioned 1 cm from the plate’s edge. A 0.5 cm agar plug containing the *V. dahliae* V937I strain [32] was placed at the center of each plate. The plates were incubated at 25 °C for up to 12 days, after which the growth inhibition zones were measured. To quantify the AF activity of the most effective isolates, they were further tested individually on MEY agar plates, where bacterial colonies were arranged in a circular pattern 1 cm from the plate’s edge. A *V. dahliae*-containing agar plug was placed at the center, and plates were incubated at 25 °C for up to 10 days. The inhibition index (I) was calculated using the formula: I index (%) = [(Rc − R)/Rc] × 100, where R represents the radius of the fungal colony in the presence of the bacterial isolate, and Rc is the maximum radius of the fungal colony in the control condition. Each assay was performed in triplicate for all tested strains. 

### 2.3. Analysis of Antifungal Activity in Small-Scale Soil Experiments

The AF activity in soil of selected isolates was evaluated using small-scale soil tests, following the methodology described by Calvo-Peña et al. (2023) [16]. Soil samples were collected from a depth of 20–30 cm after removing the uppermost 5 cm of surface soil. Five subsamples were taken, combined, and homogenized for analysis. Physicochemical properties of the soil were determined at the Laboratory of Instrumental Techniques (University of León, Spain). Parameters analyzed included soil texture and mechanical composition, organic carbon content, pH, electrical conductivity, nitrogen levels, available phosphorus, exchangeable potassium, micronutrients (Fe, Cu, Mn, and Zn), magnesium, available boron, and total and active carbonates. These analyses were conducted following standard procedures as described by Calvo-Peña et al. (2023) [16].

### 2.4. In Vitro Confrontation of Endophytic Streptomyces Strains

To check potential antagonistic interactions between endophytic *Streptomyces* strains, pairwise confrontation assays were conducted following the methodology initially described by Schrey et al. (2012) [33], with minor modifications as detailed by Calvo-Peña et al. (2023) [16].

### 2.5. Molecular Identification of Strains and Genome Sequencing

Strains exhibiting the highest AF activity were identified at the genus level through partial sequencing of the 16S rRNA gene. Genomic DNA was extracted following the protocol described by Hopwood et al. (1985) [34]. The 16S rRNA genes were amplified using the oligonucleotide primers 27F and 1492R [35], and the resulting sequences were compared to those of type strains available in the EzTaxon-e database [36]. Sequence alignments were performed using MEGA v11.0 (http://www.megasoftware.net/), and evolutionary distances were calculated using the Kimura two-parameter (K2P) model for nucleotide sequences [37].

For putative species-level identification, a multilocus sequence analysis (MLSA) was conducted using five housekeeping genes: *atpD* (ATP synthase F1, β-subunit), *gyrB* (DNA gyrase B subunit), *recA* (recombinase A), *rpoB* (RNA polymerase, β-subunit), and *trpB* (tryptophan synthase, β-subunit) [38]. Amplification of these genes was performed using primers and conditions described by Guo et al. (2008) [39] and Rong et al. (2009) [40]. The GenBank accession numbers for the housekeeping gene sequences are listed in Table 1.

A phylogenetic tree was constructed based on the concatenation of the five housekeeping genes. Sequences were manually trimmed to uniform positions before alignment using MEGA v11.0, incorporating sequences from type strains obtained from the ARS Microbial Genomic Sequence Database (https://blogs.cornell.edu/buckley/streptomyces-sequence-database/, accessed on 20 January 2025). Phylogenetic analysis was performed using the maximum likelihood method with the Kimura two-parameter model (Kimura, 1980 [37]). MLSA evolutionary distances were calculated using MEGA v11.0 based on K2P distance. Strains exhibiting an MLSA evolutionary distance of ≤0.007 were considered conspecific, following the empirical guidelines established by Rong and Huang (2012) [38].

The genomes of strains OE54 and OE57^T^ were sequenced using next-generation sequencing (NGS) technologies, employing both the PacBio Sequel II system and the Illumina platform, as provided by Macrogen Inc. (Seoul, Republic of Korea). De novo genome assembly was performed using Canu v2.2 [41], and assembly quality was assessed with QUAST v5.3.0 [42]. Draft genome annotation was conducted using the RAST-SEED webserver (https://rast.nmpdr.org/) [43]. The genome sequences of strains OE54 and OE57^T^ have been deposited in the GenBank database under the accession numbers JBLHDJ000000000 and JBLHDK000000000, respectively. The associated BioProject accession number is PRJNA1185486.

### 2.6. Cultural and Growth Properties

The cultural characteristics of the strains were assessed on various agar media, including ISP 1 (DSMZ 1764), ISP 2 (DSMZ 987), ISP 3 (DSMZ 609), ISP 4 (DSMZ 547), ISP 5 (DSMZ 993), ISP 6 (DSMZ 1269), ISP 7 (DSMZ 1619) [29], nutrient agar (DSMZ 1) (Scharlab S.L., Sentmenat, Spain), Bennett’s agar (DSMZ 548) (HiMedia Laboratories GmbH, Modautal, Germany), and trypticase soy agar (TSA) (DSMZ 535) (Scharlab S.L.). Plates were incubated at 28 °C for seven days. Growth tolerance was evaluated across a range of temperatures (4 °C to 45 °C) and pH values (5.0 to 12.0) using DSMZ 65 medium (glucose 4 g/L; yest extract, 4 g/L; malt extract, 10 g/L; CaCO_3_, 2 g/L; agar, 20 g/L; pH 7.2). All tests were conducted in duplicate with bacterial suspensions standardized to 5 on the McFarland scale. The coloration of aerial and substrate mycelium, as well as the production of diffusible pigments, was recorded and compared against standard color charts. The close phylogenomic neighbors, *Streptomyces rapamycinicus* DSM 41530^T^ and *Streptomyces iranensis* DSM 41954^T^, were included in this study and obtained from the Leibniz Institute DSMZ—German Collection of Microorganisms and Cell Cultures.

### 2.7. Phenotypic and Chemotaxonomic Properties

Freeze-dried biomass from a 7-day old culture of strain OE57^T^ and its phylogenomic neighbor, *Streptomyces rapamycinicus* DSM 41530^T^, prepared in ISP 2 medium with shaking at 120 rpm, was used for chemotaxonomic analysis. Standard chromatographic procedures were applied to determine isomeric forms of diaminopimelic acid (A2pm) [44], whole cell sugars [45], and the polar lipid profile of strain OE57^T^ [46,47]. Cellular fatty acids and isoprenoid quinones of the strain were extracted from wet biomass prepared under the same growth conditions as mentioned above. Cellular fatty acids were analyzed after conversion into fatty acid methyl esters by gas chromatography (GC) using an Agilent 6890N equipment (Agilent Technologies, Santa Clara, CA, USA) as described by Sasser [48]. The fatty acid pattern was confirmed via gas chromatography–mass spectrometry (GC-MS) using an Agilent GC–MS 7000D instrument, as described in Vieira et al. (2021) [49]. The position of double bonds was determined by a derivatization with dimethyl sulfide and a further GC-MS measurement [50]. Menaquinone extracts were analyzed by high-performance liquid chromatography (HPLC), and identified with both a diode-array detector (DAD) and high-resolution MS [51]. The biochemical and enzymatic properties of the OE54 and OE57^T^ strains and their phylogenomic relatives (*S. iranensis* DSM 41954^T^ and *S. rapamycinicus* DSM 41530^T^) were characterized using API-ZYM and API 20NE test strips (bioMérieux, Lyon, France). For API 20NE, inoculation of the cupules was performed at 28 °C with a bacterial suspension standardized to a turbidity of 5 on the McFarland scale, and results were recorded at 24 and 48 h. API-ZYM assays were conducted at 28 °C instead of the standard 37 °C, with an incubation period of 24 h.

### 2.8. Phylogeny and Comparative Genomic Analyses

For preliminary taxonomic identification at the genus level, partial 16S rRNA gene sequences were analyzed as described above. Complete 16S rRNA gene sequences (average length 1485 bp), obtained from whole-genome sequencing, were used for more refined phylogenetic analyses. Pairwise 16S rRNA gene sequence similarities between the strains and their closest phylogenetic relatives were estimated using the parameters described by Meier-Kolthoff et al. (2013) [52], as implemented in the Genome-to-Genome Distance Calculator (GGDC) 2.1 (http://ggdc.dsmz.de) [53]. Reference strain sequences were retrieved from the EzBioCloud database (https://www.ezbiocloud.net/) [54]. Phylogenetic relationships were inferred using maximum-likelihood (ML) trees based on both 16S rRNA gene and whole-genome sequences. These analyses were conducted using the Type (Strain) Genome Server (TYGS) v1.0 (https://tygs.dsmz.de/), a bioinformatics tool for genome-based taxonomic classification [55]. The TYGS platform utilizes an extensive, continuously updated genome database containing taxonomic and nomenclatural information for all available type strains. This system enables full-genome phylogenetic comparisons by aligning the genome sequence of the strain of interest with those of type strains, facilitating taxonomic placement. Genomic relatedness between the strains and their closest phylogenomic relatives was assessed using digital DNA–DNA hybridization (dDDH) values calculated with GGDC 2.1 [56] and differences in genomic G + C content.

### 2.9. In Silico Screening for Secondary Metabolites

The presence of putative biosynthetic gene clusters (BGCs) in the genome sequences of the strains and their closest phylogenomic relatives was analyzed using the antiSMASH web tool v7.0 [57]. antiSMASH predicts secondary metabolite BGCs by identifying relevant genes and grouping them into clusters based on sequence similarity and genomic proximity. The tool employs domain recognition through HMMER to detect key enzyme domains, and the identified clusters are compared against multiple databases to predict the potential metabolites they may encode [58]. The analysis was performed using the relaxed strictness setting with all additional features enabled, including ClusterBLAST (https://docs.antismash.secondarymetabolites.org/modules/clusterblast/, accessed on 15 December 2024)and MIBiG cluster comparisons (https://mibig.secondarymetabolites.org/, accesed on 15 December 2024). For clusters of particular interest, manual curation and further assessments were conducted using BLAST (v2.16.0. https://blast.ncbi.nlm.nih.gov/Blast.cgi, accessed on 15 December 2024). Genes of unknown function were analyzed with InterProScan (version 5.72-103.0; https://ebi.ac.uk/interpro/about/interproscan; accessed on 15 December 2024) to predict their potential roles within the clusters. Additionally, CAGECAT (https://cagecat.bioinformatics.nl; accessed on 15 December 2024) [59] and BiG-SCAPE v1.1.9 (https://git.wageningenur.nl/medema-group/BiG-SCAPE; accessed on 15 December 2024) [60] were used to assess similarities among the identified clusters.

### 2.10. Fermentation, Culture Extract Preparation, and HPLC Analysis of Antifungal Activity

To analyze the production of AF compounds in liquid media, crude extracts of strains OE54 and OE57^T^ grown in ISP 2 medium were prepared following the method described by Das et al. (2018) [61]. Briefly, the cell-free culture supernatant was vigorously mixed with ethyl acetate in a 1:1 (*v*/*v*) ratio for 30 min, followed by separation of the organic layer. The ethyl acetate extract was evaporated under vacuum using a CentriVap concentrator (Labconco, Kansas City, MO, USA), and the residue was dissolved in 80% methanol to a final concentration of 1 mg/mL. To test AF activity, 60 μL of the crude extract were used following the AF bioassay described above. The remaining extract was filtered through Corning^®^ Costar^®^ Spin-X^®^ centrifuge tube filters (0.45 μm pore size; Merck KGaA, Darmstadt, Germany) and stored at −20 °C until further analysis. HPLC analysis of the crude extracts was performed using an Agilent 1200 Series Gradient HPLC System (Agilent Technologies), following the chromatographic method described by Awla et al. (2020) [62]. The system was equipped with a quaternary pump delivery system (G1311A), a preparative autosampler (G1329A), a diode array multi-wavelength detector (G7115A), and an analytical fraction collector (G1364F) with an Autosampler Thermostat (G1330B). A 10 μL sample was injected and resolved using an analytical Lichospher RP18 column (40 × 250 mm; 5 μm) (Teknokroma, San Cugat del Vallés, Spain).

### 2.11. Purification of Rapamycin and Epoxinnamide by Vacuum Flash Chromatography (VFC)

Strain OE54 was cultivated in 500 mL indented Erlenmeyer flasks containing 125 mL of ISP 2 medium. Each flask was inoculated with 10 agar plugs (0.5 cm diameter) of the bacterial strain, which had been previously grown on SCA plates until sporulation was achieved. Cultures were incubated at 30 °C with agitation at 150 rpm for 72 h. The resulting fermentation broth (6.5 L) was mixed with the adsorption resin Amberlite XAD-1180 (Dupont, Mississauga, ON, Canada) and filtered using Radifil RW50 as a filtration aid (Agrovin, Alcázar de San Juan, Spain). The exhausted broth was discarded, and the resin–mycelium mixture was extracted with 3 L of EtOAc/MeOH (3:1). Following filtration and solvent evaporation under vacuum, 2.90 g of crude extract was obtained. The crude extract was analyzed by LC-MS and tested for AF activity using a plate bioassay. A 2 mg aliquot of the extract was dissolved in 400 μL of MeOH and analyzed using a 1290 Infinity II HPLC system (Agilent) coupled to an Agilent 6230 time-of-flight LC/MS (LC/TOF) mass spectrometer. Chromatographic separation was performed on an Agilent Zorbax Eclipse Plus C18 RRHD column (2.1 × 50 mm, 1.8 μm particle size) with a gradient system of MeOH/H_2_O (0.1% formic acid), increasing from 20% to 100% MeOH over 8 min. UV detection was performed at 220 nm with a flow rate of 0.6 mL/min. Upon confirming that the crude extract exhibited AF activity, it was subjected to VFC using silica gel (40–60 μm, 60 Å) (Thermo Fisher Scientific Inc., Waltham, MA, USA) as the stationary phase and eluted with a stepwise gradient of hexane/EtOAc/MeOH. The obtained fractions were analyzed by LC-MS, as described above, and tested for AF activity. Those active fractions were combined and further purified by C18 reversed-phase column chromatography using MeOH/H_2_O (9:1). Some active fractions were pooled and subjected to an additional round of VFC using a stepwise gradient of dichloromethane/MeOH. This process yielded 12 fractions (F1′–F12′), each of which was tested again for AF activity.

### 2.12. Structural Elucidation of Rapamycin and Epoxinnamide

The purified compound was analyzed by nuclear magnetic resonance (NMR) for structural elucidation. The molecular formula of the major compound detected in the active fractions was determined using high-resolution electrospray ionization mass spectrometry (HRESIMS) and Nuclear Magnetic Resonance (NMR) spectroscopy. Structural elucidation was conducted through extensive NMR analyses, including ^1^H NMR, ^13^C NMR, ^1^H-^1^H COSY, gHSQC, and gHMBC experiments. ^1^H and ^13^C NMR spectra were recorded on a Varian Mercury 400 spectrometer (Agilent Technologies) operating at 400 MHz and 100 MHz, respectively. gHMQC and gHMBC experiments were performed using an inverse resonance probe. Chemical shifts are reported in parts per million (ppm) relative to the solvent signals (CDCl_3_: δH 7.26, δC 77.0). Mass spectrometric data were acquired using an Agilent 6230 time-of-flight LC/MS (LC/TOF) mass spectrometer, providing high-resolution mass measurements for molecular formula determination.

### 2.13. Analysis of Rapamycin’s Inhibitory Effect on Conidiospore Germination and Hyphal Growth

The effect of rapamycin on conidiospore germination was tested using a protocol based on López-Moral et al. (2022) [63]. Conidial suspensions were prepared from 12-day-old *Verticillium dahliae* V937I colonies grown on PDA and adjusted to a final concentration of 1 × 10^6^ conidia/mL. A 5 mg/mL stock solution of rapamycin (Thermo Fisher Scientific Inc.) was prepared in DMSO and diluted in 50% ethanol to generate working solutions (50 to 350 μg/mL). This approach minimized any potential inhibitory effects of DMSO and ethanol on conidiospore germination. For the germination assay, a 5 μL drop of the conidial suspension was placed in the center of a microscope coverslip and mixed with 5 μL of rapamycin solution. A negative control (0 μg/mL rapamycin) was prepared by mixing 5 μL of conidial suspension with 5 μL of 50% ethanol. Coverslips were placed inside Petri dishes containing water agar to create humid chambers and incubated at 25 °C in the dark for 10 h. Following incubation, conidial germination was halted by adding a 5 μL drop of 0.01% acid fuchsin in lactoglycerol (lactic acid:glycerol:water, 1:2:1) to each coverslip. The coverslips were then mounted onto slides for microscopic examination. The experiment was conducted in triplicate for each concentration and repeated twice. For each coverslip, 100 randomly selected conidia were examined under ×400 phase-contrast microscopy Olympus CX41 (Olympus Europe, Hamburg, Germany), and germinated conidia were counted. Conidia were considered germinated if the germ tube length was at least equal to the longitudinal axis of the conidia. The relative germination inhibition (RGI, %) was calculated as RGI = [(G_control_ − G_rap_)/G_control_] × 100, where G_control_ represents the percentage of germinated conidia in the control group (35% ethanol), and G_rap_ represents the percentage of germinated conidia in the presence of rapamycin. The data were used to generate a regression line, and the 50% inhibitory concentration (IC_50_) was determined via Probit analysis of mortality data. The effect of rapamycin on hyphal growth and the calculation of minimum inhibitory concentration (MIC) was determined using different rapamycin concentrations on PDA plates [64], with rapamycin concentrations ranging from 0 to 20 ng/mL.

## 3. Results

### 3.1. Isolation and Selection of Culturable Endophytic Streptomycetes Associated with Olive Roots and Their Antifungal Activity Against V. dahliae

**Figure 1:**
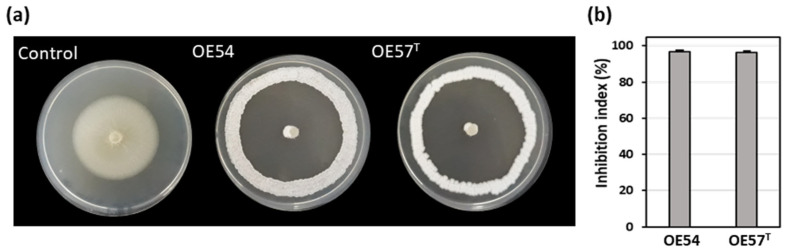
(**a**) Antifungal activity of the endophytic isolates OE54 and OE57^T^ against the phytopathogenic fungus *Verticillium dahliae* V937I, as determined by an in vitro bioassay-based screening; and (**b**) quantification of the antifungal activity by calculating the inhibition index. Data shown represent the mean values from three independent experiments, each conducted in duplicate.

A total of 106 putative different endophytic *Streptomyces* strains (designated OE1 to OE106, where OE denotes Olive Endophyte) were isolated from the internal tissues of olive roots. Selection was based on distinct morphological and cultural characteristics. In an in vitro bioassay-based screening, 27.3% (29 out of 106) of the isolates exhibited AF activity against *V. dahliae* strain V937I. Among them, two strains demonstrated particularly strong inhibitory effects on *V. dahliae* mycelial growth: strain OE54, with an inhibition index of 96.86% (±0.68), and strain OE57^T^, with an inhibition index of 96.21% (±0.91) (Figure 1).

### 3.2. Evaluation of Antifungal Activity in a Small-Scale Soil Assay

**Figure 2:**
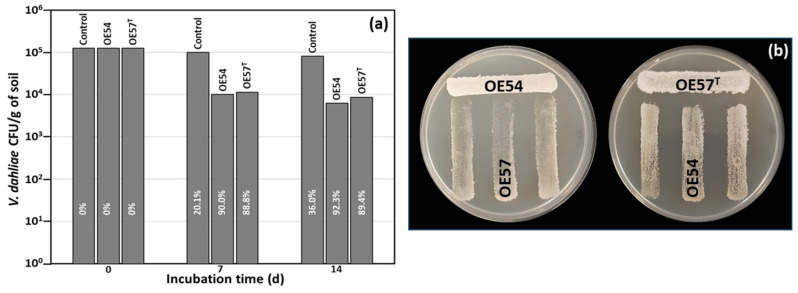
(**a**) Small-scale in vitro soil assay to test the antifungal activity of isolates OE54 and OE57^T^ against *V. dahliae*. The values shown are the average of two independent experiments made by duplicate. The inhibition percentages corresponding to each bar are shown in white letters. (**b**) Analysis of the cross interaction between both strains. Each strain was challenged with the other on a Petri dish co-culture bioassay 3 times (*n* = 9).

While many microbial isolates exhibit strong AF activity in in vitro assays, their efficacy under real soil conditions remains uncertain. This trait is crucial for their potential application as BCAs in field trials and for promoting root colonization in the case of endophytic strains. To address this, a small-scale in vitro soil experiment was designed to evaluate the AF activity of *Streptomyces* strains OE54 and OE57^T^, both individually and in combination, against *V. dahliae* under soil conditions. For this experiment, both strains were inoculated into sterile soil to eliminate potential interference from other microorganisms present in natural soil. The soil used was classified as loamy according to the USDA standard, with a pH of 7.98, an organic matter content of 6.59%, and a total nitrogen content of 0.32%. *V. dahliae* was introduced at an initial concentration of 1.25 × 10^5^ colony-forming units (CFUs) per gram of soil. Over time, its viability decreased by 20.1% and 36.0% at 7 and 14 days post-inoculation, respectively. Co-inoculation with strain OE54 led to a significant reduction in *V. dahliae* survival, with declines of 90.0% and 92.3% at 7 and 14 days, respectively. By the end of the experiment, only 6.20 × 10^3^ CFU/g of *V. dahliae* remained detectable (Figure 2a). Similarly, co-inoculation with strain resulted in *V. dahliae* reductions of 88.8% and 89.4% at 7 and 14 days, respectively, with a final viable count of 8.47 × 10^3^ CFU/g of soil (Figure 2a).

### 3.3. Strain-Specific Inhibition Patterns in Streptomyces–Streptomyces Interaction Bioassays

Considering the strong performance of both strains in the small-scale soil assay, we aimed to assess the potential negative interactions between OE54 and OE57^T^ that could hinder their combined application in future field trials. To this end, a co-culture bioassay was conducted (Figure 2b). The results indicated that strain OE54 exerted a minimal inhibitory effect on strain OE57 ^T^, reducing its growth by only 2.0% (±0.3). In contrast, strain OE57^T^ exhibited a slightly higher inhibitory effect on OE54, with a reduction of 9.58% (±2.0) (Figure 2b).

### 3.4. Molecular Identification of Isolates OE54 and OE57^T^ Using 16S rRNA Analysis, Multilocus Sequence Analysis (MLSA), and Comparative Genomics

**Figure 3:**
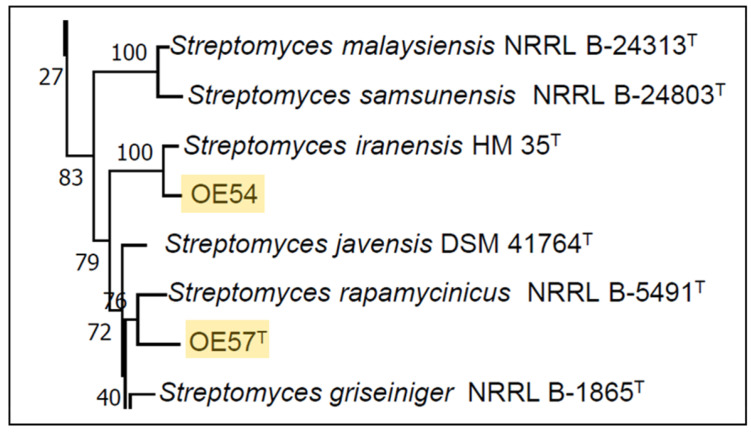
Partial view of the Neighbor-Joining tree derived from the MLSA analysis, illustrating the phylogenetic placement of *Streptomyces* strains OE54 and OE57^T^ (highlighted with yellow background). The complete tree is available in Supplementary Information (Figure S1).

A partial sequencing of the 16S rRNA gene from strain OE54 revealed a sequence similarity exceeding 99.00% with multiple *Streptomyces* species, including *S. iranensis* (99.93%), *S. rapamycinicus* (99.21%), *S. yogyakartensis* (99.07%), *S. javensis* (99.07%), *S. violaceusniger* (99.07%), and *S. demainii* (99.00%). Similarly, strain OE57^T^ exhibited a >99.00% sequence similarity with *S. yogyakartensis* (99.22%), *S. javensis* (99.22%), *S. violaceusniger* (99.22%), and *S. albiflaviniger* (99.14%). These findings confirmed that both isolates belong to the *Streptomyces* genus. However, accurate species-level identification was not possible, as all similarity values exceed the 98.65% threshold for prokaryotic species delineation based on 16S rRNA gene sequences [65]. Given the limitations of 16S rRNA for discriminating closely related *Streptomyces* species, an MLSA was conducted using five housekeeping genes (*atpD*, *gyrB*, *recA*, *rpoB*, and *trpB*). This analysis placed strain OE54 within a subclade with *S. iranensis* (Figure 3), with a Kimura 2-Parameter MLSA distance of 0.005 (Table 2), supporting its classification as *S. iranensis*, since strains with MLSA distances ≤ 0.007 are considered conspecific [38]. Conversely, strain OE57^T^ clustered within a subclade that included *S. rapamycinicus* (Figure 3), but showed a genetic distance of 0.011 (Table 2), suggesting it represents a distinct species.

The discovery of two phylogenetically related *Streptomyces* strains residing as endophytes in olive roots prompted further investigation through whole-genome sequencing. Strain OE57^T^ was sequenced at 94× coverage, yielding a genome size of 12.39 Mbp with a G + C content of 70.8%, 10,904 coding sequences, and 82 RNA genes, while strain OE54 was sequenced at 52× coverage, revealing a genome size of 11.71 Mbp with a G + C content of 71.0%, 10,077 coding sequences, and 82 RNA genes. The genomic features of both strains were consistent with those characteristics of the *Streptomyces* genus [66].

**Figure 4:**
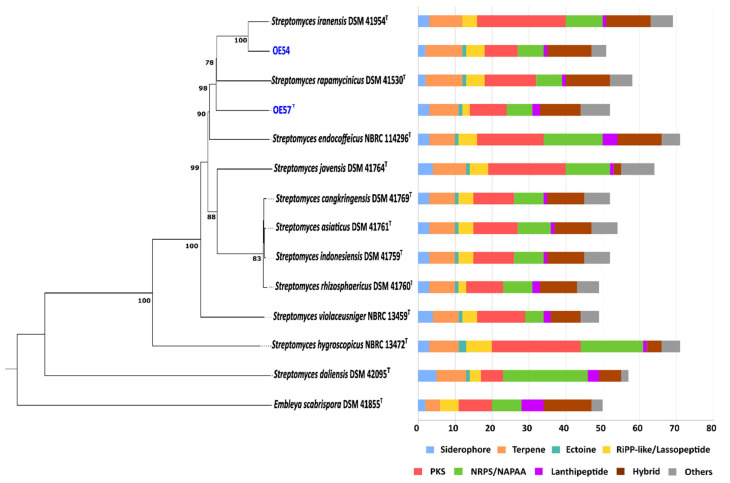
Whole-genome sequence-based phylogenetic tree of strains OE54 and OE57^T^ (highlighted in blue color) and their closest related species, constructed using the TYGS web server. The tree was inferred using FastME based on genome-to-genome distances computed with the Genome Blast Distance Phylogeny (GBDP) method, employing the d4 distance formula. Branch lengths are scaled according to GBDP distance values. The biosynthetic gene cluster (BGC) composition of each strain is displayed on the right side of the tree, with BGC classes color-coded according to the legend below.

**Figure 5:**
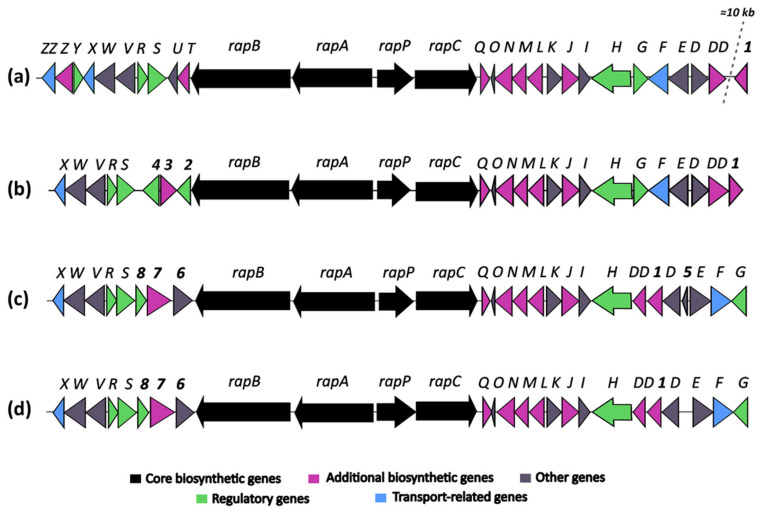
Comparative analysis of rapamycin biosynthetic gene clusters (BGCs) from the following: (**a**) *Streptomyces rapamycinicus* NRRL 5491^T^; (**b**) *Streptomyces* sp. OE57^T^; (**c**) *Streptomyces iranensis* DSM 41954^T^; and (**d**) *Streptomyces iranensis* OE54.

A whole-genome-based phylogenetic classification of strains OE54 and OE57^T^, along with their closest relatives, revealed a distinct grouping within a subclade that also includes *S. iranensis* and *S. rapamycinicus* (Figure 4). Consistent with previous MLSA and 16S rRNA phylogenetic analyses, this whole-genome phylogeny confirmed that strain OE54 belongs to the *S. iranensis* species (Figure 5). In contrast, while strain OE57 clustered within the same subclade, it represents a distinct and novel *Streptomyces* species.

Digital DNA–DNA hybridization (dDDH) values further corroborated these results. Strain OE54 showed a 81.7% similarity with *S. iranensis* DSM 41954^T^, exceeding the 70% threshold for species delineation [67], thereby confirming its classification as *S. iranensis*. On the other hand, OE57^T^ showed dDDH values of 60.3% with *S. iranensis* DSM 41954^T^, 60.0% with *S. rapamycinicus* DSM 41530^T^, and 56.3% with *S. endocoffeicus* NBRC 114296^T^. The dDDH similarity between OE54 and OE57^T^ was 59.8%. These dDDH values were well below the 70% threshold established for species delineation, supporting the recognition of strain OE57^T^ as a distinct *Streptomyces* species.

In addition, the full-length 16S rRNA gene sequences retrieved from the genome assemblies were used to construct a phylogenetic tree (Supplementary Figure S2), which corroborated the previous results. OE54 clustered with *S. iranensis*, while OE57^T^ grouped with species of the *S. violaceusniger* clade, particularly *S. javensis* and *S. violaceusniger*. Although these associations reflect close evolutionary relationships, they also underscore the limited resolution of the 16S rRNA gene for species-level discrimination within the *Streptomyces* genus.

### 3.5. In Silico Screening for Secondary Metabolite Production in Strains OE54 and OE57^T^

The biosynthetic potential of both strains was assessed through a bioinformatic analysis of their genome sequences using the antiSMASH software. In the genome of strain OE54, a total of 51 predicted BGCs were identified (Figure 4). Among these, eight BGCs were predicted to encode compounds with AF activity. Notably, four BGCs exhibited a high sequence identity (>60%) to known AF biosynthetic pathways. These included the following: (i) a BGC with a 96% sequence identity to neomediomycin B, a polyketide-class aminopolyol compound that is an analog of mediomycin (also known as clethramycin), a known AF agent [68]; (ii) a BGC with a 95% sequence identity to azalomycin F3a, a 36-membered polyhydroxyl macrolide with demonstrated AF activity against various fungal phytopathogens [69]; (iii) a BGC with a 82% sequence identity to rapamycin, a potent AF compound effective against multiple fungal phytopathogens [70,71], including *V. dahliae*, which exhibits hypersensitivity to this compound [72] (*S. iranensis* is one of only two known *Streptomyces* species capable of producing rapamycin [73]); and (iv) a BGC with a 66% sequence identity to nigericin, a polyether compound with weak AF activity that, nonetheless, enhances the AF activity of rapamycin [74]. Additional putative BGCs associated with AF activity were identified, albeit with lower sequence identity. These included the following: rustmicin (33%), a 14-membered macrolide that inhibits sphingolipid synthesis [75]; the polyene macrolactam BE-14106 (28%) [76]; and notonesomycin (11%) [77].

A total of 52 predicted biosynthetic gene clusters (BGCs) were identified in the genome of strain OE57^T^ (Figure 4), five of which were predicted to encode potential AF compounds. Among these, three BGCs exhibited a sequence similarity greater than 60% to known AF biosynthetic pathways: azalomycin F3a (86%), rapamycin (82%), and nigericin (66%). Additional putative BGCs with a lower sequence similarity included toyocamycin (30%) [78] and notonesomycin (11%).

The identification of a putative rapamycin-encoding BGC in the genome of strain OE57^T^ was particularly noteworthy, as the production of this compound has only been experimentally confirmed in two *Streptomyces* species: *S. iranensis* and *S. rapamycinicus*. To further investigate this finding, a comparative analysis was conducted to examine the organization of the rapamycin BGC among *S. rapamycinicus* NRRL 5491^T^ [79], *S. iranensis* DSM 41954^T^ (complete genome annotation from October 2024, available at https://www.ncbi.nlm.nih.gov/datasets/genome/GCF_042466515.1/), and the OE54 and OE57^T^ strains (Figure 5).

The comparative analysis of rapamycin BGCs from *Streptomyces rapamycinicus* (A), *Streptomyces iranensis* (C), and the strains OE57^T^ (B) and OE54 (D) (Figure 5) revealed both conserved and strain-specific features, potentially reflecting evolutionary adaptations that influence rapamycin biosynthesis. The overall organization of the rapamycin BGC is highly conserved across the four strains, with the core genes *rapB*, *rapA*, and *rapC*, encoding the multifunctional polyketide synthase (PKS), as well as the NRPS-like gene *rapP*, maintaining a consistent arrangement.

However, minor structural differences were observed: (a) The BGC organization in *S. iranensis* and strain OE54 was nearly identical, except for the presence of a small open reading frame, *orf5*, in *S. iranensis* (Figure 5). This gene encodes a hypothetical protein and is absent in the OE54 BGC, as well as in the *S. rapamycinicus* and OE57^T^ clusters. (b) The genes *rapO, rapN, rapM, rapL, rapK, rapJ, rapI, rapH*, and *rapG*, which are involved in regulation, precursor synthesis, and macrolactone tailoring, are located downstream of the PKS core region in *S. rapamycinicus* and strain OE57^T^. In contrast, in *S. iranensis* and strain OE54, *rapG* has been repositioned to the right end of the cluster (Figure 5). (c) The most pronounced differences were observed upstream of the PKS core region. In *S. rapamycinicus* and OE57^T^, the genes *rapS, rapR, rapV, rapW*, and *rapX* are organized in an identical manner. However, the *rapY, rapZ,* and *rapZZ* genes, which have unknown functions, are absent in the OE57^T^ cluster. Similarly, the *rapT* and *rapU* genes, located between *rapB* and *rapS* in *S. rapamycinicus*, are missing in OE57^T^. The *rapY* encodes a protein homologous to antibiotic export repressors, such as ActII in the actinorhodin cluster and TcmR in the tetracenomycin cluster (Supplementary Information Table S1) [80]. It negatively regulates the expression of most rapamycin biosynthetic genes [81]. In contrast, in the OE57^T^ cluster, three open reading frames (*orf2, orf3,* and *orf4*) occupy the region between *rapB* and *rapS*. *orf2* encodes a TetR/AcrR family transcriptional regulator with >99% similarity to homologs found in various *Streptomyces* species. These regulators typically function as repressors involved in antibiotic resistance and the regulation of small-molecule exporters [82], suggesting that *orf2* in OE57 may serve a regulatory role analogous to *rapY* in *S. rapamycinicus*. *orf3* encodes a protein with >90% similarity to *O*-methyltransferases, including acetylserotonin *O*-methyltransferases, while *orf4* encodes a hypothetical protein with no database homologs. (d) In *S. iranensis* and strain OE54, three ORFs (*orf6, orf7,* and *orf8*) are located between *rapB* and *rapS*. *orf6* encodes a protein belonging to the SGNH/GDSL hydrolase family, *orf7* encodes a putative FAD-dependent mono-oxygenase, and *orf8*—similar to *orf2* in *S. rapamycinicus* and OE57—encodes a protein related to TecR/AcrR family regulators. (e) Finally, attention should be drawn to the *orf1* gene, which encodes a protein with a high amino acid identity to *orfDD*. Both genes are predicted to encode a pyridoxal phosphate-dependent cystathionine synthase. In *S. rapamycinicus* and the OE57^T^ strain, this gene is located outside the rapamycin cluster, with its right boundary defined by *orfDD*, which, according to Molnár et al. (1996) [83], also lies outside the cluster. In contrast, in *Streptomyces iranensis* and the OE54 strain, *orf1* is positioned between *rapD* and *rapDD* (Supplementary Information, Figure S3). In *S. rapamycinicus*, a homologous *orf1* is situated approximately 10 kb downstream of the rapamycin BGC, close to the actinoplanic acid BGC. Notably, in this species, the actinoplanic acid BGC is located immediately downstream of the rapamycin cluster [84]. While actinoplanic acid BGCs are also present in *S. iranensis*, OE54, and OE57^T^, in these strains, they are positioned at a considerable genomic distance (~8.5–9 Mb) from the rapamycin cluster (Supplementary Information, Figure S3).

### 3.6. Identification of Rapamycin as the Main Antifungal Compound Produced by OE54 and OE57^T^ Strains

**Figure 6:**
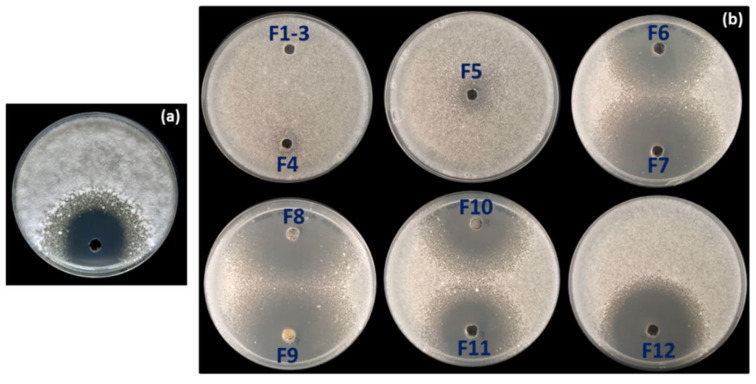
(**a**) Antifungal activity against *Verticillium dahliae* detected in the supernatant of the fermentation broth of strain OE54 after 72 h of growth, and (**b**) in the 12 fractions (F1–F12) obtained from the initial round of vacuum flash chromatography (VFC) during fractionation of the fermentation broth. Antifungal activity was observed in fractions F6–F12, with the strongest inhibition detected in fractions F7–F9.

To identify the compound(s) responsible for the AF activity observed in strain OE54, a total of 6.5 L of fermentation broth was obtained using the ISP2 medium following incubation for 72 h at 28 °C and 200 rpm. Optimal AF production was confirmed through a bioassay using the culture broth against *Verticillium dahliae* (Figure 6a). Subsequently, an activity-guided vacuum flash chromatography (VFC) was performed, yielding 2.90 g of extract. A 2 mg sample of this extract was analyzed by liquid chromatography–mass spectrometry (LC-MS) and tested for AF activity via a plate bioassay. Following VFC, 12 fractions were collected and subjected to AF activity testing using a plate bioassay. AF activity was detected in fractions 6–12, with the most pronounced inhibition observed in fractions 7–9 (Figure 6b).

**Figure 7:**
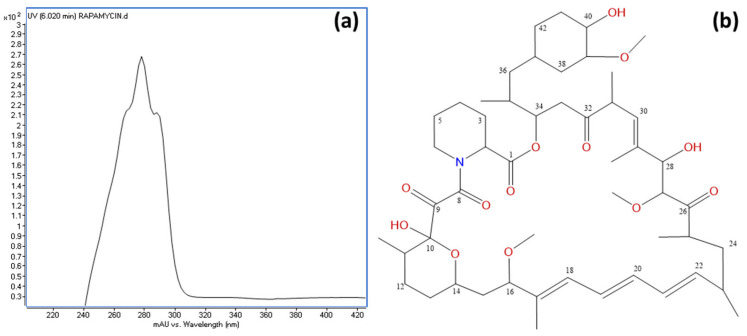
(**a**) UV spectrum of rapamycin, displaying absorption maxima at 267, 277, and 288 nm; and (**b**) chemical structure of rapamycin with carbon positions numbered as determined by NMR analysis.

An HPLC and LC/MS analysis of the active fractions revealed a common major peak. This peak was scarcely detected in the diode array detector (DAD) at λ = 220 nm due to the compound’s UV spectrum, which exhibited absorption maxima at 267, 277, and 288 nm (Figure 7a). However, it was clearly visible in the evaporative light-scattering detector (ELSD) chromatogram (Supplementary Information Figure S4). Active fraction 9 (429.0 mg) was further purified using C18 reversed-phase column chromatography, yielding 17.2 mg of a pure active compound. High-resolution electrospray ionization mass spectrometry (HRESIMS) analysis determined the molecular formula as C_51_H_79_NO_13_, indicating 13 degrees of unsaturation (Supplementary Information Figure S4). Structural elucidation was performed using extensive NMR spectroscopy, including ^1^H NMR, ^13^C NMR, ^1^H-^1^H COSY, gHSQC, and gHMBC experiments, confirming the compound as rapamycin (Figure 7b). The spectroscopic data were consistent with previously reported values [85]. ^1^H-NMR and ^13^C-NMR data are provided in Supplementary Information Table S2.

The presence of a putative rapamycin BGC in the genome of strain OE57^T^ suggested that the strong AF activity observed against *V. dahliae* could be attributed to the production of this compound. To test this hypothesis, liquid cultures were grown in ISP2 medium, and the culture broth was extracted with ethyl acetate. The solvent was then evaporated, and the residue was dissolved in 80% methanol for HPLC analysis. The HPLC chromatogram revealed a peak with a retention time of 16.3 min, identical to that of a pure rapamycin standard. Additionally, the UV absorption spectrum of the detected compound matched that of rapamycin. These results confirm that rapamycin is responsible for most of the AF activity exhibited by strain OE57^T^.

### 3.7. Identification of the Epoxy Cinnamoyl-Containing Nonribosomal Peptide Epoxinnamide Produced by OE54

**Figure 8:**
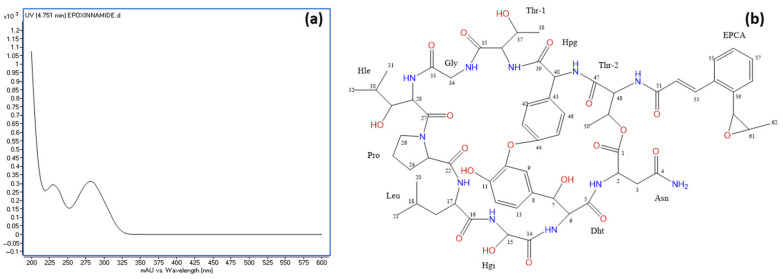
(**a**) UV spectrum of epoxinnamide and (**b**) chemical structure of epoxinnamide with carbon positions labeled as determined by NMR analysis. Abbreviations: Dht, 3β-dihydroxytyrosine; Hgy, α-hydroxyglycine; Hle, β-hydroxyleucine; Hpg, hydroxyphenylglycine; EPCA, *O*-1,2-epoxypropyl cinnamic acid.

During the purification of rapamycin from the fermentation broth, a second round of VFC was applied to the combined fractions F10–F12 (see Figure 6b). Notably, fraction F8′ contained 14.7 mg of a highly pure compound, as confirmed by HPLC with ELSD detection (Supplementary Information Figure S5). This compound, identified as epoxinnamide, exhibited no AF activity and displayed a UV absorption peak at 280 nm (Figure 8a). An HRESIMS analysis determined the molecular formula of epoxinnamide as C_62_H_79_N_11_O_20_, corresponding to 29 degrees of unsaturation (Supplementary Information Figure S5). Structural elucidation using ^1^H and ^13^C NMR spectroscopy (Figure 8b) confirmed its identity, with spectroscopic data consistent with previously reported values [86]. The corresponding ^1^H-NMR and ^13^C-NMR data are provided in Supplementary Information Table S3.

Epoxinnamide is an epoxy cinnamoyl-containing nonribosomal peptide (NRP) that has, to date, only been reported in a *Streptomyces* sp. strain isolated from intertidal mudflats in Oido, Republic of Korea. This finding establishes strain OE54 as the second known producer of this compound. Furthermore, epoxinnamide BGCs have been identified in the genomes of *S. iranensis*, *S. rapamycinicus*, OE54, and OE57^T^. The overall organization of the epoxinnamide BGC is highly conserved in *Streptomyces* sp. OID44, where it was first described [86], as well as in *S. rapamycinicus* and strain OE57^T^. However, notable differences in the gene arrangement were observed in *S. iranensis* and strain OE54 BGCs (Supplementary Information Figure S6).

### 3.8. Effect of Rapamycin on V. dahliae Conidiospore Germination, Radial Growth Inhibition, and MIC Determination

Rapamycin effectively inhibited the germination of *Verticillium dahliae* V937I conidiospores within the tested concentration range of 5–125 μg/mL. A probit analysis of the germination data produced a regression equation of *y* = 0.6649*x* + 3.7187 (*R^2^* = 0.9175), allowing for the calculation of the LC_50_ (lethal concentration for 50% inhibition) as 87.36 μg/mL (Supplementary Information Figure S7).

Rapamycin exhibited significantly greater potency in inhibiting the radial growth of *V. dahliae*, with detectable effects at concentrations as low as 2 ng/mL. The IC_50_ (half-maximal inhibitory concentration) was determined to be 3.91 ng/mL.

### 3.9. Cultural, Morphological, and Phenotypic Properties of OE54 and OE57 Strains

Strains OE54 and OE57^T^ exhibit phenotypic and morphological features consistent with their classification within the genus *Streptomyces* [66]. As summarized in Supplementary Information Table S4, OE54 and *S. iranensis* demonstrated growth within a temperature range of 15–37 °C, whereas *S. rapamycinicus* and OE57^T^ were capable of growth at temperatures ranging from 15 °C to 42 °C. Regarding pH tolerance, *S. iranensis* and OE57^T^ were able to grow in a pH range of 5–10, while OE54 and *S. rapamycinicus* exhibited a broader tolerance of pH 5–12, though the growth of *S. rapamycinicus* was weak at pH 12. Differences in salt tolerance were also observed: *S. iranensis* and OE54 exhibited low salt tolerance, growing only in the presence of 2.5% NaCl, whereas *S. rapamycinicus* and OE57^T^ were able to grow in up to 5.0% NaCl. None of the strains were capable of growth at 7.5% NaCl.

The strains also displayed distinct growth characteristics on various culture media (Supplementary Information Table S4). All strains exhibited moderate to good growth on ISP 1, ISP 2, ISP 3, ISP 4, Bennet, R5, and GYM agar media (growth characteristics of *S. iranensis* and *S. rapamycinicus* type strains are available at https://www.dsmz.de/collection/catalogue). Growth on TSA was weak for all strains except for OE57^T^, which exhibited moderate growth. In ISP 5, *S. iranensis* demonstrated moderate growth, whereas all other strains displayed weak growth. In ISP 6, all strains exhibited weak growth.

Biochemical and enzymatic properties further differentiate these strains from one another and from their closest phylogenomic relatives (Supplementary Information Table S5). Notably, OE54 and *S. iranensis* differ in traits such as urea degradation and the production of esterase C4 and α-glucosidase, which are absent in the reference strain *S. iranensis* DSM 41954^T^. Given that phylogenetic analyses clearly classify OE54 within the *S. iranensis* species, these differences may reflect environmental adaptation. While the *S. iranensis* type strain was isolated from rhizosphere soil at a depth of 10 cm with no indication of the associated plant [87], OE54 was isolated as an endophyte from the interior of olive roots. Similarly, OE57^T^ can be distinguished from the reference strains of *S. iranensis* DSM 41954^T^ and *S. rapamycinicus* DSM 41530^T^ by the absence of enzymatic activities such as C8 and C14 lipases, α-galactosidase, and α-mannosidase (Supplementary Information Table S5).

### 3.10. Chemotaxonomic Features

The chemotaxonomic feature of strain OE57^T^ were in concordance with those of the genus *Streptomyces* [66]. Whole-organism hydrolysates of strain OE57^T^ and its close phylogenomic neighbor *Streptomyces rapamycinicus* DSM 41530^T^ were rich in *LL*-diaminopimelic acid in the cell-wall peptidoglycan and have glucose and ribose as whole-cell sugars. The strain contained diphosphatidylglycerol (DPG), phosphatidylethanolamine (PE), hydroxy phosphatidylethanolamine (OH-PE), phosphatidylinositol (PI), phospholipid (PL), aminophospholipid (APL), and unidentified lipids (Ls), while strain DSM 41530^T^ had phosphatidylmethylethanolamine (PME) and glycophospholipid (Supplementary Information Figure S8). The major fatty acids (>10%) of strain OE57^T^ contained *iso*-C_15:0_, C_16:0_, and *iso*-C_17:0_, while strain DSM 41530^T^ showed *iso*-C_15:0_, *anteiso*-C_15:0_, *iso*-C_16:0_, and C_16:0_ (Supplementary Information Table S6). The predominant menaquinone (>5%) of strains OE57^T^ and DSM 41530^T^ consisted of MK9-(H4), MK9-(H6), MK9-(H8), and MK-10(H6).

### 3.11. Description of Streptomyces lacaronensis sp. nov.

*Streptomyces lacaronensis* (la.ca.ro.nen’sis N.L. gen. N. *lacaronensis*, referring to river Lacarón, on whose bank the type strain was isolated from the roots of an olive tree, Extremadura region, Spain):

The strain is a Gram-stain-positive, aerobic, non-motile bacterium that forms light grey aerial mycelium on oat meal (ISP 3) agar and signal white aerial mycelium in starch-mineral (ISP 4), ISP 1, ISP 2, ISP 5, ISP 6, NA, and GYM agars. A salmon range diffusible pigment is produced in ISP 7 media at 28 °C. The strain is able to grow from 15 °C to 42 °C, optimally at 28 °C, and from pH 5.0–10.0, optimally at pH 7.0. Additional cultural and morphological properties are mentioned in Supplementary Tables S4 and S5.

The strain has LL-diaminopimelic acid of the cell wall peptidoglycan and glucose and ribose as whole cell sugars. It contained diphosphatidylglycerol (DPG), phosphatidylethanolamine (PE), hydroxy phosphatidylethanolamine (OH-PE), phosphatidylinositol (PI), phospholipid (PL), aminophospholipid (APL), and unidentified lipids (Ls). The fatty acid profile consists of (>5%) *iso*-C_15:0_, *anteiso*-C_15:0_, C_16:1_ *cis* 9, *iso*-C_16:0,_ C_16:0_, *iso*-C_17:1_ *cis* 9, and *iso*-C_17:0_. It has MK9-(H4), MK9-(H6), MK9-(H8), and MK-10(H6) as predominant menaquinone.

The genome size of the strain is 12.29 Mbp, and its in silico G + C content is 70.9%. The type strain OE57^T^ (=DSM 118741^T^ = CECT 31164^T^) was isolated from inside a sample of an olive root collected from a tree exhibiting visual symptoms compatible with Verticillium wilt that was located on a commercial plot located in the town of La Garrovilla (Spain) and at geographic coordinates 38°55′08.1” N 6°31′27.7” W. The genome sequence of OE57^T^ strain has been deposited in the GenBank database under the accession number JBLHDK000000000. The BioProject accession number is PRJNA1185486.

## 4. Discussion

The genus *Streptomyces* represents one of the most complex and diverse bacterial taxa characterized to date, comprising over 755 validly named species (https://lpsn.dsmz.de/genus/streptomyces; accessed on 1 June 2025), with this number continually increasing as new species are discovered. A defining evolutionary feature of *Streptomyces* species is their large genome size relative to other bacterial groups, which encodes the biosynthetic pathways for numerous secondary metabolites with significant industrial applications. Among these metabolites, many exhibit potent AF properties, making *Streptomyces* particularly attractive candidates for use as BCAs in the management of fungal phytopathogens affecting a wide range of cultivated plants.

In a screening study aimed to isolate *Streptomyces* strains from the interior root tissues of olive plants, two strains, OE54 and OE57^T^, were identified based on their strong in vitro AF activity against *V. dahliae*. Molecular, physiological, and growth analyses confirmed that strain OE54 belongs to *S. iranensis*, whereas strain OE57^T^ represents a novel species, herein designated *S. lacaronensis*. The discovery of a new *Streptomyces* species is inherently valuable, as it contributes to a more comprehensive understanding of the genus. However, a particularly noteworthy finding of this study was that both *S. iranensis* OE54 and *S. lacaronensis* OE57^T^ exhibited pronounced AF activity attributable to rapamycin production.

The simultaneous isolation of two distinct rapamycin-producing *Streptomyces* species from the same plant tissue is highly unusual, as the production of this compound has been reported in only a limited number of bacterial species to date. This finding suggests that olive trees affected by Verticillium wilt may recruit rapamycin-producing *Streptomyces* strains from the rhizosphere, enabling their establishment as endophytes. Such recruitment could confer advantages to the host plant, including the production of antifungal compounds that may mitigate the impact of pathogenic infections. Previous research has indicated that the rhizosphere of hop plants affected by Verticillium wilt undergoes significant shifts in microbial composition compared to that of healthy plants, specifically characterized by the recruitment of putatively beneficial fungi [88]. This phenomenon of attracting potentially beneficial microorganisms to the rhizosphere of plants afflicted by phytopathogenic fungi may facilitate subsequent colonization by those capable of adopting an endophytic lifestyle.

Prior to this study, rapamycin biosynthesis had been documented exclusively in *Streptomyces rapamycinicus* (formerly *S. hygroscopicus*) [89], *S. iranensis* [73], and *Actinoplanes* sp. strain N902-109 [90]. The detection of rapamycin in the fermentation broths of *S. iranensis* OE54 and *S. lacaronensis* OE57^T^ expands this previously limited list, underscoring the potential ecological significance of rapamycin production in endophytic *Streptomyces* strains. This finding is particularly noteworthy, as rapamycin—often referred to as a “billion-dollar molecule”—is the second most widely used immunosuppressant of microbial origin, following cyclosporine [91]. Initially regarded as a low-toxicity AF compound, its potent immunosuppressive properties were identified in 1977 [92], leading to its widespread clinical application in organ transplantation and autoimmune disease management. Moreover, rapamycin has since been recognized for its broad therapeutic potential, including antitumor activity [93], neuroprotective effects in neurodegenerative diseases [94,95], and its possible role in lifespan extension [96].

The molecular target of rapamycin in eukaryotic cells is well-established. Rapamycin interacts with FK506-binding protein 12 (FKBP12), forming a complex that inhibits the activity of the Target of Rapamycin (TOR) kinase. TOR is an evolutionarily conserved phosphoinositide 3-kinase (PI3K)-related protein kinase that regulates numerous cellular processes in response to diverse extracellular and intracellular signals [72,97]. In this study, we demonstrate that *V. dahliae* exhibits a high degree of sensitivity to rapamycin, which markedly suppresses hyphal growth and significantly inhibited conidiospore germination. These findings align with the results of Li et al. (2019) [72], who reported that rapamycin effectively inhibits ribosomal biogenesis, RNA polymerase II transcription factors, and various metabolic pathways, including the transcription of genes encoding cell-wall-degrading enzymes in *V. dahliae*-treated cells. Moreover, their study confirmed that rapamycin substantially reduces the pathogenicity of *V. dahliae*, thereby preventing the onset of Verticillium wilt [72]. Collectively, these findings suggest that rapamycin may serve as a promising biofungicide, offering a viable alternative to conventional chemical fungicides for the management of Verticillium wilt. Furthermore, rapamycin-producing *Streptomyces* spp. strains, such as those identified in this study, hold significant potential as effective BCAs.

A comparative analysis of the BGCs associated with rapamycin production in *S. rapamycinicus*, *S. iranensis*, and strains OE54 and OE57^T^ reveals both conserved and strain-specific features, reflecting potential evolutionary adaptations that influence rapamycin biosynthesis. As illustrated in Figure 5, the BGCs of *S. iranensis* and strain OE54 exhibit a high degree of similarity, with the sole distinction being the presence of a small open reading frame (*orf5*) located between *rapD* and *rapE* in *S. iranensis*, which is absent in the OE54 cluster. *orf5* encodes a small hypothetical protein with no significant homologs in existing databases, suggesting that its presence or absence may stem from a sequencing artifact in one of the genomes. The close similarity between these BGCs further supports the classification of strain OE54 as *S. iranensis*.

The core biosynthetic genes (*rapA*, *rapB*, *rapC*, and *rapP*) are highly conserved across all four clusters, underscoring their essential role in rapamycin biosynthesis. Additionally, the downstream flanking region encompassing *rapQ*, *rapO*, *rapN*, *rapM*, *rapL*, *rapK*, *rapJ*, *rapI*, and *rapH* exhibits substantial conservation, suggesting that these genes play crucial roles in rapamycin production (see Supplementary Table S1 for a description of their putative functions). Previous studies have demonstrated that *rapG* and *rapH* function as positive regulators of rapamycin biosynthesis in *S. rapamycinicus* [98]. Furthermore, *rapI*, *rapM*, and *rapQ* encode S-adenosylmethionine (SAM)-dependent *O*-methyltransferases [83], which are likely responsible for the *O*-methylation of positions C16, C27, and C39 of the rapamycin molecule (Figure 7). In contrast, the products of *rapJ* and *rapN* (encoding cytochrome P450 mono-oxygenases) and *rapO* (encoding a ferredoxin-like protein) [83,99] are predicted to mediate the formation of keto groups at positions C9, C27, and, potentially, C32 (Figure 7). Lastly, *rapL* encodes a lysine cyclodeaminase, an enzyme that catalyzes the cyclization of lysine to generate L-pipecolate, a direct precursor in rapamycin biosynthesis [83,100].

A notable degree of variability is observed in the organization of genes located downstream of *rapH*, using the *S. rapamycinicus* BGC as a reference. In both *S. rapamycinicus* and strain OE57^T^, this region maintains a conserved gene arrangement, comprising *rapG, rapF, orfE,* and *orfD*, with *orfDD* positioned outside the cluster, as reported by Molnár et al. (1996) [83]. In contrast, *S. iranensis* and strain OE54 exhibit an inverted gene organization, with *orfDD, orf1, orfD, orfE, rapF,* and *rapG* positioned downstream of *rapH*. This rearrangement suggests a potential inversion event affecting a DNA fragment containing these genes. The presence of *orf1* within the *S. iranensis*, and OE54 and OE57 BGCs is particularly noteworthy. This gene encodes a protein exhibiting a 92.12% amino acid identity with *orfDD*. An InterPro analysis indicates that both *orf1* and *orfDD* contain conserved domains characteristic of cysteine synthase/cystathionine β-synthase (IPR050214) (see Supplementary Table S1). Additionally, *orf1* shares a homology with a 2,3-diaminopropionate biosynthesis protein (SbnA), a homologous enzyme identified in multiple *Streptomyces* species. The biological function of SbnA has been extensively studied in *Staphylococcus aureus*, where it participates in the biosynthesis of L-2,3-diaminopropionic acid (L-DAP), a precursor for siderophores and antibiotics [101]. Specifically, SbnA catalyzes the condensation of O-phospho-L-serine and L-glutamate to produce N-(1-amino-1-carboxyl-2-ethyl)-glutamic acid, a direct precursor of L-DAP. Interestingly, in *S. rapamycinicus*, *orf1* is located approximately 10 kb downstream from the right boundary of the rapamycin BGC (see Supplementary Figure S2). Despite its genomic proximity, no direct evidence currently supports its involvement in rapamycin biosynthesis. However, its presence within or near this biosynthetic cluster, along with its high sequence similarity to *orfDD* and its conserved domain architecture, raises intriguing questions regarding its potential evolutionary, regulatory, or functional significance. Further investigations are warranted to elucidate its precise role in rapamycin biosynthesis and its broader physiological implications.

The genomic region upstream of *rapB*, which encompasses regulatory and accessory genes associated with rapamycin biosynthesis, exhibits considerable variation among the analyzed strains. In *Streptomyces rapamycinicus*, this region includes *rapT, orfU, rapS, rapR, orfV, orfW, rapX, rapY,* and *rapZ*, with *orfZZ* positioned outside the cluster, as previously described by Molnár et al. (1996) [83]. Notably, *rapS, rapR, orfV, orfW,* and *rapX* are universally conserved across all four strains (Figure 5). The co-occurrence of *orfV, orfW,* and *rapX* suggests that these genes may form an operon involved in rapamycin export, given their homology with known transport proteins (see Supplementary Table S1) [83]. However, *rapT* and *orfU* are absent in *S. iranensis* and strains OE54 and OE57^T^, indicating that these genes may not be essential for rapamycin biosynthesis. This finding is particularly significant given that *rapT* encodes a putative ketoreductase implicated in the synthesis of the dihydroxycyclohexane carboxylic acid starter unit of rapamycin [83]. Similarly, *rapY* and *rapZ* are absent from the *S. iranensis*, OE54, and OE57^T^ clusters. The absence of *rapY* is especially notable, as this gene has been identified as a negative regulator of rapamycin biosynthesis, alongside *rapR* and *rapS* [81]. In *S. rapamycinicus*, the overexpression of *rapY, rapR,* or *rapS* results in a marked reduction in rapamycin production, further confirming their role as negative regulators [81]. Interestingly, *rapY* encodes a protein with a high sequence identity to members of the TetR family of transcriptional regulators. Its regulatory function may be compensated by *orf2* in strain OE57^T^ and *orf8* in *S. iranensis* and OE54, as these genes encode putative TetR/AcrR family transcriptional regulators. Additionally, the absence of *orfZ*, which encodes a hypothetical protein with no significant sequence similarity to known functional domains (see Supplementary Table S1), suggests that it may not play a crucial role in rapamycin biosynthesis.

The OE57^T^ strain harbors three unique genes within its rapamycin BGC —*orf2, orf3,* and *orf4*— that are absent in the other analyzed clusters. Notably, *orf3* encodes a protein with homology to various methyltransferases (see Supplementary Table S1), suggesting a potential role in the site-specific methylation of the rapamycin structure. Meanwhile, *orf2* encodes a putative transcriptional regulator belonging to the TetR/AcrR family, which may influence rapamycin biosynthesis. The *orf4* gene product exhibits a similarity to protein kinase domain-containing proteins, implying a possible role in regulatory pathways controlling rapamycin production. Similarly, the *S. iranensis* and OE54 clusters contain three unique genes *orf6, orf7,* and *orf8*. *orf8* encodes another TetR/AcrR family regulator, likely contributing to the transcriptional control of rapamycin biosynthesis in these strains. The *orf7* gene product shares a similarity with FAD-dependent mono-oxygenases, suggesting a potential involvement in hydroxylation reactions that could modify the rapamycin structure. Lastly, *orf6* encodes a protein belonging to the SGNH/GDSL hydrolase family, which is known to participate in diverse biological processes, including lipid metabolism and secondary metabolite biosynthesis. The considerable genetic variability observed in the left-flanking region of the rapamycin BGC across these strains suggests that these additional genes may contribute to the biosynthesis of structurally distinct rapamycin analogs (rapalogs) or modulate biosynthetic regulatory mechanisms. These variations likely reflect diverse ecological adaptations among rapamycin-producing *Streptomyces* species and warrant further investigation to elucidate their functional significance.

Finally, we would like to emphasize that this comparative genomic analysis provides valuable insights into the conservation and diversification of rapamycin biosynthesis among different *Streptomyces* species and highlights the genetic basis underlying its production in strains OE54 and OE57^T^. These variations in gene content and organization highlight potential regulatory adaptations in rapamycin-producing *Streptomyces* species and suggest functional redundancy, or alternative regulatory mechanisms that warrant further investigation.

Beyond the rapamycin BGC, we analyzed the clusters responsible for actinoplanic acid and epoxinnamide biosynthesis. Notably, neither cluster was directly identified by antiSMASH. Instead, their classification was inferred based on literature comparisons and detailed analyses of sequence homology and synteny. Our findings indicate that the epoxinnamide cluster belongs to the non-ribosomal peptide synthetase (NRPS) family with arylpolyene-like features, exhibiting 50% similarity to kitacinnamycin. Meanwhile, the actinoplanic acid cluster is classified as NRPS-like, contains a Type I polyketide synthase (T1PKS) module, and shares 20% similarity with lydicamycin.

The genetic organization of the epoxinnamide BGC revealed a conserved core region (*epcA–epcE*) across all analyzed strains, though substantial differences were observed in the flanking biosynthetic, regulatory, and transport-associated genes. In *S. rapamycinicus* and OE57^T^, the cluster retains a compact and continuous structure, with regulatory genes (*epcB* and *epcC*) and transport-related genes (*epcF* and *epcG*) positioned adjacent to the core biosynthetic genes. In contrast, *S. iranensis* and OE54 display notable gene losses and rearrangements, particularly in the upstream region, where genes 1–5 are either absent or fragmented (Supplementary Information Figure S6). Furthermore, while the only previously published study on epoxinnamide described two separate core genes (*epcD* and *epcE*), our analysis of two newly sequenced strains alongside two reference strains consistently identified a single gene in this region, which we have designated *epcDE* (Supplementary Information Figure S6). This discrepancy may arise from differences in the annotation criteria, the natural variation in gene organization, or potential sequencing and assembly artifacts. Additionally, the observed variation in regulatory gene organization across these strains suggests potential differences in expression control mechanisms, which could influence metabolite production.

A notable feature of the actinoplanic acid BGC is its genomic context across the analyzed *Streptomyces* strains. In *S. rapamycinicus*, the APL cluster is co-localized with the rapamycin BGC, suggesting a potential evolutionary and functional interdependence between the two pathways [84]. However, in *S. iranensis*, OE54, and OE57^T^, the APL and rapamycin clusters are situated in opposite chromosomal regions. Despite this spatial separation, the APL cluster remains highly conserved in both gene content and organization across these strains (Supplementary Information Figure S3). This genomic dispersion, previously reported in *S. iranensis* [84], extends to OE54 and OE57^T^, suggesting that such rearrangements are not isolated events but may represent a broader evolutionary trend. Interestingly, actinoplanic acid was not detected in the chemical analyses of OE54 and OE57^T^ fermentation broths. While the genomic separation of regulatory elements, such as aplR, from the core biosynthetic genes might suggest transcriptional uncoupling, Mrak et al. (2018) [84] demonstrated that *aplR* deletion in *S. rapamycinicus* did not abolish APL production. This finding could imply the existence of alternative regulatory mechanisms that maintain pathway expression despite genomic separation. Moreover, the mere presence of biosynthetic genes does not necessarily indicate active expression, as regulatory control is complex and influenced by strain-specific and environmental factors. The absence of APL production under the tested conditions may reflect these intricate regulatory dynamics.

These findings underscore the complexity and plasticity of secondary metabolism in *Streptomyces*. The structural variability observed among these BGCs, likely driven by genomic rearrangements, highlights the limitations of annotation tools such as antiSMASH when analyzing clusters not well-represented in reference databases. Consequently, integrating comparative genomics with experimental validation is essential for fully exploring the biosynthetic potential of these strains. Future studies employing transcriptomic and metabolomic approaches will be crucial to elucidating how these genetic differences translate into metabolite diversity, potentially leading to the discovery of novel bioactive compounds.

## 5. Conclusions

Root tissues from olive trees affected by Verticillium wilt have proven to be a valuable reservoir for the isolation of endophytic *Streptomyces* strains. More than 25% of the isolates exhibited some antifungal activity against *V. dahliae*. Among these, two isolates—*Streptomyces iranensis* OE54 and *Streptomyces lacaronensis* OE57—exhibited the highest levels of growth inhibition. Both strains were able to show strong antifungal activity against *V. dahliae* in both in vitro and small-scale soil bioassays. This activity could be attributed to the production of rapamycin, a compound that effectively inhibited conidial germination and was particularly potent in suppressing the mycelial growth of *V. dahliae* at concentrations within the nanograms per mililiter range. *Streptomyces lacaronensis* sp. nov. OE57^T^ has been formally described as a new species within the genus *Streptomyces* and recognized as a new producer of rapamycin.

## Figures and Tables

**Table 1:** GenBank accession numbers of DNA sequences corresponding to partially sequenced genes used in the MLSA analysis of the *Streptomyces* sp. OE54 and OE57^T^ strains.

Strain	GenBank Accession Numbers					
	*16S rRNA*	*atpD*	*gyrB*	*recA*	*rpoB*	*trpB*
OE54	PP980539	PP979823	PP979829	PP979835	PP979841	PP979847
OE57^T^	PP980540	PP979824	PP979830	PP979836	PP979842	PP979848

**Table 2:** Identification of *Streptomyces* strains OE54 and OE57^T^ based on MLSA analysis and their MLSA evolutionary distances from phylogenetically related strains.

MLSA (Kimura 2-Parameter) Distance *		
Strain	*Streptomyces* sp. OE54	*Streptomyces* sp. OE57^T^
*Streptomyces* sp. OE57^T^	0.021	
*Streptomyces iranensis* HM 35^T^	0.005	0.022
*Streptomyces rapamycinicus* NRRL 5491^T^	0.018	0.011
*Streptomyces javensis* DSM 41764^T^	0.018	0.014

* Strain pairs having a ≤0.007 MLSA evolutionary distance were considered conspecific based on the guideline empirically determined by Rong and Huang (2012) [38].

## Data Availability

The original contributions presented in this study are included in the article/Supplementary Materials. Further inquiries can be directed to the corresponding authors.

## Supplementary Materials

The following supporting information can be downloaded at: https://www.mdpi.com/article/10.3390/microorganisms13071622/s1, Figure S1: *Streptomyces* phylogenetic tree inferred from concatenated partial sequences of the housekeeping genes (*atpD*, *gyrB*, *recA*, *rpoB*, and *trpB*) of endophytic OE54 and OE57^T^ strains with type strains obtained from the ARS Microbial Genomic Sequence Database server; Figure S2: Maximum likelihood phylogenetic tree based on 16S rRNA gene sequences, showing the relationships between the OE54 and OE57^T^ strains and their closely related species; Figure S3: Genetic organization of the rapamycin and actinoplanic acid biosynthetic gene clusters (BGCs) in *S. rapamycinicus* DSM 41530^T^ (a), OE57^T^ (b), *S. iranensis* DSM 41954^T^ (c), and OE54 (d) genomes; Figure S4: High-performance liquid chromatography (HPLC) chromatogram of fraction F9, obtained via vacuum flash chromatography from the fermentation broth of strain OE54, using an evaporative light scattering detector (ELSD) (a) and high-resolution electrospray ionization time-of-flight mass spectrometry (HR ESI-TOF-MS) spectrum of the corresponding compound, identified as rapamycin, with a molecular ion at *m/z* 936.5197 [M + Na]^+^ (b); Figure S5: High-performance liquid chromatography (HPLC) chromatogram of fraction F8, obtained after a second round of flash chromatography from the fermentation broth of strain OE54, using an evaporative light scattering detector (ELSD) (a) and high-resolution electrospray ionization time-of-flight mass spectrometry (HR ESI-TOF-MS) spectrum of the corresponding compound, identified as epoxinnamide, with a molecular ion at *m/z* 1298.5188 [M + H]^+^ (b); Figure S6: Genetic organization of the epoxinnamide cluster in *Streptomyces* sp. OID44 (a), *S. rapamycinicus* DSM 41530^T^ (b), OE57^T^ strain (c), *S. iranensis* DSM 41954^T^ (d), and OE54 strain (e); Figure S7: Probit analysis for germination of conidispores of *V. dahliae* V937I strain after treatment with commercial rapamycin; Figure S8: Two-dimensional TLC plate of polar lipids of strains OE57^T^ and its close phylogenomic relative *Streptomyces rapamycinicus* DSM 41530^T^ stained with molybdatophosphoric acid. Table S1: Inferred functions of *rap* genes and ORFs located in the rapamycin biosynthetic clusters of *S. rapamycinicus* NRRL 5491, *S. iranensis* DSM 41954, OE54, and OE57^T^ strains; Table S2. ^13^C, ^1^H Nuclear Magnetic Resonance (NMR) spectral data of rapamycin [δ (ppm), CDCl_3_]; Table S3: ^13^C, ^1^H Nuclear Magnetic Resonance (NMR) Spectral Data of Eppoxinnamide [δ (ppm), J_HH_ (Hz); CDCl_3_]; Table S4: Cultural and growth characteristics of strains OE54 and OE57^T^, along with their closest phylogenomic relatives; Table S5: Phenotypic characteristics of strains OE54 and OE57^T^, along with their closest phylogenomic relatives, as determined by API 20NE and API ZYM assays; Table S6: Fatty acids profiles of the *Streptomyces lacaronensis* sp. nov. strain OE57^T^ (=DSM 118741^T^) and its closest phylogenomic neighbor *Streptomyces rapamycinicus* DSM 41530^T^. References [81,83,98,99,100,102] are cited in the Supplementary Materials.

## References

[1] Inderbitzin P., Subbarao K.V. (2014). Verticillium Systematics and Evolution: How Confusion Impedes Verticillium Wilt Management and How to Resolve It. Phytopathology.

[2] Klosterman S.J., Atallah Z.K., Vallad G.E., Subbarao K.V. (2009). Diversity, Pathogenicity, and Management of *Verticillium* Species. Annu. Rev. Phytopathol..

[3] Keykhasaber M., Thomma B.P.H.J., Hiemstra J.A. (2018). Verticillium Wilt Caused by *Verticillium dahliae* in Woody Plants with Emphasis on Olive and Shade Trees. Eur. J. Plant Pathol..

[4] Kunej U., Jakše J., Radišek S., Štajner N. (2021). Identification and Characterization of *Verticillium nonalfalfae*-Responsive MicroRNAs in the Roots of Resistant and Susceptible Hop Cultivars. Plants.

[5] López-Escudero F.J., Mercado-Blanco J. (2011). Verticillium Wilt of Olive: A Case Study to Implement an Integrated Strategy to Control a Soil-Borne Pathogen. Plant Soil.

[6] Coque J.J.R., Álvarez-Pérez J.M., Cobos R., González-García S., Ibáñez A.M., Diez Galán A., Calvo-Peña C., Gadd G.M., Sariaslani S. (2020). Advances in the Control of Phytopathogenic Fungi That Infect Crops through Their Root System. Advances in Applied Microbiology.

[7] Mulero-Aparicio A., Agustí-Brisach C., Varo Á., López-Escudero F.J., Trapero A. (2019). A Non-Pathogenic Strain of *Fusarium oxysporum* as a Potential Biocontrol Agent against Verticillium Wilt of Olive. Biol. Control.

[8] Mulero-Aparicio A., Cernava T., Turrà D., Schaefer A., Di Pietro A., López-Escudero F.J., Trapero A., Berg G. (2019). The Role of Volatile Organic Compounds and Rhizosphere Competence in Mode of Action of the Non-Pathogenic *Fusarium oxysporum* FO12 toward Verticillium Wilt. Front. Microbiol..

[9] Varo A., Raya-Ortega M.C., Trapero A. (2016). Selection and Evaluation of Micro-Organisms for Biocontrol of *Verticillium dahliae* in Olive. J. Appl. Microbiol..

[10] Carrero-Carrón I., Trapero-Casas J.L., Olivares-García C., Monte E., Hermosa R., Jiménez-Díaz R.M. (2016). *Trichoderma asperellum* Is Effective for Biocontrol of Verticillium Wilt in Olive Caused by the Defoliating Pathotype of *Verticillium dahliae*. Crop Prot..

[11] Lozano-Tovar M.D., Garrido-Jurado I., Quesada-Moraga E., Raya-Ortega M.C., Trapero-Casas A. (2017). *Metarhizium brunneum* and *Beauveria bassiana* Release Secondary Metabolites with Antagonistic Activity against *Verticillium dahliae* and *Phytophthora megasperma* Olive Pathogens. Crop Prot..

[12] Gómez-Lama Cabanás C., Sesmero R., Valverde-Corredor A., López-Escudero F.J., Mercado-Blanco J. (2017). A Split-Root System to Assess Biocontrol Effectiveness and Defense-Related Genetic Responses in above-Ground Tissues during the Tripartite Interaction *Verticillium dahliae*-Olive-*Pseudomonas fluorescens* PICF7 in Roots. Plant Soil.

[13] Gómez-Lama Cabanás C., Legarda G., Ruano-Rosa D., Pizarro-Tobías P., Valverde-Corredor A., Niqui J.L., Triviño J.C., Roca A., Mercado-Blanco J. (2018). Indigenous *Pseudomonas* spp. Strains from the Olive (*Olea europaea* L.) Rhizosphere as Effective Biocontrol Agents against *Verticillium dahliae*: From the Host Roots to the Bacterial Genomes. Front. Microbiol..

[14] Markakis E.A., Tjamos S.E., Antoniou P.P., Paplomatas E.J., Tjamos E.C. (2016). Biological Control of Verticillium Wilt of Olive by *Paenibacillus alvei*, Strain K165. BioControl.

[15] Cheffi Azabou M., Gharbi Y., Medhioub I., Ennouri K., Barham H., Tounsi S., Triki M.A. (2020). The Endophytic Strain *Bacillus velezensis* OEE1: An Efficient Biocontrol Agent against Verticillium Wilt of Olive and a Potential Plant Growth Promoting Bacteria. Biol. Control.

[16] Calvo-Peña C., Cobos R., Sánchez-López J.M., Ibáñez A., Coque J.J.R. (2023). Albocycline Is the Main Bioactive Antifungal Compound Produced by *Streptomyces* sp. OR6 against *Verticillium dahliae*. Plants.

[17] Díaz-Díaz M., Antón-Domínguez B.I., Raya M.C., Bernal-Cabrera A., Medina-Marrero R., Trapero A., Agustí-Brisach C. (2024). *Streptomyces* spp. Strains as Potential Biological Control Agents against Verticillium Wilt of Olive. J. Fungi.

[18] Seipke R.F., Kaltenpoth M., Hutchings M.I. (2012). *Streptomyces* as Symbionts: An Emerging and Widespread Theme?. FEMS Microbiol. Rev..

[19] Janssen P.H. (2006). Identifying the Dominant Soil Bacterial Taxa in Libraries of 16S rRNA and 16S rRNA Genes. Appl. Environ. Microbiol..

[20] Viaene T., Langendries S., Beirinckx S., Maes M., Goormachtig S. (2016). *Streptomyces* as a Plant’s Best Friend?. FEMS Microbiol. Ecol..

[21] Li J., Guo-zhen Z., Huang H., Qin S., Zhu W.-Y., Zhao L.-X., Xu L.-H., Zhang S., Li W.-J., Strobel G. (2012). Isolation and Characterization of Culturable Endophytic Actinobacteria Associated with *Artemisia annua* L. Antonie Van Leeuwenhoek.

[22] Álvarez-Pérez J.M., González-García S., Cobos R., Olego M.Á., Ibañez A., Díez-Galán A., Garzón-Jimeno E., Coque J.J.R. (2017). Use of Endophytic and Rhizosphere Actinobacteria from Grapevine Plants to Reduce Nursery Fungal Graft Infections That Lead to Young Grapevine Decline. Appl. Environ. Microbiol..

[23] Ayswaria R., Vasu V., Krishna R. (2020). Diverse Endophytic *Streptomyces* Species with Dynamic Metabolites and Their Meritorious Applications: A Critical Review. Crit. Rev. Microbiol..

[24] Polpass J., Maharshi A., Jha B. (2021). Actinobacteria in Natural Products Research: Progress and Prospects. Microbiol. Res..

[25] Van Der Heul H.U., Bilyk B.L., McDowall K.J., Seipke R.F., Van Wezel G.P. (2018). Regulation of Antibiotic Production in Actinobacteria: New Perspectives from the Post-Genomic Era. Nat. Prod. Rep..

[26] Chater K.F., Biró S., Lee K.J., Palmer T., Schrempf H. (2010). The Complex Extracellular Biology of *Streptomyces*. FEMS Microbiol. Rev..

[27] Cuervo L., Álvarez-García S., Salas J.A., Méndez C., Olano C., Malmierca M.G. (2023). The Volatile Organic Compounds of *Streptomyces* Spp.: An In-Depth Analysis of Their Antifungal Properties. Microorganisms.

[28] Zhao Q., Bertolli S., Park Y.J., Tan Y., Cutler K.J., Srinivas P., Asfahl K.L., Fonesca-García C., Gallagher L.A., Li Y. (2024). *Streptomyces* Umbrella Toxin Particles Block Hyphal Growth of Competing Species. Nature.

[29] Shirling E.B., Gottlieb D. (1966). Methods for the Characterization of *Streptomyces* Species. Int. J. Syst. Bacteriol..

[30] Martin J.F., McDaniel L.E. (1976). Biosynthesis of Candicidin by Phosphate-Limited Resting Cells of *Streptomyces griseus*. Eur. J. Appl. Microbiol..

[31] Kieser T., Bibb M., Buttner M., Chater K., Hopwood D. (2000). Practical Streptomyces Genetics.

[32] Collado-Romero M., Mercado-Blanco J., Olivares-García C., Valverde-Corredor A., Jiménez-Díaz R.M. (2006). Molecular Variability within and among *Verticillium dahliae* Vegetative Compatibility Groups Determined by Fluorescent Amplified Fragment Length Polymorphism and Polymerase Chain Reaction Markers. Phytopathology.

[33] Schrey S.D., Erkenbrack E., Früh E., Fengler S., Hommel K., Horlacher N., Schulz D., Ecke M., Kulik A., Fiedler H.P. (2012). Production of Fungal and Bacterial Growth Modulating Secondary Metabolites Is Widespread among Mycorrhiza-Associated Streptomycetes. BMC Microbiol..

[34] Hopwood D.A., Bibb M.J., Chater K.F., Kieser H.M., Lydiate D.J., Smith C.P., Ward J.M., Schrempf H. (1985). Genetic Manipulation of Streptomyces: A Laboratory Manual.

[35] Lane D., Stackebrandt E., Goodfellow M. (1991). 16S/23S rRNA Sequencing. Nucleic Acid Techniques in Bacterial Systematics.

[36] Kim O.S., Cho Y.J., Lee K., Yoon S.H., Kim M., Na H., Park S.C., Jeon Y.S., Lee J.H., Yi H. (2012). Introducing EzTaxon-e: A Prokaryotic 16S RRNA Gene Sequence Database with Phylotypes That Represent Uncultured Species. Int. J. Syst. Evol. Microbiol..

[37] Kimura M. (1980). A Simple Method for Estimating Evolutionary Rates of Base Substitutions through Comparative Studies of Nucleotide Sequences. J. Mol. Evol..

[38] Rong X., Huang Y. (2012). Taxonomic Evaluation of the *Streptomyces hygroscopicus* Clade Using Multilocus Sequence Analysis and DNA-DNA Hybridization, Validating the MLSA Scheme for Systematics of the Whole Genus. Syst. Appl. Microbiol..

[39] Guo Y.P., Zheng W., Rong X.Y., Huang Y. (2008). A Multilocus Phylogeny of the *Streptomyces griseus* 16S rRNA Gene Clade: Use of Multilocus Sequence Analysis for Streptomycete Systematics. Int. J. Syst. Evol. Microbiol..

[40] Rong X., Guo Y., Huang Y. (2009). Proposal to Reclassify the *Streptomyces albidoflavus* Clade on the Basis of Multilocus Sequence Analysis and DNA-DNA Hybridization, and Taxonomic Elucidation of *Streptomyces griseus* subsp. solvifaciens. Syst. Appl. Microbiol..

[41] Koren S., Walenz B.P., Berlin K., Miller J.R., Bergman N.H., Phillippy A.M. (2017). Canu: Scalable and Accurate Long-Read Assembly via Adaptive k-Mer Weighting and Repeat Separation. Genome Res..

[42] Mikheenko A., Prjibelski A., Saveliev V., Antipov D., Gurevich A. (2018). Versatile Genome Assembly Evaluation with QUAST-LG. Bioinformatics.

[43] Overbeek R., Olson R., Pusch G.D., Olsen G.J., Davis J.J., Disz T., Edwards R.A., Gerdes S., Parrello B., Shukla M. (2014). The SEED and the Rapid Annotation of Microbial Genomes Using Subsystems Technology (RAST). Nucleic Acids Res..

[44] Schleifer K.H., Kandler O. (1972). Peptidoglycan Types of Bacterial Cell Walls and Their Taxonomic Implications. Bacteriol. Rev..

[45] Lechevalier M.P., Lechevalier H.A. (1970). Chemical Composition as a Criterion in the Classification of Aerobic Actinomycetes. Int. J. Syst. Bacteriol..

[46] Minnikin A’ D.E., O’donnell A.G., Goodfellow M., Alderson G., Athalye M., Schaal A., Parlett J.H. (1984). An Integrated Procedure for the Extraction of Bacterial Isoprenoid Quinones and Polar Lipids. J. Microbiol. Methods.

[47] Kroppenstedt R.M., Goodfellow M., Dworkin M., Falkow S., Rosenberg E., Schleifer H.H., Stackebrandt E. (2006). The Family Thermomonosporaceae: Actinocorallia, Actinomadura, Spirillispora and Thermomonospora. The Prokaryotes: A Handbook on the Biology of Bacteria. Archaea, Bacteria, Firmicutes, Actinomycetes.

[48] Sasser M. (1990). MIDI Technical Note 101. Identification of Bacteria by Gas Chromatography of Cellular Fatty Acids. MIDI Technical Note 101.

[49] Vieira S., Huber K.J., Neumann-Schaal M., Geppert A., Luckner M., Wanner G., Overmann J. (2021). *Usitatibacter rugosus* Gen. Nov., Sp. Nov. and *Usitatibacter palustris* Sp. Nov., Novel Members of *Usitatibacteraceae* Fam. Nov. within the Order *Nitrosomonadales* Isolated from Soil. Int. J. Syst. Evol. Microbiol..

[50] Moss C.W., Lambert-Fair M.A. (1989). Location of Double Bonds in Monounsaturated Fatty Acids of *Campylobacter cryaerophila* with Dimethyl Disulfide Derivatives and Combined Gas Chromatography-Mass Spectrometry. J. Clin. Microbiol..

[51] Schumann P., Kalensee F., Cao J., Criscuolo A., Clermont D., Köhler J.M., Meier-Kolthoff J.P., Neumann-Schaal M., Tindall B.J., Pukall R. (2021). Reclassification of *Haloactinobacterium glacieicola* as *Occultella glacieicola* Gen. Nov., Comb. Nov., of *Haloactinobacterium album* as *Ruania alba* Comb. Nov, with an Emended Description of the Genus *Ruania*, Recognition That the Genus Names *Haloactinobacterium* and *Ruania* Are Heterotypic Synonyms and Description of *Occultella aeris* Sp. Nov., a Halotolerant Isolate from Surface Soil Sampled at an Ancient Copper Smelter. Int. J. Syst. Evol. Microbiol..

[52] Meier-Kolthoff J.P., Auch A.F., Klenk H.-P., Göker M.G. (2013). Genome Sequence-Based Species Delimitation with Confidence Intervals and Improved Distance Functions. BMC Bioinform..

[53] Meier-Kolthoff J.P., Carbasse J.S., Peinado-Olarte R.L., Göker M. (2022). TYGS and LPSN: A Database Tandem for Fast and Reliable Genome-Based Classification and Nomenclature of Prokaryotes. Nucleic Acids Res..

[54] Yoon S.H., Ha S.M., Kwon S., Lim J., Kim Y., Seo H., Chun J. (2017). Introducing EzBioCloud: A Taxonomically United Database of 16S rRNA Gene Sequences and Whole-Genome Assemblies. Int. J. Syst. Evol. Microbiol..

[55] Meier-Kolthoff J.P., Göker M. (2019). TYGS Is an Automated High-Throughput Platform for State-of-the-Art Genome-Based Taxonomy. Nat. Commun..

[56] Meier-Kolthoff J.P., Göker M., Spröer C., Klenk H.P. (2013). When Should a DDH Experiment Be Mandatory in Microbial Taxonomy?. Arch. Microbiol..

[57] Blin K., Shaw S., Augustijn H.E., Reitz Z.L., Biermann F., Alanjary M., Fetter A., Terlouw B.R., Metcalf W.W., Helfrich E.J.N. (2023). AntiSMASH 7.0: New and Improved Predictions for Detection, Regulation, Chemical Structures and Visualisation. Nucleic Acids Res..

[58] Medema M.H., Kottmann R., Yilmaz P., Cummings M., Biggins J.B., Blin K., De Bruijn I., Chooi Y.H., Claesen J., Coates R.C. (2015). Minimum Information about a Biosynthetic Gene Cluster. Nat. Chem. Biol..

[59] van den Belt M., Gilchrist C., Booth T.J., Chooi Y.H., Medema M.H., Alanjary M. (2023). CAGECAT: The CompArative GEne Cluster Analysis Toolbox for Rapid Search and Visualisation of Homologous Gene Clusters. BMC Bioinform..

[60] Navarro-Muñoz J.C., Selem-Mojica N., Mullowney M.W., Kautsar S.A., Tryon J.H., Parkinson E.I., De Los Santos E.L.C., Yeong M., Cruz-Morales P., Abubucker S. (2020). A Computational Framework to Explore Large-Scale Biosynthetic Diversity. Nat. Chem. Biol..

[61] Das R., Romi W., Das R., Sharma H.K., Thakur D. (2018). Antimicrobial Potentiality of Actinobacteria Isolated from Two Microbiologically Unexplored Forest Ecosystems of Northeast India. BMC Microbiol..

[62] Awla H.K., Rashid T.S. (2020). HPLC Fractionation: A Comparative Analysis of Anti-Fungal Compounds from Different *Streptomyces* Isolates Inhibiting *Colletotrichum acutatum*. Biocatal. Agric. Biotechnol..

[63] López-Moral A., Agustí-Brisach C., Leiva-Egea F.M., Trapero A. (2022). Influence of Cultivar and Biocontrol Treatments on the Effect of Olive Stem Extracts on the Viability of *Verticillium dahliae* Conidia. Plants.

[64] EUCAST Definitive Document (1998). Methods for the determination of susceptibility of bacteria to antimicrobial agents. Terminology. Clin. Microbiol. Infect..

[65] Kim M., Oh H.S., Park S.C., Chun J. (2014). Towards a Taxonomic Coherence between Average Nucleotide Identity and 16S rRNA Gene Sequence Similarity for Species Demarcation of Prokaryotes. Int. J. Syst. Evol. Microbiol..

[66] Kämpfer P. (2012). Genus Streptomyces Waksman and Henrici 1943, 339AL Emend. Witt and Stackebrandt 1990, 370, Emend. Wellington, Stackebrandt, Sanders, Wolstrup and Jorgensen, 1992, 159. Bergey’s Manual of Systematic Bacteriology.

[67] Wayne L.G., Brenner D.J., Colwell R.R., Grimont P.A.D., Kandler O., Krichevsky M.I., Moore L.H., Moore W.E.C., Murray R.G.E., Stackebrandt E. (1987). Report of the Ad Hoc Committee on Reconciliation of Approaches to Bacterial Systematics. Int. J. Syst. Bacteriol..

[68] Sun F., Xu S., Jiang F., Liu W. (2018). Genomic-Driven Discovery of an Amidinohydrolase Involved in the Biosynthesis of Mediomycin A. Appl. Microbiol. Biotechnol..

[69] Cheng J., Yang S.H., Palaniyandi S.A., Han J.S., Yoon T.M., Kim T.J., Suh J.W. (2010). Azalomycin F Complex Is an Antifungal Substance Produced by *Streptomyces malaysiensis* MJM1968 Isolated from Agricultural Soil. J. Appl. Biol. Chem..

[70] Bastidas R.J., Shertz C.A., Lee S.C., Heitman J., Cardenas M.E. (2012). Rapamycin Exerts Antifungal Activity In Vitro and In Vivo against *Mucor circinelloides* via FKBP12-Dependent Inhibition of Tor. Eukaryot. Cell.

[71] Jiang H., Rao Y., Mei L., Wang Y. (2021). Antifungal Activity of Rapamycin on *Botryosphaeria dothidea* and Its Effect against Chinese Hickory Canker. Pest. Manag. Sci..

[72] Li L., Zhu T., Song Y., Luo X., Feng L., Zhuo F., Li F., Ren M. (2019). Functional Characterization of Target of Rapamycin Signaling in *Verticillium dahliae*. Front. Microbiol..

[73] Netzker T., Schroeckh V., Gregory M.A., Flak M., Krespach M.K.C., Leadlay P.F., Brakhage A.A. (2016). An Efficient Method to Generate Gene Deletion Mutants of the Rapamycin-Producing Bacterium *Streptomyces iranensis* HM 35. Appl. Environ. Microbiol..

[74] Fang A., Wong G.K., Demain A.L. (2000). Enhancement of the Antifungal Activity of Rapamycin by the Coproduced Elaiophylin and Nigerin. J. Antibiot..

[75] Mandala S.M., Thornton R.A., Milligan J., Rosenbach M., Garcia-Calvo M., Bull H.G., Harris G., Abruzzo G.K., Flattery A.M., Gill C.J. (1998). Rustmicin, a Potent Antifungal Agent, Inhibits Sphingolipid Synthesis at Inositol Phosphoceramide Synthase. J. Biol. Chem..

[76] Fujita K., Sugiyama R., Nishimura S., Ishikawa N., Arai M.A., Ishibashi M., Kakeya H. (2016). Stereochemical Assignment and Biological Evaluation of BE-14106 Unveils the Importance of One Acetate Unit for the Antifungal Activity of Polyene Macrolactams. J. Nat. Prod..

[77] Goh F., Zhang M.M., Lim T.R., Low K.N., Nge C.E., Heng E., Yeo W.L., Sirota F.L., Crasta S., Tan Z. (2020). Identification and Engineering of 32 Membered Antifungal Macrolactone Notonesomycins. Microb. Cell Fact..

[78] Shentu X.P., Cao Z.Y., Xiao Y., Tang G., Ochi K., Yu X.P. (2018). Substantial Improvement of Toyocamycin Production in *Streptomyces diastatochromogenes* by Cumulative Drug-Resistance Mutations. PLoS ONE.

[79] Jo H.G., Adidjaja J.J., Kim D.K., Park B.S., Lee N., Cho B.K., Kim H.U., Oh M.K. (2022). Comparative Genomic Analysis of *Streptomyces rapamycinicus* NRRL 5491 and Its Mutant Overproducing Rapamycin. Sci. Rep..

[80] Park S.R., Yoo Y.J., Ban Y.H., Yoon Y.J. (2010). Biosynthesis of Rapamycin and Its Regulation: Past Achievements and Recent Progress. J. Antibiot..

[81] Yoo Y.J., Hwang J.Y., Shin H.L., Cui H., Lee J., Yoon Y.J. (2015). Characterization of Negative Regulatory Genes for the Biosynthesis of Rapamycin in *Streptomyces rapamycinicus* and Its Application for Improved Production. J. Ind. Microbiol. Biotechnol..

[82] Cuthbertson L., Nodwell J.R. (2013). The TetR Family of Regulators. Microbiol. Mol. Biol. Rev..

[83] Molnár I., Aparicio J.F., Haydock S.F., Khaw L., Schwecke T., König A., Staunton J., Leadlay P.F. (1996). Organisation of the Biosynthetic Gene Cluster for Rapamycin in *Streptomyces hygroscopicus*: Analysis of Genes Flanking the Polyketide Synthase. Gene.

[84] Mrak P., Krastel P., Lukančič P.P., Tao J., Pistorius D., Moore C.M. (2018). Discovery of the Actinoplanic Acid Pathway in *Streptomyces rapamycinicus* Reveals a Genetically Conserved Synergism with Rapamycin. J. Biol. Chem..

[85] McAlpine J.B., Swanson S.J., Jackson M., Whittern D.N. (1991). Revised NMR Assignments for Rapamycin. J. Antibiot..

[86] Kang S., Han J., Jang S.C., An J.S., Kang I., Kwon Y., Nam S.J., Shim S.H., Cho J.C., Lee S.K. (2022). Epoxinnamide: An Epoxy Cinnamoyl-Containing Nonribosomal Peptide from an Intertidal Mudflat-Derived *Streptomyces* sp. *Mar*. Drugs.

[87] Hamedi J., Mohammadipanah F., Klenk H.P., Pötter G., Schumann P., Spröer C., Von Jan M., Kroppenstedt R.M. (2010). *Streptomyces iranensis* Sp. Nov., Isolated from Soil. Int. J. Syst. Evol. Microbiol..

[88] Gallego-Clemente E., Moreno-González V., Ibáñez A., Calvo-Peña C., Ghoreshizadeh S., Radišek S., Cobos R., Coque J.J.R. (2023). Changes in the Microbial Composition of the Rhizosphere of Hop Plants Affected by Verticillium Wilt Caused by *Verticillium Nonalfalfae*. Microorganisms.

[89] Vézina C., Kudelski A., Sehgal S.N. (1975). Rapamycin (AY-22,989), a New Antifungal Antibiotic. J. Antibiot..

[90] Nishida H., Sakakibara T., Aoki F., Saito T., Ichikawa K., Inagaki T., Kojima Y., Yamauchi Y., Huangt L.H., Guadliana M.A. (1995). Generation of Novel Rapamycin Structures by Microbial Manipulations. J. Antibiot..

[91] Trevillian P. (2006). Immunosuppressants-Clinical Applications. Aust. Prescr..

[92] Martel R.R., Klicius J., Galet S. (1977). Inhibition of the Immune Response by Aapamycin, a New Antifungal Antibiotic. Can. J. Physiol. Pharmacol..

[93] Law B.K. (2005). Rapamycin: An Anti-Cancer Immunosuppressant?. Crit. Rev. Oncol. Hematol..

[94] Tain L.S., Mortiboys H., Tao R.N., Ziviani E., Bandmann O., Whitworth A.J. (2009). Rapamycin Activation of 4E-BP Prevents Parkinsonian Dopaminergic Neuron Loss. Nat. Neurosci..

[95] Malagelada C., Jin Z.H., Jackson-Lewis V., Przedborski S., Greene L.A. (2010). Rapamycin Protects against Neuron Death in In Vitro and In Vivo Models of Parkinson’s Disease. J. Neurosci..

[96] Sharp Z.D., Strong R. (2023). Rapamycin, the Only Drug That Has Been Consistently Demonstrated to Increase Mammalian Longevity. An Update. Exp. Gerontol..

[97] Yoo Y.J., Kim H., Park S.R., Yoon Y.J. (2017). An Overview of Rapamycin: From Discovery to Future Perspectives. J. Ind. Microbiol. Biotechnol..

[98] Kuščer E., Coates N., Challis I., Gregory M., Wilkinson B., Sheridan R., Petković H. (2007). Roles of *RapH* and *RapG* in Positive Regulation of Rapamycin Biosynthesis in *Streptomyces hygroscopicus*. J. Bacteriol..

[99] Schwecke T., Aparicio J.F., Molnar I., König A., Khaw L.E., Haydock S.F., Oliynyk M., Caffrey P., Cortés J., Lester J.B. (1995). The Biosynthetic Gene Cluster for the Polyketide Immunosuppressant Rapamycin. Proc. Natl. Acad. Sci. USA.

[100] Gatto G.J., Boyne M.T., Kelleher N.L., Walsh C.T. (2006). Biosynthesis of Pipecolic Acid by *RapL*, a Lysine Cyclodeaminase Encoded in the Rapamycin Gene Cluster. J. Am. Chem. Soc..

[101] Kobylarz M.J., Grigg J.C., Takayama S.I.J., Rai D.K., Heinrichs D.E., Murphy M.E.P. (2014). Synthesis of L-2,3-Diaminopropionic Acid, a Siderophore and Antibiotic Precursor. Chem. Biol..

[102] König A., Schwecke T., Molnár I., Böhm G.A., Lowden P.A.S., Staunton J., Leadlay P.F. (1997). The Pipecolate-Incorporating Enzyme for the Biosynthesis of the Immunosuppressant Rapamycin—Nucleotide Sequence Analysis, Disruption and Heterologous Expression of Rap P from Streptomyces Hygroscopicus. Eur. J. Biochem..

