# Supplementary material for: Rapamycin Plays a Pivotal Role in the Potent Antifungal Activity Exhibited Against Verticillium dahliae by Streptomyces iranensis OE54 and Streptomyces lacaronensis sp. nov. Isolated from Olive Roots
Source: Microorganisms. 2025 Jul 9;13(7):1622. doi: 10.3390/microorganisms13071622 (PMC12298158; doi:10.3390/microorganisms13071622)
Supplement: Supplementary file 1 [file microorganisms-13-01622-s001.zip › Supplementary Figure S1.pdf]

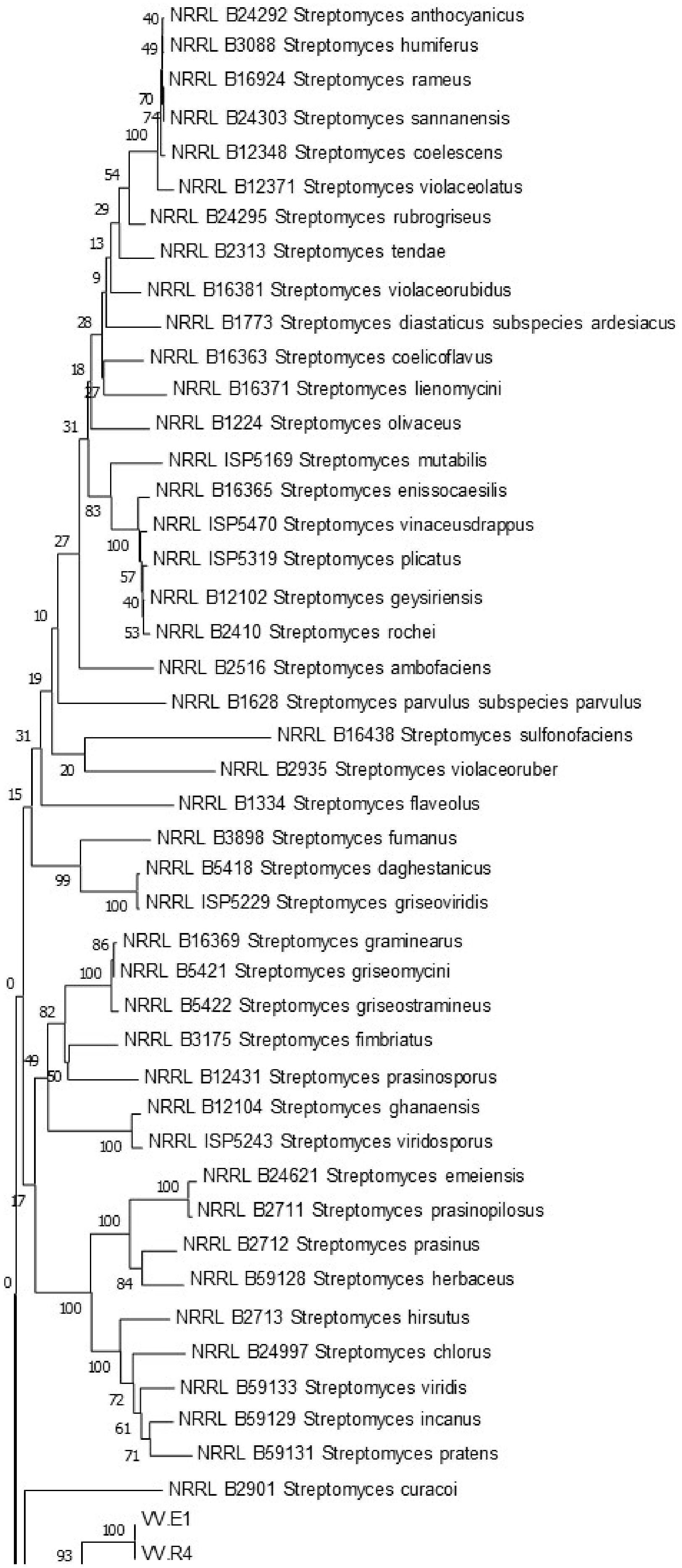

NRRL B24292 Streptomyces anthocyanicus
NRRL B3088 Streptomyces humiferus
NRRL B16924 Streptomyces rameus
NRRL B24303 Streptomyces sannanensis
NRRL B12348 Streptomyces coelescens
NRRL B12371 Streptomyces violaceolatus
NRRL B24295 Streptomyces rubrogriseus
NRRL B2313 Streptomyces tendae
NRRL B16381 Streptomyces violaceorubidus
NRRL B1773 Streptomyces diastaticus subspecies ardesiacus
NRRL B16363 Streptomyces coelicoflavus
NRRL B16371 Streptomyces lienomycini
NRRL B1224 Streptomyces olivaceus
NRRL ISP5169 Streptomyces mutabilis
NRRL B16365 Streptomyces enissocaesilis
NRRL ISP5470 Streptomyces vinaceusdrappus
NRRL ISP5319 Streptomyces plicatus
NRRL B12102 Streptomyces geysiriensis
NRRL B2410 Streptomyces rochei
NRRL B2516 Streptomyces ambofaciens
NRRL B1628 Streptomyces parvulus subspecies parvulus
NRRL B16438 Streptomyces sulfonofaciens
NRRL B2935 Streptomyces violaceoruber
NRRL B1334 Streptomyces flaveolus
NRRL B3898 Streptomyces fumanus
NRRL B5418 Streptomyces daghestanicus
NRRL ISP5229 Streptomyces griseoviridis
NRRL B16369 Streptomyces graminearus
NRRL B5421 Streptomyces griseomycini
NRRL B5422 Streptomyces griseostramineus
NRRL B3175 Streptomyces fimbriatus
NRRL B12431 Streptomyces prasinosporus
NRRL B12104 Streptomyces ghanaensis
NRRL ISP5243 Streptomyces viridosporus
NRRL B24621 Streptomyces emeiensis
NRRL B2711 Streptomyces prasinopilosus
NRRL B2712 Streptomyces prasinus
NRRL B59128 Streptomyces herbaceus
NRRL B2713 Streptomyces hirsutus
NRRL B24997 Streptomyces chlorus
NRRL B59133 Streptomyces viridis
NRRL B59129 Streptomyces incanus
NRRL B59131 Streptomyces pratens
NRRL B2901 Streptomyces curacoi
VV.E1
VV.R4

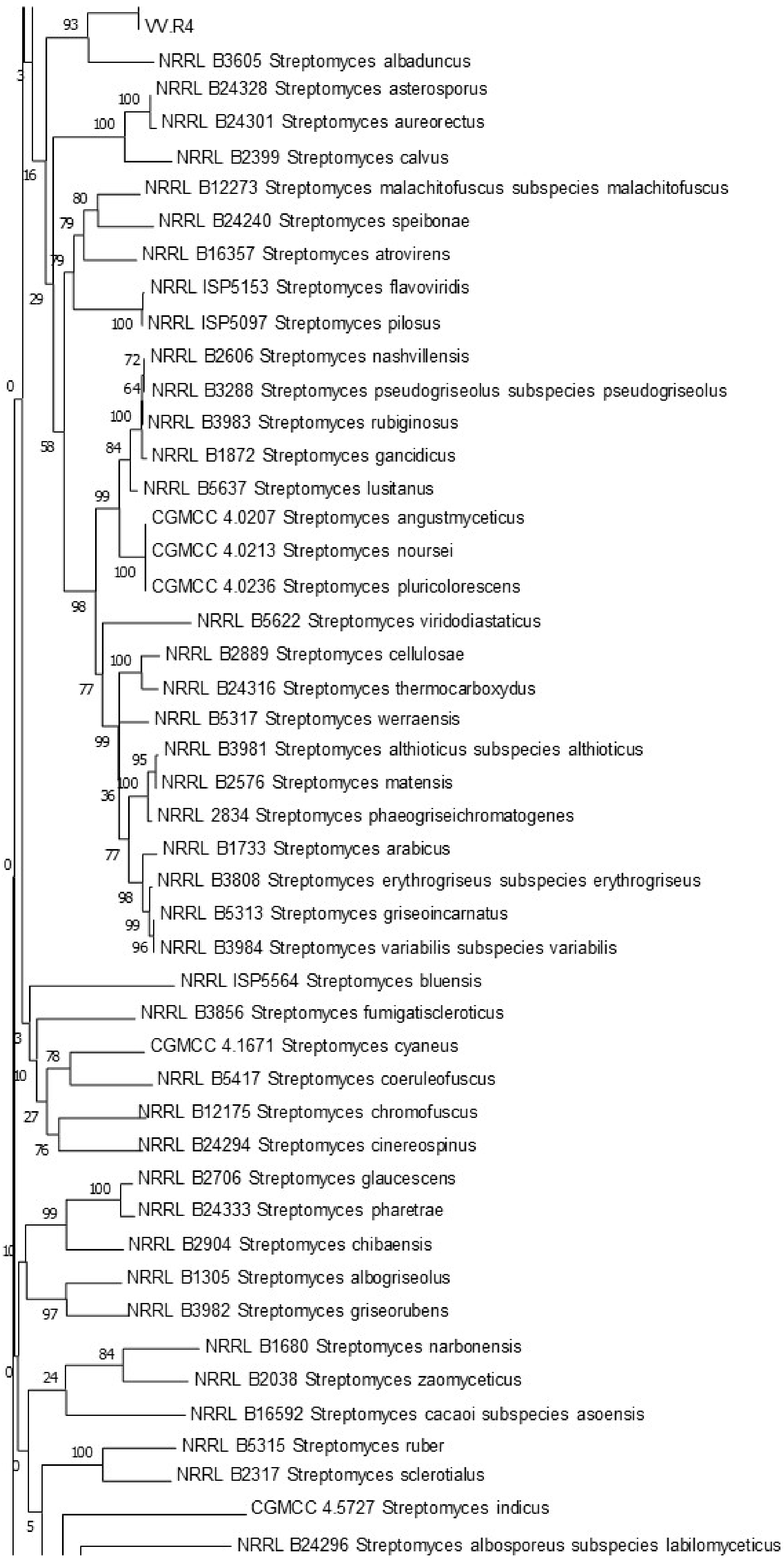
VV.R4
NRRL B3605 Streptomyces albaduncus
NRRL B24328 Streptomyces asterosporus
NRRL B24301 Streptomyces aureorectus
NRRL B2399 Streptomyces calvus
NRRL B12273 Streptomyces malachitofuscus subspecies malachitofuscus
NRRL B24240 Streptomyces speibonae
NRRL B16357 Streptomyces atrovirens
NRRL ISP5153 Streptomyces flavoviridis
NRRL ISP5097 Streptomyces pilosus
NRRL B2606 Streptomyces nashvillensis
NRRL B3288 Streptomyces pseudogriseolus subspecies pseudogriseolus
NRRL B3983 Streptomyces rubiginosus
NRRL B1872 Streptomyces gancidicus
NRRL B5637 Streptomyces lusitanus
CGMCC 4.0207 Streptomyces angustmyceticus
CGMCC 4.0213 Streptomyces noursei
CGMCC 4.0236 Streptomyces pluricolorescens
NRRL B5622 Streptomyces viridodiastaticus
NRRL B2889 Streptomyces cellulosae
NRRL B24316 Streptomyces thermocarboxydus
NRRL B5317 Streptomyces werraensis
NRRL B3981 Streptomyces althioticus subspecies althioticus
NRRL B2576 Streptomyces matensis
NRRL 2834 Streptomyces phaeogriseichromatogenes
NRRL B1733 Streptomyces arabicus
NRRL B3808 Streptomyces erythrogriseus subspecies erythrogriseus
NRRL B5313 Streptomyces griseoincarnatus
NRRL B3984 Streptomyces variabilis subspecies variabilis
NRRL ISP5564 Streptomyces bluensis
NRRL B3856 Streptomyces fumigatiscleroticus
CGMCC 4.1671 Streptomyces cyaneus
NRRL B5417 Streptomyces coeruleofuscus
NRRL B12175 Streptomyces chromofuscus
NRRL B24294 Streptomyces cinereospinus
NRRL B2706 Streptomyces glaucescens
NRRL B24333 Streptomyces pharetrae
NRRL B2904 Streptomyces chibaensis
NRRL B1305 Streptomyces albogriseolus
NRRL B3982 Streptomyces griseorubens
NRRL B1680 Streptomyces narbonensis
NRRL B2038 Streptomyces zaomyceticus
NRRL B16592 Streptomyces cacaoi subspecies asoensis
NRRL B5315 Streptomyces ruber
NRRL B2317 Streptomyces sclerotialus
CGMCC 4.5727 Streptomyces indicus
NRRL B24296 Streptomyces albosporeus subspecies labilomyceticus

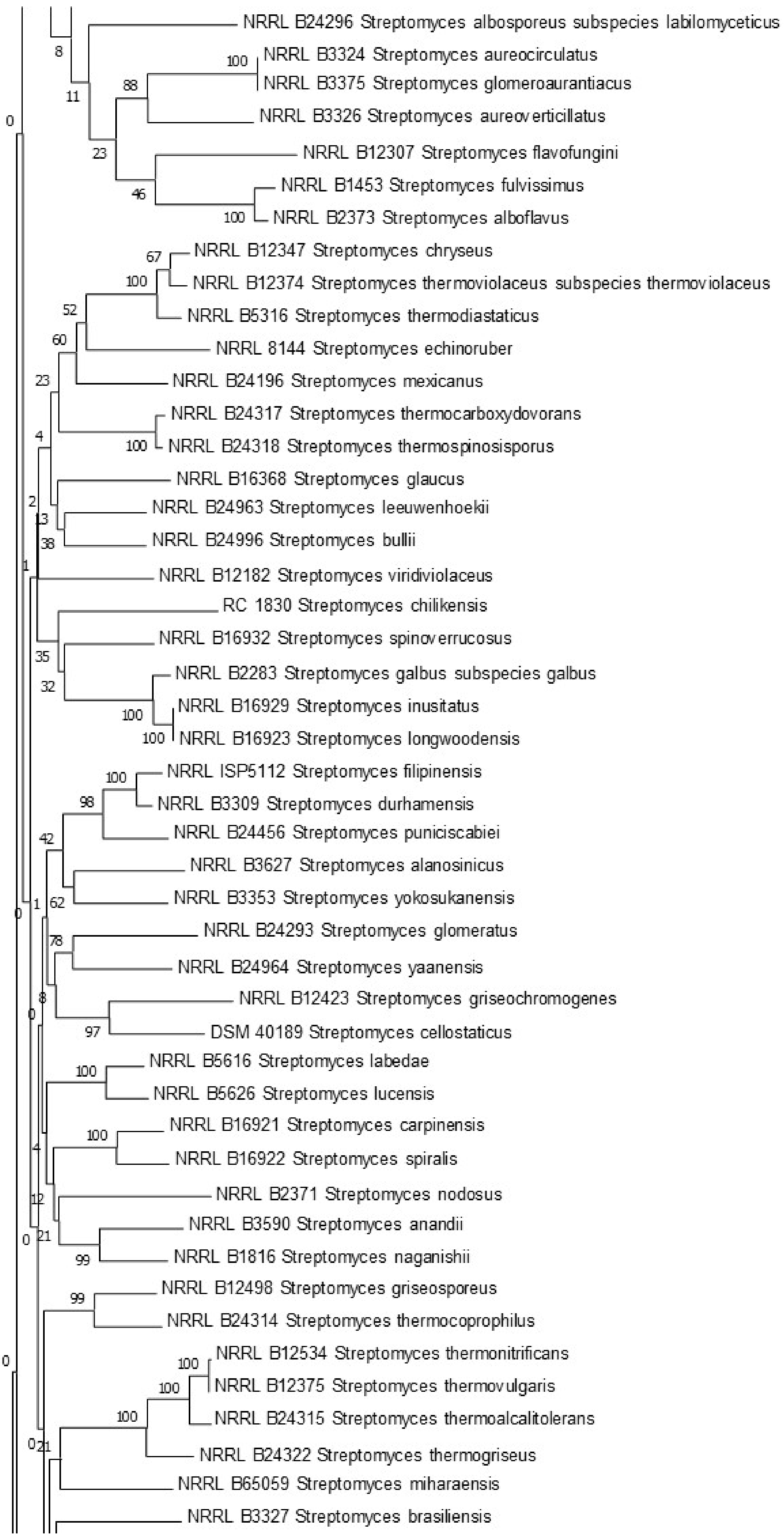
NRRL B24296 Streptomyces albosporeus subspecies labilomyceticus
NRRL B3324 Streptomyces aureocirculatus
NRRL B3375 Streptomyces glomeroaurantiacus
NRRL B3326 Streptomyces aureoverticillatus
NRRL B12307 Streptomyces flavofungini
NRRL B1453 Streptomyces fulvissimus
NRRL B2373 Streptomyces alboflavus
NRRL B12347 Streptomyces chryseus
NRRL B12374 Streptomyces thermoviolaceus subspecies thermoviolaceus
NRRL B5316 Streptomyces thermodiastaticus
NRRL 8144 Streptomyces echinoruber
NRRL B24196 Streptomyces mexicanus
NRRL B24317 Streptomyces thermocarboxydovorans
NRRL B24318 Streptomyces thermospinosisporus
NRRL B16368 Streptomyces glaucus
NRRL B24963 Streptomyces leeuwenhoekii
NRRL B24996 Streptomyces bullii
NRRL B12182 Streptomyces viridiviolaceus
RC 1830 Streptomyces chilikensis
NRRL B16932 Streptomyces spinoverrucosus
NRRL B2283 Streptomyces galbus subspecies galbus
NRRL B16929 Streptomyces inusitatus
NRRL B16923 Streptomyces longwoodensis
NRRL ISP5112 Streptomyces filipinensis
NRRL B3309 Streptomyces durhamensis
NRRL B24456 Streptomyces puniciscabiei
NRRL B3627 Streptomyces alanosinicus
NRRL B3353 Streptomyces yokosukanensis
NRRL B24293 Streptomyces glomeratus
NRRL B24964 Streptomyces yaanensis
NRRL B12423 Streptomyces griseochromogenes
DSM 40189 Streptomyces cellostaticus
NRRL B5616 Streptomyces labedae
NRRL B5626 Streptomyces lucensis
NRRL B16921 Streptomyces carpinensis
NRRL B16922 Streptomyces spiralis
NRRL B2371 Streptomyces nodosus
NRRL B3590 Streptomyces anandii
NRRL B1816 Streptomyces naganishii
NRRL B12498 Streptomyces griseosporeus
NRRL B24314 Streptomyces thermocoprophilus
NRRL B12534 Streptomyces thermonitrificans
NRRL B12375 Streptomyces thermovulgaris
NRRL B24315 Streptomyces thermoalcalitolerans
NRRL B24322 Streptomyces thermogriseus
NRRL B65059 Streptomyces miharaensis
NRRL B3327 Streptomyces brasiliensis

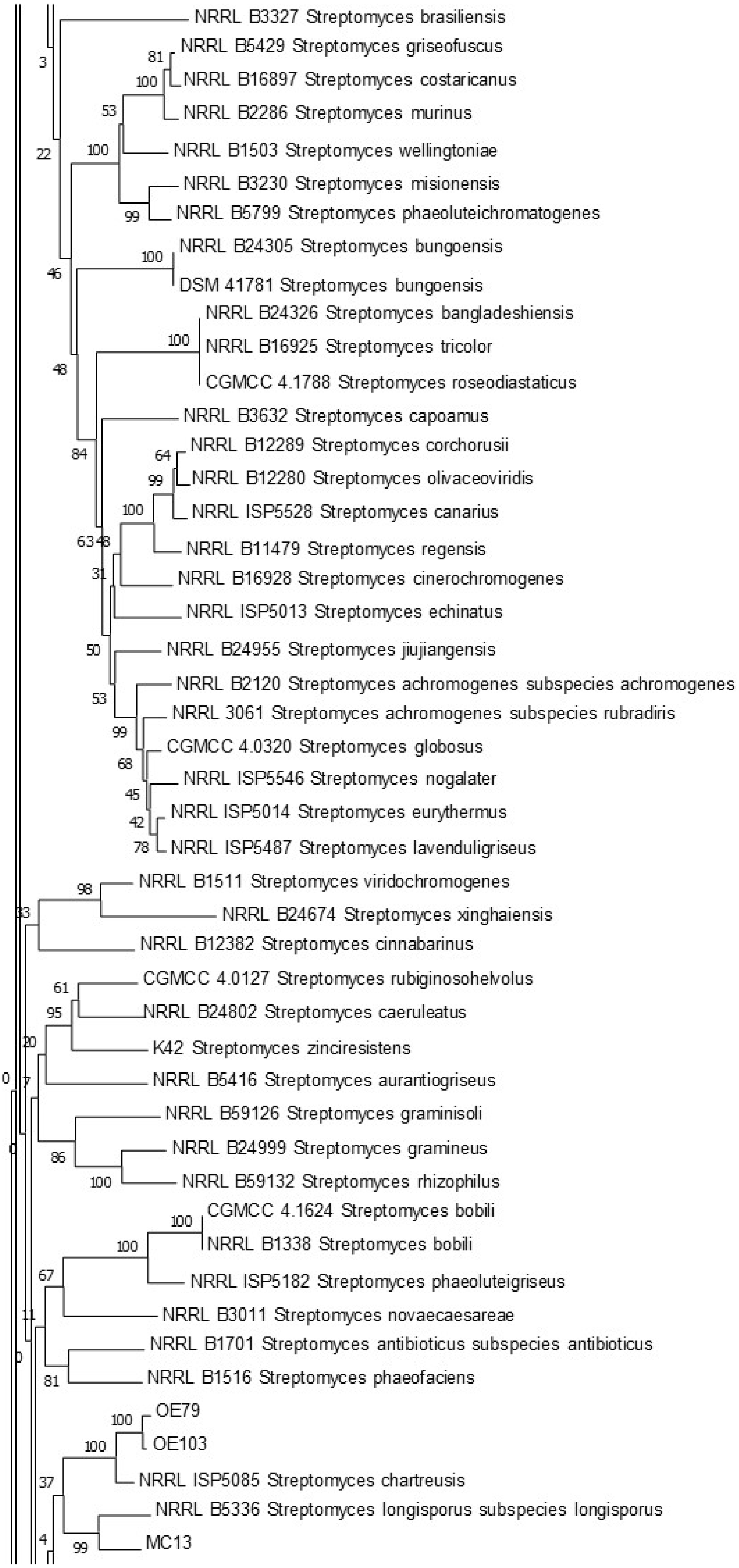
NRRL B3327 Streptomyces brasiliensis
NRRL B5429 Streptomyces griseofuscus
NRRL B16897 Streptomyces costaricanus
NRRL B2286 Streptomyces murinus
NRRL B1503 Streptomyces wellingtoniae
NRRL B3230 Streptomyces misionensis
NRRL B5799 Streptomyces phaeoluteichromatogenes
NRRL B24305 Streptomyces bungoensis
DSM 41781 Streptomyces bungoensis
NRRL B24326 Streptomyces bangladeshiensis
NRRL B16925 Streptomyces tricolor
CGMCC 4.1788 Streptomyces roseodiastaticus
NRRL B3632 Streptomyces capoamus
NRRL B12289 Streptomyces corchorusii
NRRL B12280 Streptomyces olivaceoviridis
NRRL ISP5528 Streptomyces canarius
NRRL B11479 Streptomyces regensis
NRRL B16928 Streptomyces cinerochromogenes
NRRL ISP5013 Streptomyces echinatus
NRRL B24955 Streptomyces jiujiangensis
NRRL B2120 Streptomyces achromogenes subspecies achromogenes
NRRL 3061 Streptomyces achromogenes subspecies rubradiris
CGMCC 4.0320 Streptomyces globosus
NRRL ISP5546 Streptomyces nogalater
NRRL ISP5014 Streptomyces eurythermus
NRRL ISP5487 Streptomyces lavenduligriseus
NRRL B1511 Streptomyces viridochromogenes
NRRL B24674 Streptomyces xinghaiensis
NRRL B12382 Streptomyces cinnabarinus
CGMCC 4.0127 Streptomyces rubiginosohelvolus
NRRL B24802 Streptomyces caeruleatus
K42 Streptomyces zinciresistens
NRRL B5416 Streptomyces aurantiogriseus
NRRL B59126 Streptomyces graminisoli
NRRL B24999 Streptomyces gramineus
NRRL B59132 Streptomyces rhizophilus
CGMCC 4.1624 Streptomyces bobili
NRRL B1338 Streptomyces bobili
NRRL ISP5182 Streptomyces phaeoluteigriseus
NRRL B3011 Streptomyces novaecaesareae
NRRL B1701 Streptomyces antibioticus subspecies antibioticus
NRRL B1516 Streptomyces phaeofaciens
OE79
OE103
NRRL ISP5085 Streptomyces chartreusis
NRRL B5336 Streptomyces longisporus subspecies longisporus
MC13

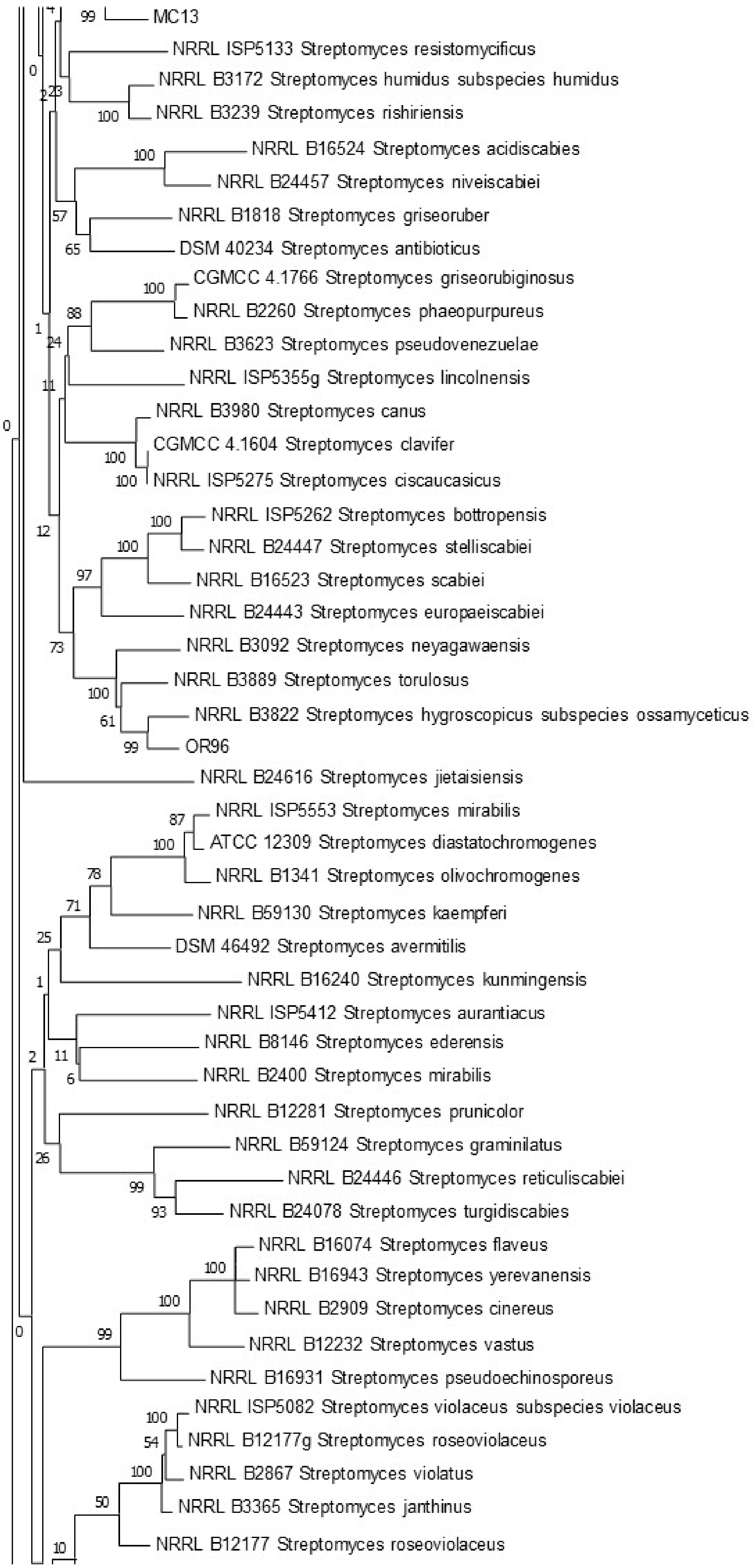
MC13
NRRL ISP5133 Streptomyces resistomycificus
NRRL B3172 Streptomyces humidus subspecies humidus
NRRL B3239 Streptomyces rishiriensis
NRRL B16524 Streptomyces acidiscabies
NRRL B24457 Streptomyces niveiscabiei
NRRL B1818 Streptomyces griseoruber
DSM 40234 Streptomyces antibioticus
CGMCC 4.1766 Streptomyces griseorubiginosus
NRRL B2260 Streptomyces phaeopurpureus
NRRL B3623 Streptomyces pseudovenezuelae
NRRL ISP5355g Streptomyces lincolnensis
NRRL B3980 Streptomyces canus
CGMCC 4.1604 Streptomyces clavifer
NRRL ISP5275 Streptomyces ciscaucasicus
NRRL ISP5262 Streptomyces bottropensis
NRRL B24447 Streptomyces stelliscabiei
NRRL B16523 Streptomyces scabiei
NRRL B24443 Streptomyces europaeiscabiei
NRRL B3092 Streptomyces neyagawaensis
NRRL B3889 Streptomyces torulosus
NRRL B3822 Streptomyces hygroscopicus subspecies ossamyceticus
OR96
NRRL B24616 Streptomyces jietaisiensis
NRRL ISP5553 Streptomyces mirabilis
ATCC 12309 Streptomyces diastatochromogenes
NRRL B1341 Streptomyces olivochromogenes
NRRL B59130 Streptomyces kaempferi
DSM 46492 Streptomyces avermitilis
NRRL B16240 Streptomyces kunmingensis
NRRL ISP5412 Streptomyces aurantiacus
NRRL B8146 Streptomyces ederensis
NRRL B2400 Streptomyces mirabilis
NRRL B12281 Streptomyces prunicolor
NRRL B59124 Streptomyces graminilatus
NRRL B24446 Streptomyces reticuliscabiei
NRRL B24078 Streptomyces turgidiscabies
NRRL B16074 Streptomyces flaveus
NRRL B16943 Streptomyces yerevanensis
NRRL B2909 Streptomyces cinereus
NRRL B12232 Streptomyces vastus
NRRL B16931 Streptomyces pseudoechinosporeus
NRRL ISP5082 Streptomyces violaceus subspecies violaceus
NRRL B12177g Streptomyces roseoviolaceus
NRRL B2867 Streptomyces violatus
NRRL B3365 Streptomyces janthinus
NRRL B12177 Streptomyces roseoviolaceus

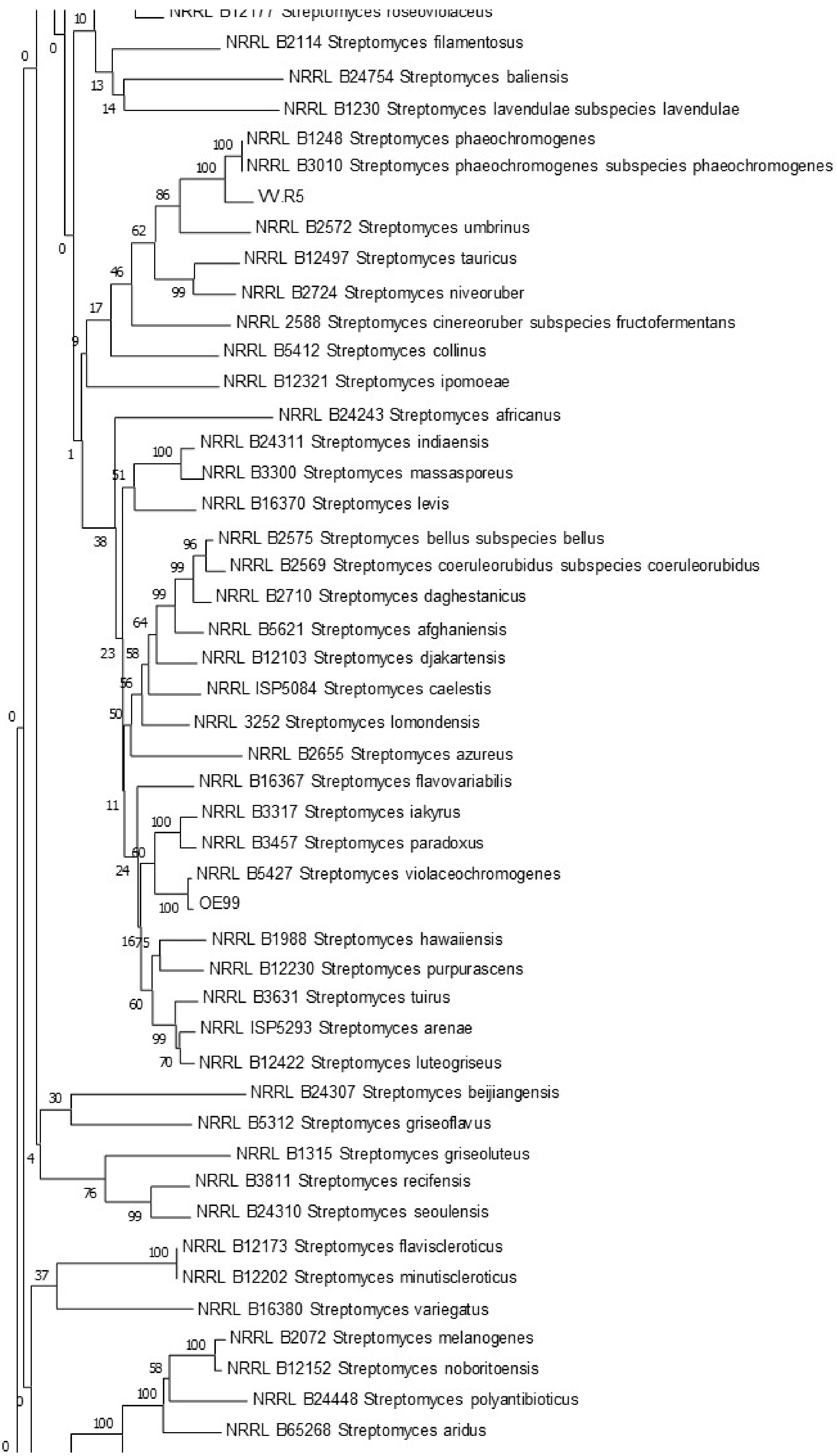

NRRL B2114 Streptomyces filamentosus
NRRL B24754 Streptomyces baliensis
NRRL B1230 Streptomyces lavendulae subspecies lavendulae
NRRL B1248 Streptomyces phaeochromogenes
NRRL B3010 Streptomyces phaeochromogenes subspecies phaeochromogenes
VV.R5
NRRL B2572 Streptomyces umbrinus
NRRL B12497 Streptomyces tauricus
NRRL B2724 Streptomyces niveoruber
NRRL 2588 Streptomyces cinereoruber subspecies fructofermentans
NRRL B5412 Streptomyces collinus
NRRL B12321 Streptomyces ipomoeae
NRRL B24243 Streptomyces africanus
NRRL B24311 Streptomyces indiaensis
NRRL B3300 Streptomyces massasporeus
NRRL B16370 Streptomyces levis
NRRL B2575 Streptomyces bellus subspecies bellus
NRRL B2569 Streptomyces coeruleorubidus subspecies coeruleorubidus
NRRL B2710 Streptomyces daghestanicus
NRRL B5621 Streptomyces afghaniensis
NRRL B12103 Streptomyces djakartensis
NRRL ISP5084 Streptomyces caelestis
NRRL 3252 Streptomyces lomondensis
NRRL B2655 Streptomyces azureus
NRRL B16367 Streptomyces flavovariabilis
NRRL B3317 Streptomyces iakyrus
NRRL B3457 Streptomyces paradoxus
NRRL B5427 Streptomyces violaceochromogenes
OE99
NRRL B1988 Streptomyces hawaiiensis
NRRL B12230 Streptomyces purpurascens
NRRL B3631 Streptomyces tuirus
NRRL ISP5293 Streptomyces arenae
NRRL B12422 Streptomyces luteogriseus
NRRL B24307 Streptomyces beijiangensis
NRRL B5312 Streptomyces griseoflavus
NRRL B1315 Streptomyces griseoluteus
NRRL B3811 Streptomyces recifensis
NRRL B24310 Streptomyces seoulensis
NRRL B12173 Streptomyces flaviscleroticus
NRRL B12202 Streptomyces minutiscleroticus
NRRL B16380 Streptomyces variegatus
NRRL B2072 Streptomyces melanogenes
NRRL B12152 Streptomyces noboritoensis
NRRL B24448 Streptomyces polyantibioticus
NRRL B65268 Streptomyces aridus

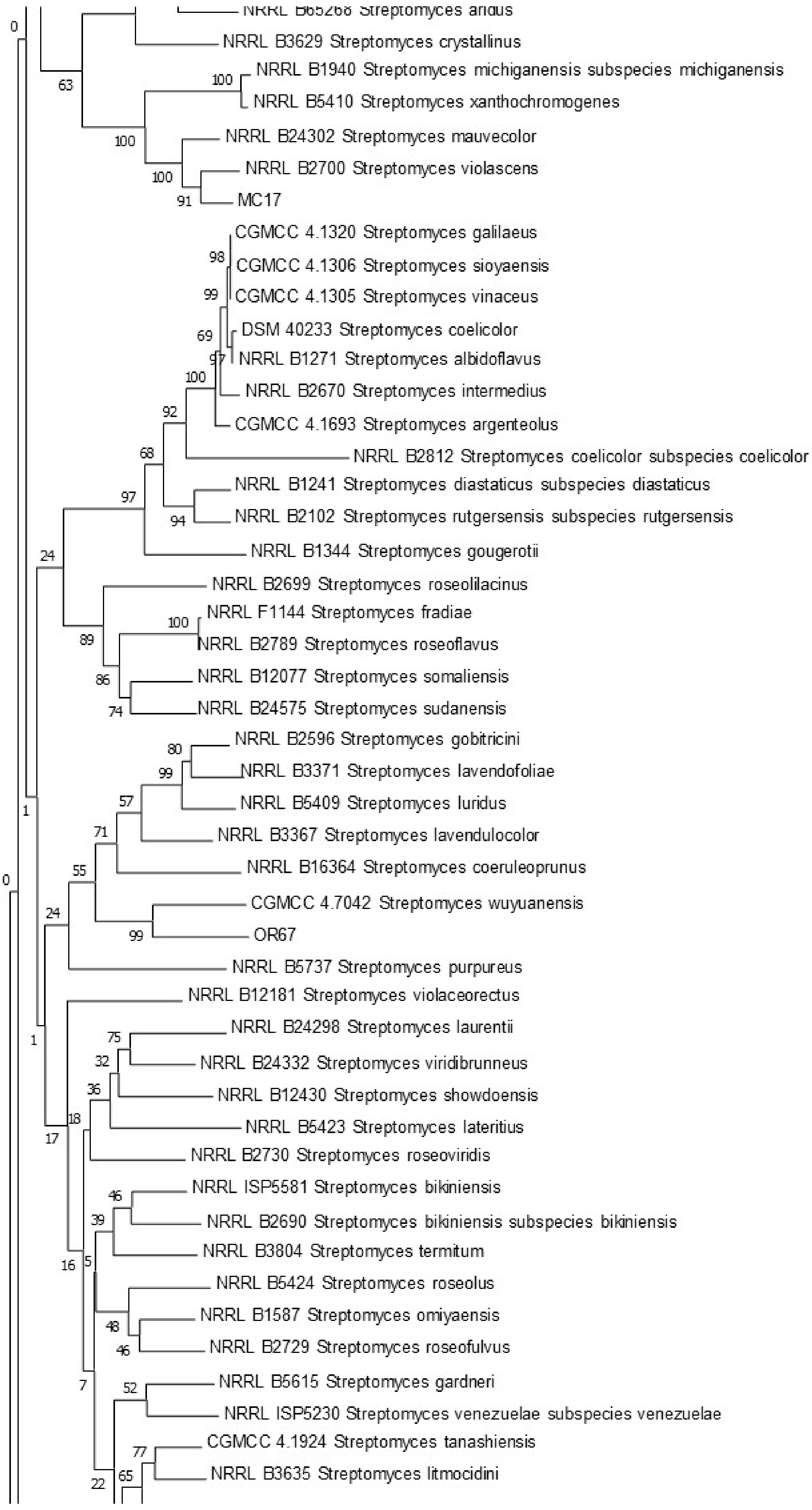
NRRL B3629 Streptomyces crystallinus
NRRL B1940 Streptomyces michiganensis subspecies michiganensis
NRRL B5410 Streptomyces xanthochromogenes
NRRL B24302 Streptomyces mauvecolor
NRRL B2700 Streptomyces violascens
MC17
CGMCC 4.1320 Streptomyces galilaeus
CGMCC 4.1306 Streptomyces sioyaensis
CGMCC 4.1305 Streptomyces vinaceus
DSM 40233 Streptomyces coelicolor
NRRL B1271 Streptomyces albidoflavus
NRRL B2670 Streptomyces intermedius
CGMCC 4.1693 Streptomyces argenteolus
NRRL B2812 Streptomyces coelicolor subspecies coelicolor
NRRL B1241 Streptomyces diastaticus subspecies diastaticus
NRRL B2102 Streptomyces rutgersensis subspecies rutgersensis
NRRL B1344 Streptomyces gougerotii
NRRL B2699 Streptomyces roseolilacinus
NRRL F1144 Streptomyces fradiae
NRRL B2789 Streptomyces roseoflavus
NRRL B12077 Streptomyces somaliensis
NRRL B24575 Streptomyces sudanensis
NRRL B2596 Streptomyces gobitricini
NRRL B3371 Streptomyces lavendofoliae
NRRL B5409 Streptomyces luridus
NRRL B3367 Streptomyces lavendulocolor
NRRL B16364 Streptomyces coeruleoprunus
CGMCC 4.7042 Streptomyces wuyuanensis
OR67
NRRL B5737 Streptomyces purpureus
NRRL B12181 Streptomyces violaceorectus
NRRL B24298 Streptomyces laurentii
NRRL B24332 Streptomyces viridibrunneus
NRRL B12430 Streptomyces showdoensis
NRRL B5423 Streptomyces lateritius
NRRL B2730 Streptomyces roseoviridis
NRRL ISP5581 Streptomyces bikiniensis
NRRL B2690 Streptomyces bikiniensis subspecies bikiniensis
NRRL B3804 Streptomyces termitum
NRRL B5424 Streptomyces roseolus
NRRL B1587 Streptomyces omiyaensis
NRRL B2729 Streptomyces roseofulvus
NRRL B5615 Streptomyces gardneri
NRRL ISP5230 Streptomyces venezuelae subspecies venezuelae
CGMCC 4.1924 Streptomyces tanashiensis
NRRL B3635 Streptomyces litmocidini

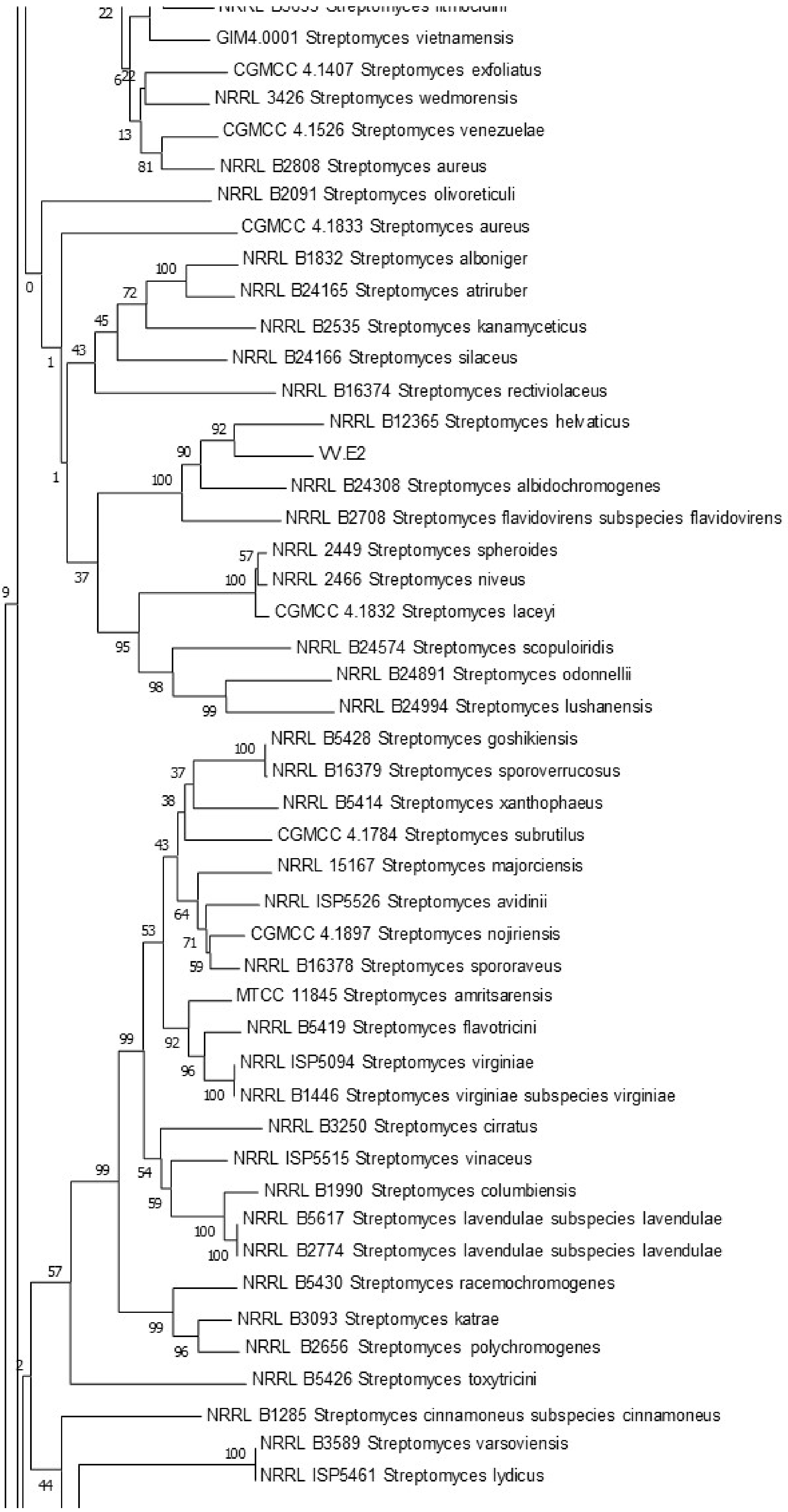

GIM4.0001 Streptomyces vietnamensis
CGMCC 4.1407 Streptomyces exfoliatus
NRRL 3426 Streptomyces wedmorensis
CGMCC 4.1526 Streptomyces venezuelae
NRRL B2808 Streptomyces aureus
NRRL B2091 Streptomyces olivoreticuli
CGMCC 4.1833 Streptomyces aureus
NRRL B1832 Streptomyces alboniger
NRRL B24165 Streptomyces atriruber
NRRL B2535 Streptomyces kanamyceticus
NRRL B24166 Streptomyces silaceus
NRRL B16374 Streptomyces rectiviolaceus
NRRL B12365 Streptomyces helvaticus
VV.E2
NRRL B24308 Streptomyces albidochromogenes
NRRL B2708 Streptomyces flavidovirens subspecies flavidovirens
NRRL 2449 Streptomyces spheroides
NRRL 2466 Streptomyces niveus
CGMCC 4.1832 Streptomyces laceyi
NRRL B24574 Streptomyces scopuloiridis
NRRL B24891 Streptomyces odonnellii
NRRL B24994 Streptomyces lushanensis
NRRL B5428 Streptomyces goshikiensis
NRRL B16379 Streptomyces sporoverrucosus
NRRL B5414 Streptomyces xanthophaeus
CGMCC 4.1784 Streptomyces subrutilus
NRRL 15167 Streptomyces majorciensis
NRRL ISP5526 Streptomyces avidinii
CGMCC 4.1897 Streptomyces nojiriensis
NRRL B16378 Streptomyces spororaveus
MTCC 11845 Streptomyces amritsarensis
NRRL B5419 Streptomyces flavotricini
NRRL ISP5094 Streptomyces virginiae
NRRL B1446 Streptomyces virginiae subspecies virginiae
NRRL B3250 Streptomyces cirratus
NRRL ISP5515 Streptomyces vinaceus
NRRL B1990 Streptomyces columbiensis
NRRL B5617 Streptomyces lavendulae subspecies lavendulae
NRRL B2774 Streptomyces lavendulae subspecies lavendulae
NRRL B5430 Streptomyces racemochromogenes
NRRL B3093 Streptomyces katrae
NRRL B2656 Streptomyces polychromogenes
NRRL B5426 Streptomyces toxytricini
NRRL B1285 Streptomyces cinnamoneus subspecies cinnamoneus
NRRL B3589 Streptomyces varsoviensis
NRRL ISP5461 Streptomyces lydicus

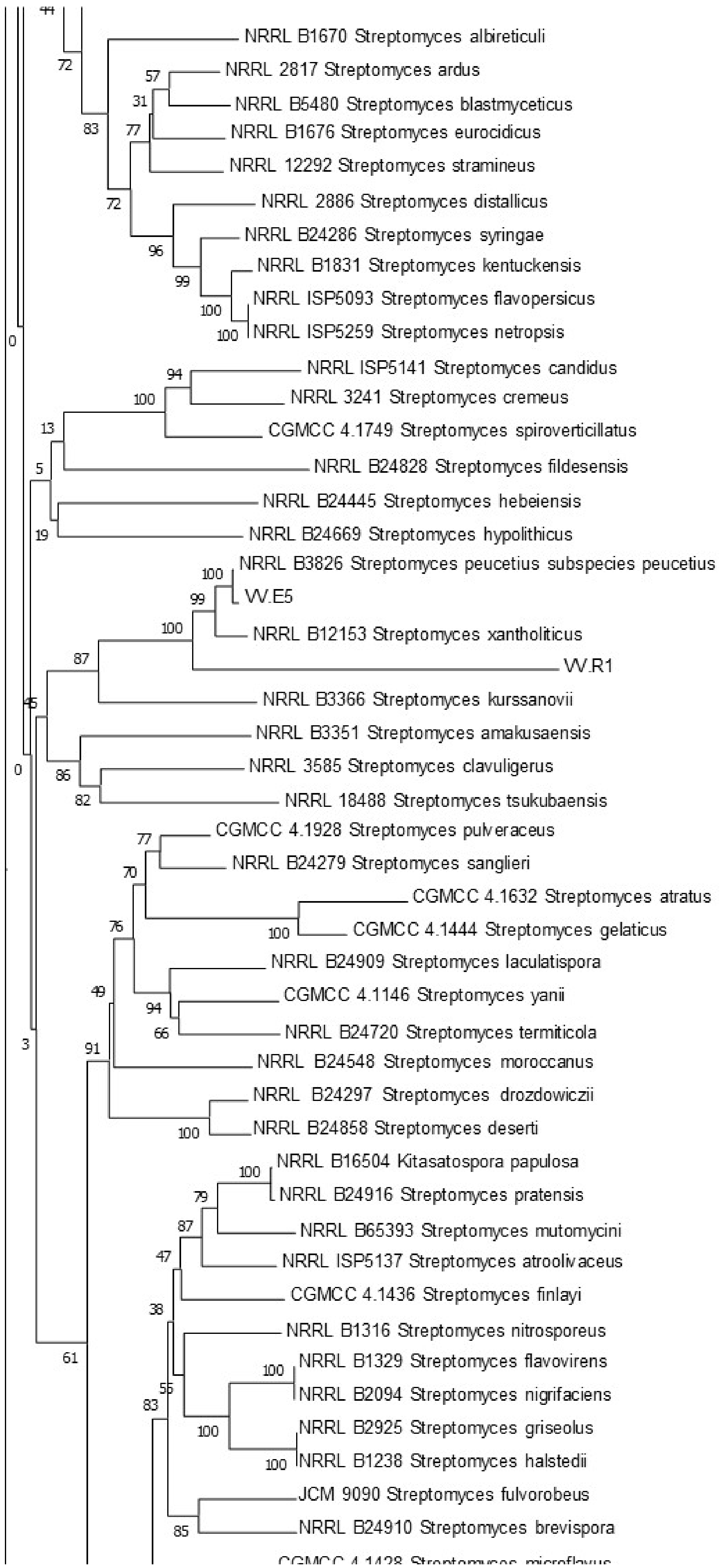

NRRL B1670 Streptomyces albireticuli
NRRL 2817 Streptomyces ardus
NRRL B5480 Streptomyces blastmyceticus
NRRL B1676 Streptomyces eurocidicus
NRRL 12292 Streptomyces stramineus
NRRL 2886 Streptomyces distallicus
NRRL B24286 Streptomyces syringae
NRRL B1831 Streptomyces kentuckensis
NRRL ISP5093 Streptomyces flavopersicus
NRRL ISP5259 Streptomyces netropsis
NRRL ISP5141 Streptomyces candidus
NRRL 3241 Streptomyces cremeus
CGMCC 4.1749 Streptomyces spiroverticillatus
NRRL B24828 Streptomyces fildesensis
NRRL B24445 Streptomyces hebeiensis
NRRL B24669 Streptomyces hypolithicus
NRRL B3826 Streptomyces peucetius subspecies peucetius
W.E5
NRRL B12153 Streptomyces xantholiticus
W.R1
NRRL B3366 Streptomyces kurssanovii
NRRL B3351 Streptomyces amakusaensis
NRRL 3585 Streptomyces clavuligerus
NRRL 18488 Streptomyces tsukubaensis
CGMCC 4.1928 Streptomyces pulveraceus
NRRL B24279 Streptomyces sanglieri
CGMCC 4.1632 Streptomyces atratus
CGMCC 4.1444 Streptomyces gelaticus
NRRL B24909 Streptomyces laculatispora
CGMCC 4.1146 Streptomyces yanii
NRRL B24720 Streptomyces termiticola
NRRL B24548 Streptomyces moroccanus
NRRL B24297 Streptomyces drozdowiczii
NRRL B24858 Streptomyces deserti
NRRL B16504 Kitasatospora papulosa
NRRL B24916 Streptomyces pratensis
NRRL B65393 Streptomyces mutomycini
NRRL ISP5137 Streptomyces atroolivaceus
CGMCC 4.1436 Streptomyces finlayi
NRRL B1316 Streptomyces nitrosporeus
NRRL B1329 Streptomyces flavovirens
NRRL B2094 Streptomyces nigrifaciens
NRRL B2925 Streptomyces griseolus
NRRL B1238 Streptomyces halstedii
JCM 9090 Streptomyces fulvorobeus
NRRL B24910 Streptomyces brevispora

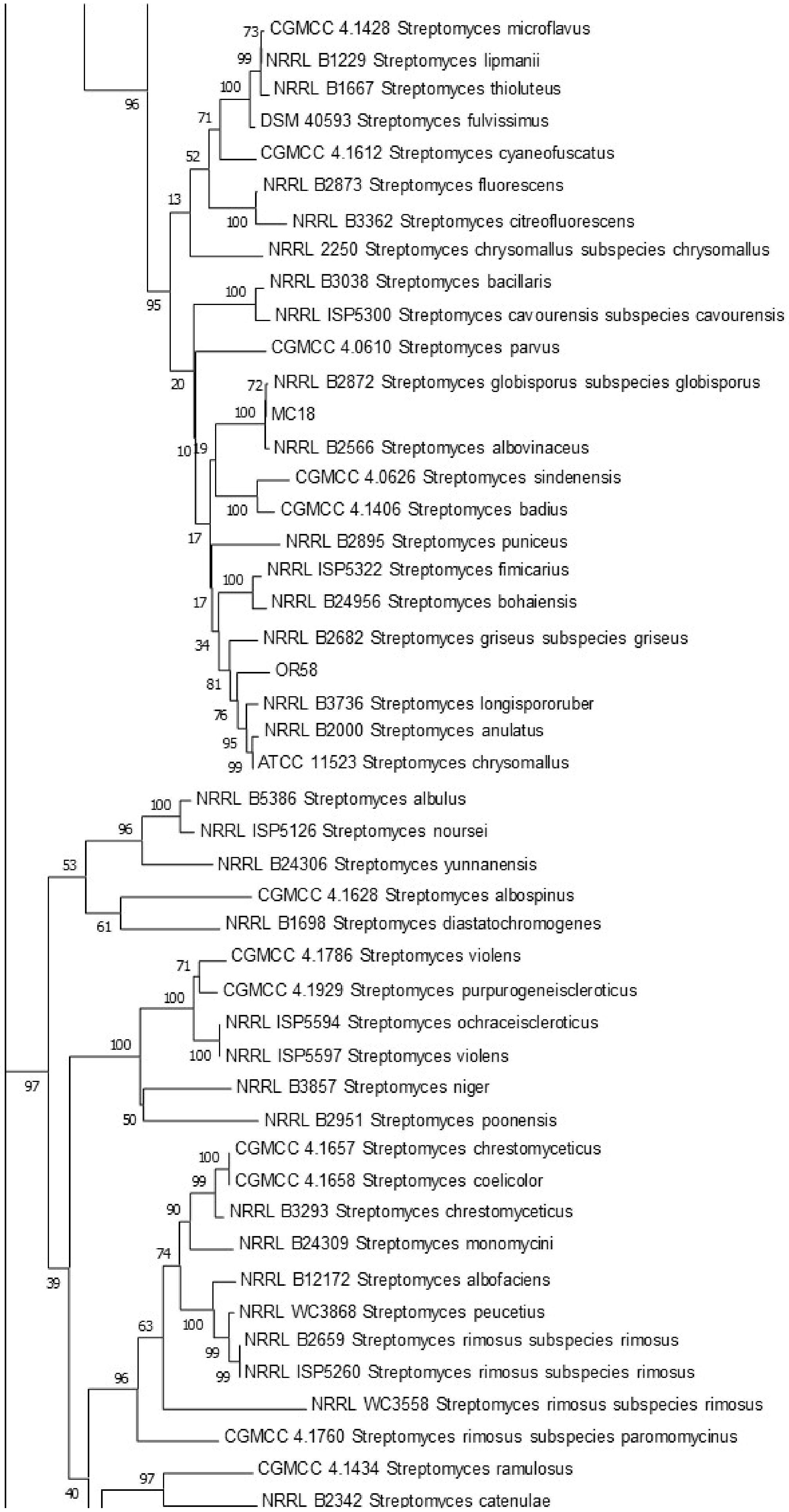
CGMCC 4.1428 Streptomyces microflavus
NRRL B1229 Streptomyces lipmanii
NRRL B1667 Streptomyces thioluteus
DSM 40593 Streptomyces fulvissimus
CGMCC 4.1612 Streptomyces cyaneofuscatus
NRRL B2873 Streptomyces fluorescens
NRRL B3362 Streptomyces citreofluorescens
NRRL 2250 Streptomyces chrysomallus subspecies chrysomallus
NRRL B3038 Streptomyces bacillaris
NRRL ISP5300 Streptomyces cavourensis subspecies cavourensis
CGMCC 4.0610 Streptomyces parvus
NRRL B2872 Streptomyces globisporus subspecies globisporus
MC18
NRRL B2566 Streptomyces albovinaceus
CGMCC 4.0626 Streptomyces sindenensis
CGMCC 4.1406 Streptomyces badius
NRRL B2895 Streptomyces puniceus
NRRL ISP5322 Streptomyces fimicarius
NRRL B24956 Streptomyces bohaiensis
NRRL B2682 Streptomyces griseus subspecies griseus
OR58
NRRL B3736 Streptomyces longispororuber
NRRL B2000 Streptomyces anulatus
ATCC 11523 Streptomyces chrysomallus
NRRL B5386 Streptomyces albulus
NRRL ISP5126 Streptomyces noursei
NRRL B24306 Streptomyces yunnanensis
CGMCC 4.1628 Streptomyces albospinus
NRRL B1698 Streptomyces diastatochromogenes
CGMCC 4.1786 Streptomyces violens
CGMCC 4.1929 Streptomyces purpurogeneiscleroticus
NRRL ISP5594 Streptomyces ochraceiscleroticus
NRRL ISP5597 Streptomyces violens
NRRL B3857 Streptomyces niger
NRRL B2951 Streptomyces poonensis
CGMCC 4.1657 Streptomyces chrestomyceticus
CGMCC 4.1658 Streptomyces coelicolor
NRRL B3293 Streptomyces chrestomyceticus
NRRL B24309 Streptomyces monomycini
NRRL B12172 Streptomyces albofaciens
NRRL WC3868 Streptomyces peucetius
NRRL B2659 Streptomyces rimosus subspecies rimosus
NRRL ISP5260 Streptomyces rimosus subspecies rimosus
NRRL WC3558 Streptomyces rimosus subspecies rimosus
CGMCC 4.1760 Streptomyces rimosus subspecies paromomycinus
CGMCC 4.1434 Streptomyces ramulosus
NRRL B2342 Streptomyces catenulae

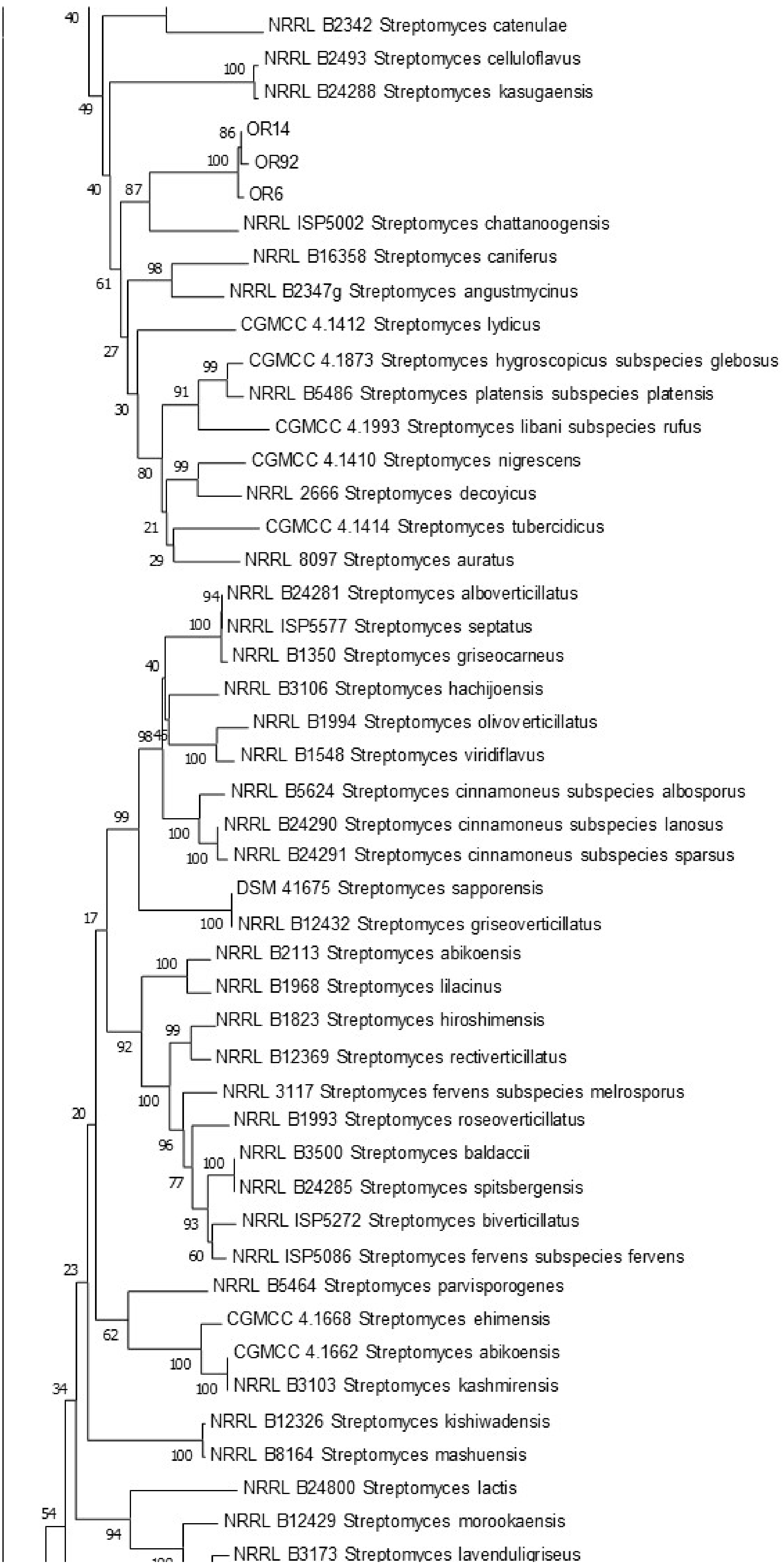
NRRL B2342 Streptomyces catenulae
NRRL B2493 Streptomyces celluloflavus
NRRL B24288 Streptomyces kasugaensis
OR14
OR92
OR6
NRRL ISP5002 Streptomyces chattanoogensis
NRRL B16358 Streptomyces caniferus
NRRL B2347g Streptomyces angustmycinus
CGMCC 4.1412 Streptomyces lydicus
CGMCC 4.1873 Streptomyces hygroscopicus subspecies glebosus
NRRL B5486 Streptomyces platensis subspecies platensis
CGMCC 4.1993 Streptomyces libani subspecies rufus
CGMCC 4.1410 Streptomyces nigrescens
NRRL 2666 Streptomyces decoyicus
CGMCC 4.1414 Streptomyces tubercidicus
NRRL 8097 Streptomyces auratus
NRRL B24281 Streptomyces alboverticillatus
NRRL ISP5577 Streptomyces septatus
NRRL B1350 Streptomyces griseocarneus
NRRL B3106 Streptomyces hachijoensis
NRRL B1994 Streptomyces olivoverticillatus
NRRL B1548 Streptomyces viridiflavus
NRRL B5624 Streptomyces cinnamoneus subspecies albosporus
NRRL B24290 Streptomyces cinnamoneus subspecies lanosus
NRRL B24291 Streptomyces cinnamoneus subspecies sparsus
DSM 41675 Streptomyces sapporensis
NRRL B12432 Streptomyces griseoverticillatus
NRRL B2113 Streptomyces abikoensis
NRRL B1968 Streptomyces lilacinus
NRRL B1823 Streptomyces hiroshimensis
NRRL B12369 Streptomyces rectiverticillatus
NRRL 3117 Streptomyces fervens subspecies melrosporus
NRRL B1993 Streptomyces roseoverticillatus
NRRL B3500 Streptomyces baldaccii
NRRL B24285 Streptomyces spitsbergensis
NRRL ISP5272 Streptomyces biverticillatus
NRRL ISP5086 Streptomyces fervens subspecies fervens
NRRL B5464 Streptomyces parvisporogenes
CGMCC 4.1668 Streptomyces ehimensis
CGMCC 4.1662 Streptomyces abikoensis
NRRL B3103 Streptomyces kashmirensis
NRRL B12326 Streptomyces kishiwadensis
NRRL B8164 Streptomyces mashuensis
NRRL B24800 Streptomyces lactis
NRRL B12429 Streptomyces morookaensis
NRRL B3173 Streptomyces lavenduligriseus

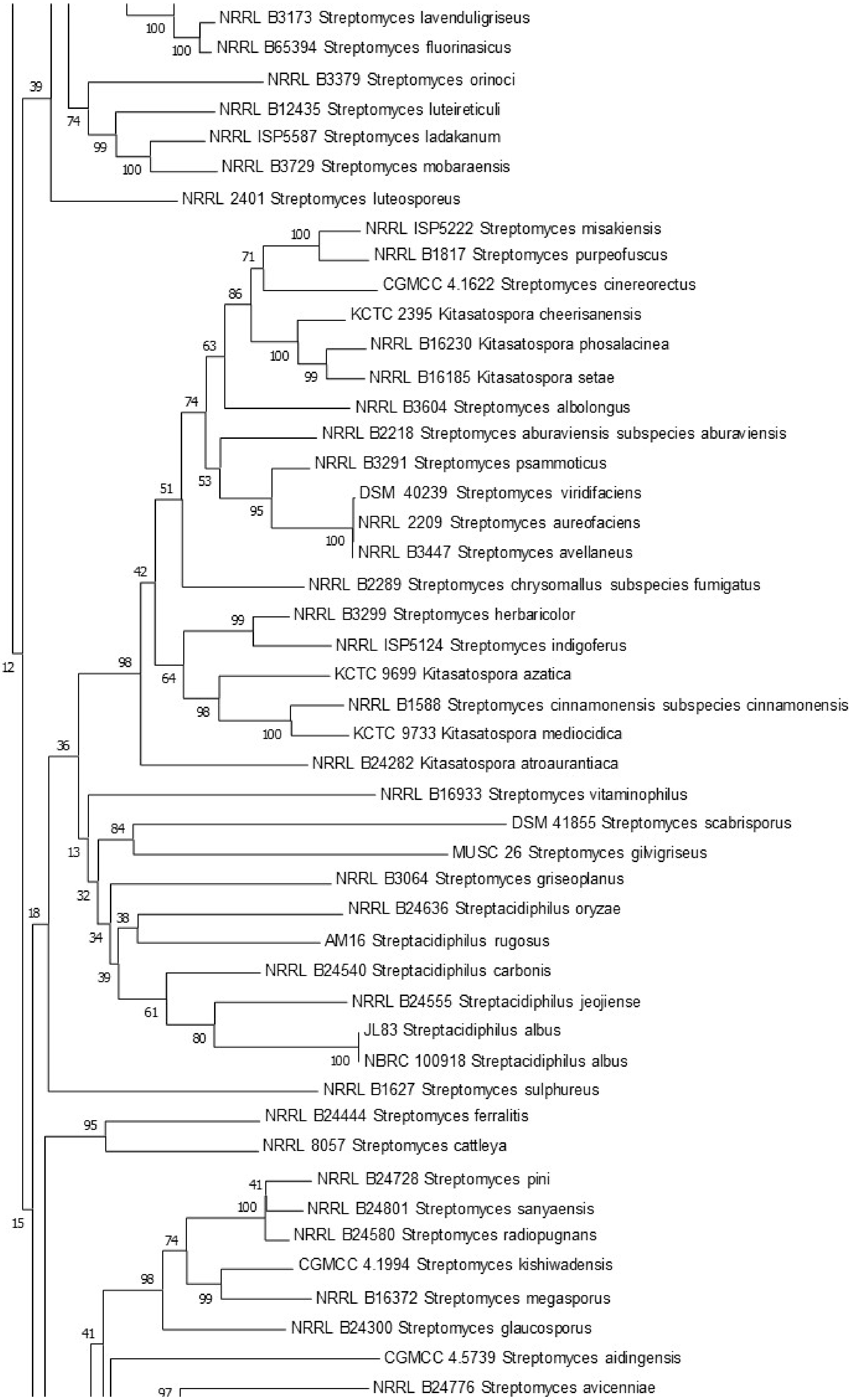
NRRL B3173 Streptomyces lavenduligriseus
NRRL B65394 Streptomyces fluorinasicus
NRRL B3379 Streptomyces orinoci
NRRL B12435 Streptomyces luteireticuli
NRRL ISP5587 Streptomyces ladakanum
NRRL B3729 Streptomyces mobaraensis
NRRL 2401 Streptomyces luteosporeus
NRRL ISP5222 Streptomyces misakiensis
NRRL B1817 Streptomyces purpeofuscus
CGMCC 4.1622 Streptomyces cinereorectus
KCTC 2395 Kitasatospora cheerisanensis
NRRL B16230 Kitasatospora phosalacinea
NRRL B16185 Kitasatospora setae
NRRL B3604 Streptomyces albolongus
NRRL B2218 Streptomyces aburaviensis subspecies aburaviensis
NRRL B3291 Streptomyces psammoticus
DSM 40239 Streptomyces viridifaciens
NRRL 2209 Streptomyces aureofaciens
NRRL B3447 Streptomyces avellaneus
NRRL B2289 Streptomyces chrysomallus subspecies fumigatus
NRRL B3299 Streptomyces herbaricolor
NRRL ISP5124 Streptomyces indigoferus
KCTC 9699 Kitasatospora azatica
NRRL B1588 Streptomyces cinnamonensis subspecies cinnamonensis
KCTC 9733 Kitasatospora mediocidica
NRRL B24282 Kitasatospora atroaurantiaca
NRRL B16933 Streptomyces vitaminophilus
DSM 41855 Streptomyces scabrisporus
MUSC 26 Streptomyces gilvigriseus
NRRL B3064 Streptomyces griseoplanus
NRRL B24636 Streptacidiphilus oryzae
AM16 Streptacidiphilus rugosus
NRRL B24540 Streptacidiphilus carbonis
NRRL B24555 Streptacidiphilus jeojiense
JL83 Streptacidiphilus albus
NBRC 100918 Streptacidiphilus albus
NRRL B1627 Streptomyces sulphureus
NRRL B24444 Streptomyces ferralitis
NRRL 8057 Streptomyces cattleya
NRRL B24728 Streptomyces pini
NRRL B24801 Streptomyces sanyaensis
NRRL B24580 Streptomyces radiopugnans
CGMCC 4.1994 Streptomyces kishiwadensis
NRRL B16372 Streptomyces megasporus
NRRL B24300 Streptomyces glaucosporus
CGMCC 4.5739 Streptomyces aidingensis
NRRL B24776 Streptomyces avicenniae

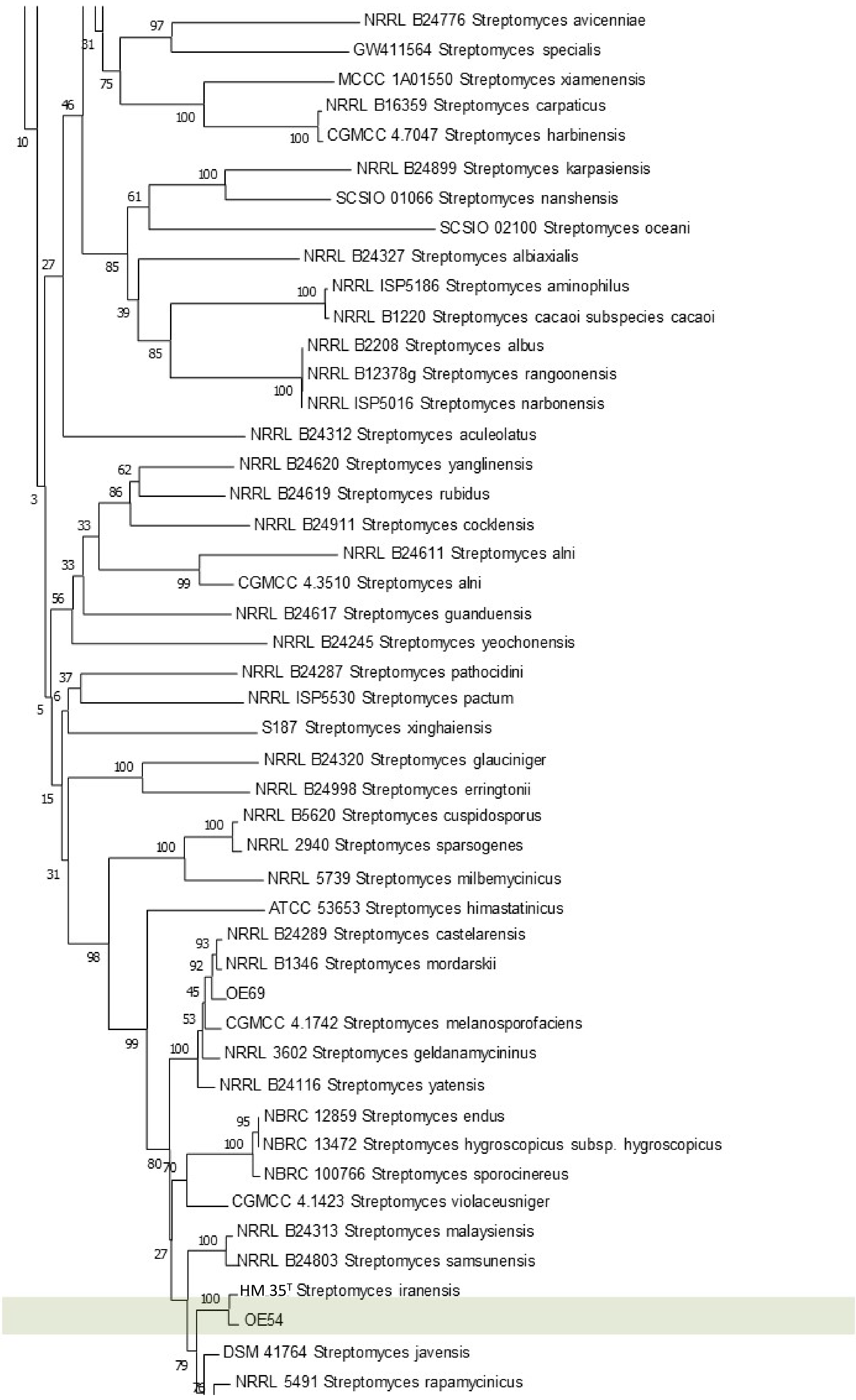
NRRL B24776 Streptomyces avicenniae
GW411564 Streptomyces specialis
MCCC 1A01550 Streptomyces xiamenensis
NRRL B16359 Streptomyces carpaticus
CGMCC 4.7047 Streptomyces harbinensis
NRRL B24899 Streptomyces karpasiensis
SCSIO 01066 Streptomyces nanshensis
SCSIO 02100 Streptomyces oceani
NRRL B24327 Streptomyces albiaxialis
NRRL ISP5186 Streptomyces aminophilus
NRRL B1220 Streptomyces cacaoi subspecies cacaoi
NRRL B2208 Streptomyces albus
NRRL B12378g Streptomyces rangoonensis
NRRL ISP5016 Streptomyces narbonensis
NRRL B24312 Streptomyces aculeolatus
NRRL B24620 Streptomyces yanglinensis
NRRL B24619 Streptomyces rubidus
NRRL B24911 Streptomyces cocklensis
NRRL B24611 Streptomyces alni
CGMCC 4.3510 Streptomyces alni
NRRL B24617 Streptomyces guanduensis
NRRL B24245 Streptomyces yeochonensis
NRRL B24287 Streptomyces pathocidini
NRRL ISP5530 Streptomyces pactum
S187 Streptomyces xinghaiensis
NRRL B24320 Streptomyces glauciniger
NRRL B24998 Streptomyces erringtonii
NRRL B5620 Streptomyces cuspidosporus
NRRL 2940 Streptomyces sparsogenes
NRRL 5739 Streptomyces milbemycinicus
ATCC 53653 Streptomyces himastatinicus
NRRL B24289 Streptomyces castelarensis
NRRL B1346 Streptomyces mordarskii
OE69
CGMCC 4.1742 Streptomyces melanosporofaciens
NRRL 3602 Streptomyces geldanamycininus
NRRL B24116 Streptomyces yatensis
NBRC 12859 Streptomyces endus
NBRC 13472 Streptomyces hygroscopicus subsp. hygroscopicus
NBRC 100766 Streptomyces sporocinereus
CGMCC 4.1423 Streptomyces violaceusniger
NRRL B24313 Streptomyces malaysiensis
NRRL B24803 Streptomyces samsunensis
HM 35T Streptomyces iranensis
OE54
DSM 41764 Streptomyces javensis
NRRL 5491 Streptomyces rapamycinicus

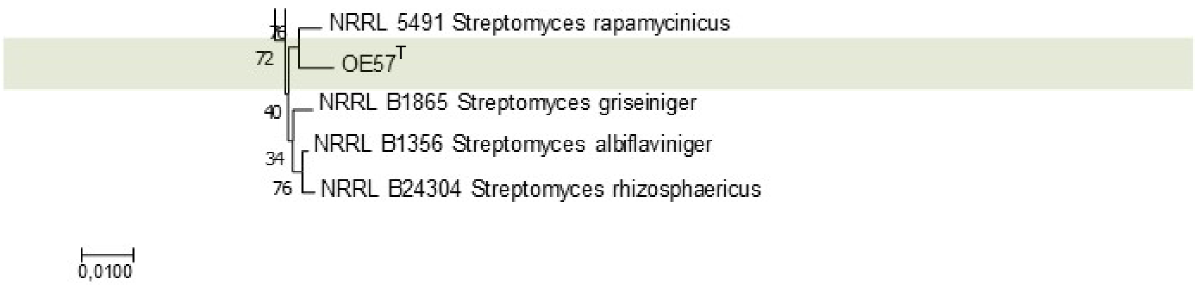

Figure S1. *Streptomyces* phylogenetic tree inferred from concatened partial sequences of the housekeeping genes (*atpD*, *gyrB*, *recA*, *rpoB*, and *trpB*) of endophytic OE54 and OE57^T strains with type strains obtained from the ARS Microbial Genomic Sequence Database server.
