# Supplementary material for: Rapamycin Plays a Pivotal Role in the Potent Antifungal Activity Exhibited Against Verticillium dahliae by Streptomyces iranensis OE54 and Streptomyces lacaronensis sp. nov. Isolated from Olive Roots
Source: Microorganisms. 2025 Jul 9;13(7):1622. doi: 10.3390/microorganisms13071622 (PMC12298158; doi:10.3390/microorganisms13071622)
Supplement: Supplementary file 1 [file microorganisms-13-01622-s001.zip › Supplementary Table S1.pdf]

Table S1. Inferred functions of *rap* genes and ORFs located in the rapamycin biosynthetic clusters of *S. rapamycinicus* B-NRRL 5491[T], *S. iranensis* DSM 41954[T], OE54, and OE57[T] strains. Proposed functions are based on available literature and functional annotations obtained from the NCBI (X86780.1) and MIBiG (BGC0001040) databases, as well as homology analyses using InterPro and BLAST

Gene	Microorganism	Size (aa)	Proposed function	Reference
*orf*ZZ	*S. rapamycinicus*	290	Monosaccharide transporter	Molnár *et al*. (1996)
orfZ	*S. rapamycinicus*	389	Hypothetical protein	
*rap*Y	*S. rapamycinicus*	204	TetR family transcriptional regulators (negative regulation)	Yoo *et al*. (2015)
*rap*X	All*	235	ABC-transporter; its expression is repressed by *rapS* and *rapY*	Molnár *et al*. (1996)
*orf*W	All*	459	MacB-like ABC transporter, putative efflux pump	Molnár *et al*. (1996)
*orf*V	All*	437	Membrane transport protein	Molnár *et al*. (1996)
*rap*R	All*	220	Response regulator (RapR-RapS two-component system) (negative regulation)	Yoo *et al*. (2015)
*rap*S	All*	399	Histidine Kinase (RapR-RapS two-component system) (negative regulation)	Yoo *et al*. (2015)
*orf*U	*S. rapamycinicus*	200	Hypothetical protein	
*rap*T	*S. rapamycinicus*	264	Ketoreductase/dehydrogenase	Molnár *et al*. (1996)
*rap*B	All*	10223	Polyketide synthase	Schwecke *et a*l. (1995)
*rap*A	All*	8563	Polyketide synthase	Schwecke *et a*l. (1995)
*rap*P	All*	1541	Pipecolate incorporating enzyme; condenses the linear polyketide chain	Schwecke *et al*. (1995); König *et al.* (1997)
*rap*C	All*	6260	Polyketide synthase	Schwecke *et a*l. (1995)
*rap*Q	All*	211	SAM-dependent *O*-Methyltransferase	Molnár *et al*. (1996)
*rap*O	All*	77	Ferredoxin	Schwecke et al. (1995)
*rap*N	All*	404	Cytochrome P450 monooxygenase	Schwecke et al. (1995)
*rap*M	All*	317	SAM-dependent *O*-Methyltransferase	Molnár *et al*. (1996)
*rap*L	All*	343	Lysine cyclodeaminase; biosynthesis of pipecolate	Molnár *et al*. (1996); Gatto et al. (2006)
*rap*K	All*	334	Pteridine-dependent dioxygenase	Molnár et al. (1996)
*rap*J	All*	386	Cytochrome P450 monooxygenase	Schwecke *et a*l. (1995)
*rap*I	All*	260	SAM-dependent *O*-Methyltransferase	Molnár *et a*l. (1996)
*rap*H	All*	872	LuxR-family transcriptional activator (positive regulator)	Kuščer *et a*l. (2007)
*rap*G	All*	330	AraC-family transcriptional activator (positive regulator)	Kuščer *et al*. (2007)

*rap*F	All*	453	Major facilitator superfamily transporter	Schwecke *et al.* (1995); this work
*orf*E	All*	465	ATP-binding protein, putative glutathione synthetase-like	This work
orfD	All*	387	Hypothetical protein	
*orf*DD	All*	207	Cystathionine synthase, pyridoxal phosphate-dependent	Molnár *et al*. (1996); this work
**Additional genes located in the clusters of *S. iranensis*, OE54 and OE57**				
*orf*1	All*	318	Cystathionine synthase, pyridoxal phosphate-dependent	This work
*orf*2	OE57	205	TecR/AcR family transcriptional regulator	This work
*orf*3	OE57	359	Methyltransferase	This work
*orf*4	OE57	535	Protein kinase domain-containing protein	This work
*orf*5	*S. iranensis*	467	Hypothetical protein	This work
*orf*6	*S. iranensis*, OE54	418	SGNL/GDSL hydrolase family protein	This work
*orf*7	*S. iranensis*, OE54	520	FAD-dependent monooxygenase	This work
*orf*8	*S. iranensis*, OE54	232	TecR/AcR family transcriptional regulator	This work

All* indicates that the corresponding gene has been identified in all mentioned strains
