# Supplementary material for: Rapamycin Plays a Pivotal Role in the Potent Antifungal Activity Exhibited Against Verticillium dahliae by Streptomyces iranensis OE54 and Streptomyces lacaronensis sp. nov. Isolated from Olive Roots
Source: Microorganisms. 2025 Jul 9;13(7):1622. doi: 10.3390/microorganisms13071622 (PMC12298158; doi:10.3390/microorganisms13071622)
Supplement: Supplementary file 1 [file microorganisms-13-01622-s001.zip › Supplementary Table S2.pdf]

Table S2. ^{13}C, ^{1}H Nuclear Magnetic Resonance (NMR) spectral data of rapamycin [δ (ppm), $CDCl_3$]

Position	Carbon type	^{13}C (δ)	^{1}H (δ)	Position	Carbon type	^{13}C (δ)	^{1}H (δ)
**1**	C=O	169.4		**27**	CH-OH	77.3	4.18
**2**	CH	57.2	5.29	**28**	C=C	136.2	
**3**	CH_2	27.2	1.77. 2.34	**29**	CH=C	126.9	5.40
**4**	CH_2	20.7	1.47. 1.78	**30**	CH	46.7	3.33
**5**	CH_2	25.4	1.48. 1.76	**31**	C=O	208.4	
**6**	CH_2	44.3	3.44. 3.60	**32**	CH_2	40.8	2.60. 2.74
**7**	C=O	166.9		**33**	CH-OCO	75.8	5.18
**8**	C=O	192.4		**34**	CH	33.3	1.98
**9**	O-C-OH	98.6		**35**	CH_2	38.4	1.12. 1.23
**10**	CH	33.8	1.98	**36**	CH	33.2	1.39
**11**	CH_2	27.4	1.60	**37**	CH_2	34.2	0.65. 2.10
**12**	CH_2	31.3	1.62. 1.33	**38**	CH-OCH_3	84.4	2.93
**13**	CH-OC	67.3	3.86	**39**	CH-OH	74.0	3.37
**14**	CH_2	38.9	1.52. 1.86	**40**	CH_2	31.3	1.33. 1.99
**15**	CH-OCH_3	84.5	3.67	**41**	CH_2	31.8	1.00. 1.70
**16**	C=C	135.6		**42**	11-CH_3	16.1	0.96
**17**	CH=C	129.8	5.98	**43**	17-CH_3	10.3	1.65
**18**	CH=C	126.5	6.40	**44**	23-CH3	21.5	1.05
**19**	CH=C	133.8	6.34	**45**	25-CH3	13.2	1.02
**20**	CH=C	130.3	6.16	**46**	29-CH3	13.2	1.74
**21**	CH=C	140.4	5.54	**47**	31-CH_3	16.1	1.12
**22**	CH	35.3	2.33	**48**	35-CH3	15.9	0.92
**23**	CH_2	40.2	1.20. 1.50	**49**	16-OCH_3	56.0	3.14
**24**	CH	41.6	2.75	**50**	27-OCH_3	59.6	3.34
**25**	C=O	215.9		**51**	39-OCH_3	56.5	3.42
**26**	CH-OCH_3	84.9	3.72				
