# Supplementary material for: Rapamycin Plays a Pivotal Role in the Potent Antifungal Activity Exhibited Against Verticillium dahliae by Streptomyces iranensis OE54 and Streptomyces lacaronensis sp. nov. Isolated from Olive Roots
Source: Microorganisms. 2025 Jul 9;13(7):1622. doi: 10.3390/microorganisms13071622 (PMC12298158; doi:10.3390/microorganisms13071622)
Supplement: Supplementary file 1 [file microorganisms-13-01622-s001.zip › Supplementary Table S3.pdf]

Table S3. ^{13}C, ^{1}H Nuclear Magnetic Resonance (NMR) spectral data of epoxinnamide [δ (ppm), $CDCl_3$]. Dht: 3,*β*-dihydroxytyrosine; Hgy: *α*-hydroxyglycine; Hle: *β*-hydroxyleucine; Hpg: hydroxyphenylglycine; EPCA: *O*-1,2-epoxypropyl cinnamic acid.

Position	^{13}C (δ)	^{1}H (δ)	Position	^{13}C (δ)	^{1}H (δ)
	**Asn**		29-OH		5.09
1	169.6		30	27.9	1.74
2	49	3.99	31	14.3	0.74
2-NH		8.23	32	20.2	0.75
3	35.1	2.61		**Gly**	
4	170.7		33	168.4	
	**Dht**		34a	42.6	4.18
5	170.6		34b		3.39
6	63.8	3.49	34-NH		7.68
6-NH		8.14		**Thr-1**	
7	69.8	4.69	35	170	
7-OH		6.15	36	57.7	4.44
8	132.3		36-NH		8.16
9	120.1	6.27	37	65.8	4.4
10	147.2		37-OH		4.52
11	147.4		38	20.3	1.08
11-OH		9.4		**Hpg**	
12	116.1	6.87	39	169.1	
13	120.2	7.08	40	60.1	4.83
	**Hgy**		41	131.6	
14	170.9		42	128.5	7.07
15	71.3	5.79	43	123.6	6.41
15-NH		9.07	44	159.6	
	**Leu**		45	122.3	7.18
16	171.5		46	131.3	7.37
17	50.3	4.64		**Thr-2**	
18a	42.6	1.45	47	173.2	
18b		1.31	48	61	5.03
19	24.1	1.46	49	68.7	5.19
20	23.1	0.88	50	17.2	1.23
21	21.8	0.86		**EPCA**	
	**Pro**		51	169.2	
22	171.7		52	124.6	6.94
23	60.2	4.37	53	136.7	8.11
24a	29.9	2.21	54	134.1	
24b		1.56	55	126.9	7.42
25a	24.7	1.79	56	127.8	7.02
26a	47.3	3.72	57	129.7	7.33
26b		3.52	58	124.4	7.09
	**Hle**		59	136.7	
27	170.1		60	54.8	4.07
28	52.5	4.59	61	58.9	2.97
28-NH		7.69	62	17.6	1.36
29	74.4	3.22			
