# Supplementary material for: Rapamycin Plays a Pivotal Role in the Potent Antifungal Activity Exhibited Against Verticillium dahliae by Streptomyces iranensis OE54 and Streptomyces lacaronensis sp. nov. Isolated from Olive Roots
Source: Microorganisms. 2025 Jul 9;13(7):1622. doi: 10.3390/microorganisms13071622 (PMC12298158; doi:10.3390/microorganisms13071622)
Supplement: Supplementary file 1 [file microorganisms-13-01622-s001.zip › Supplementary Table S4.pdf]

Table S4. Cultural and growth characteristics of strains OE54 and OE57^T, along with their closest phylogenomic relatives

Strain	*S. iranensis* DSM 41954^T	OE54	OE57^T	*S. rapamycinicus* DSM 41530^T		*S. iranensis* DSM 41954^T	OE54	OE57^T	*S. rapamycinicus* DSM 41530T
**Temperature tests (ºC)#**					**Media Tests#**				
4	-	-	-	-	ISP1	+++	+++	+++	++
10	-	-	-	-	Description ISP1	Signal white aerial mycelium (9003). Ochre yellow substrate mycelium (1024)	Signal white aerial mycelium (9003). Beige substrate mycelium (1001)	Signal white aerial mycelium (9003). Cream substrate mycelium (9001)	Cream aerial mycelium (9001). Beige substrate mycelium (1001)
15	++	+++	+	++	ISP2 (-$CaCO_3$)	+++	+++	+++	+++
20	+++	+++	+++	+++	Description ISP2	Signal white aerial mycelium (9003). Ochre yellow substrate mycelium (1024)	Traffic grey aerial mycelium (7042). Brown beige substrate mycelium (1011)	Signal white aerial mycelium (9003). Beige substrate mycelium (1001)	Cream aerial mycelium (9001). Beige substrate mycelium (1001)
25	+++	+++	+++	+++	ISP3	+++	+++	+++	+++
28	+++	+++	+++	+++	Description ISP3	Signal white aerial mycelium (9003). Sand yellow substrate mycelium (1002)	Traffic grey aerial mycelium (7042). Brown beige substrate mycelium (1011)	Light grey aerial mycelium (7035). Cream substrate mycelium (9001)	Cream aerial mycelium (9001). Beige substrate mycelium (1001)
30	+++	+++	+++	+++	ISP4	++	++	+++	++
37	++	++	++	+++	Description ISP4	Traffic grey aerial mycelium (7042). Brown beige substrate mycelium (1011)	Traffic grey aerial mycelium (7042). Brown beige substrate mycelium (1011)	Signal white aerial mycelium (9003). Cream substrate mycelium (9001)	Window grey aerial mycelium (7040). Beige substrate mycelium (1001)
42	-	-	++	+	ISP5	++	+	+	+
45	-	-	-	-	Description ISP5	Cream aerial mycelium (9001). Brown beige substrate mycelium (1011)	Cream aerial mycelium (9001). Brown beige substrate mycelium (1011)	Signal white aerial mycelium (9003). Cream substrate mycelium (9001)	Cream aerial mycelium (9001). Beige substrate mycelium (1001)
**pH tests**					ISP6	+	+	+	+
5.0	+++	+++	+++	+++	Description ISP6	Signal white aerial mycelium (9003). Beige substrate mycelium (1001).	Signal white aerial mycelium (9003). Beige substrate mycelium (1001).	Signal white aerial mycelium (9003). Beige substrate mycelium (1001).	Signal white aerial mycelium (9003). Beige substrate mycelium (1001).
5.5	+++	+++	+++	+++	ISP7	++	++	+	+
6.0	+++	+++	+++	+++	Description ISP7	Cream aerial mycelium (9001). Brown beige substrate mycelium (1011) with salmon range diffusible pigment (2012)	Cream aerial mycelium (9001). Brown beige substrate mycelium (1011) with salmon range diffusible pigment (2012)	Light grey aerial mycelium (7035). Beige substrate mycelium (1001) with salmon range diffusible pigment (2012)	Cream aerial mycelium (9001). Beige substrate mycelium (1001) with salmon range diffusible pigment (2012)
7.5	+++	+++	+++	+++	TSA	+	+	++	+

8.0	+++	+++	+++	+++	Description TSA	Brown beige substrate mycelium (1011)	Brown beige substrate mycelium (1011)	Cream aerial mycelium (9001). Beige substrate mycelium (1001)	Brown beige substrate mycelium (1011)
8.5	++	+++	+++	++	NA	+++	+++	+++	+
10.0	+	+++	+++	+	Description NA	Signal white aerial mycelium (9003). Cream substrate mycelium (9001)	Cream aerial mycelium (9001). Cream substrate mycelium (9001).	Signal white aerial mycelium (9003). Cream substrate mycelium (9001)	Cream substrate mycelium (9001)
12.0	-	++	-	+	Bennet media	+++	+++	+++	++
**NaCl tolerance***					Description Bennet Media	Signal white aerial mycelium (9003). Cream substrate mycelium (9001)	Platinum grey aerial mycelium (7036). Cream substrate mycelium (9001)	Lemon yellow substrate mycelium (1012)	Lemon yellow substrate mycelium (1012)
2.5%	+	+	+	+	R5	+++	+++	+++	+++
5.0%	-	-	+	+	Description R5	Signal white aerial mycelium (9003). Beige substrate mycelium (1001)	Signal white aerial mycelium (9003). Beige substrate mycelium (1001)	Maize yellow substrate mycelium (1006)	Maize yellow substrate mycelium (1006)
7.5%	-	-	-	-	GYM	+++	+++	+++	++
10%	-	-	-	-	Description GYM	Signal white aerial mycelium (9003). Ochre yellow substrate mycelium (1024)	Traffic grey aerial mycelium (7042). Brown beige substrate mycelium (1011)	Signal white aerial mycelium (9003). Brown beige substrate mycelium (1011)	Signal white aerial mycelium (9003). Brown beige substrate mycelium (1011)

+, weak growth; ++moderate growth; +++ good growth; - no growth

* +, growth; -, no growth
