# Supplementary material for: Rapamycin Plays a Pivotal Role in the Potent Antifungal Activity Exhibited Against Verticillium dahliae by Streptomyces iranensis OE54 and Streptomyces lacaronensis sp. nov. Isolated from Olive Roots
Source: Microorganisms. 2025 Jul 9;13(7):1622. doi: 10.3390/microorganisms13071622 (PMC12298158; doi:10.3390/microorganisms13071622)
Supplement: Supplementary file 1 [file microorganisms-13-01622-s001.zip › Supplementary Table S5.pdf]

Table S5. Phenotypic characteristics of strains OE54 and OE57, along with their closest phylogenomic relatives, as determined by API 20NE and API ZYM assays.

Substrates / Strain	*S. iranensis* DSM 41954^T	OE54	OE57^T	*S. rapamycinicus* DSM 41530^T		*S. iranensis* DSM 41954^T	OE54	OE57^T	*S. rapamycinicus* DSM 41530^T
**API 20NE**					**API ZYM**				
Tryptophan	-	-	-	-	Phosphatase alkaline	+	+	+	+
Glucose (fermentation)	-	-	-	-	Esterase C4	-	+	-	+
L-Arginine	-	-	-	-	Esterase (lipase) C8	+	+	-	+
Urea	-	+	-	-	Lipase C14	+	+	-	-
Esculin	+	+	+	+	Leucine arylamidase	+	+	+	+
Gelatine	+	+	+	+	Valine arylamidase	+	+	+	+
p-Nitro-phenyl-β-D-galactopyranosid	+	+	+	+	Cystine arylamidase	+	+	+	+
Glucose	+	+	+	+	Trypsin	+	+	+	+
Arabinose	+	+	+	+	α-chymotrypsin	+	+	+	+
Mannose	+	+	+	+	Acid phosphatase	+	+	+	+
Mannitol	+	+	+	+	Naphthol-AS-BI-phosphohydrolase	+	+	+	+
N-acetylglucosamine	+	+	+	+	α-galactosidase	+	+	-	+
Maltose	+	+	+	+	β-galactosidase	+	+	+	+
Potassium gluconate	+	+	+	+	β-glucuronidase	-	-	-	-
Capric acid	-	-	-	-	α-glucosidase	-	+	+	-
Adipic acid	+	+	+	+	β-glucosidase	+	+	+	+
Malic acid	+	+	+	+	n-Acetyl-β-glucosaminidase	+	+	+	+
Citric acid	-	-	-	-	α-mannosidase	+	+	-	+
Phenylacetic acid	-	-	-	-	α-fucosidase	-	-	-	-
