# Supplementary material for: Rapamycin Plays a Pivotal Role in the Potent Antifungal Activity Exhibited Against Verticillium dahliae by Streptomyces iranensis OE54 and Streptomyces lacaronensis sp. nov. Isolated from Olive Roots
Source: Microorganisms. 2025 Jul 9;13(7):1622. doi: 10.3390/microorganisms13071622 (PMC12298158; doi:10.3390/microorganisms13071622)
Supplement: Supplementary file 1 [file microorganisms-13-01622-s001.zip › Supplementary Table S6.pdf]

Table S6. Fatty acids profiles of the *Streptomyces lacaronensis* sp. nov. strain OE57^T (=DSM 118741^T) and its closest phylogenomic neighbor *Streptomyces rapamycinicus* DSM 41530^T

**Fatty acid compounds***	**DSM 118741^T**	**DSM 41530^T**
$C_{13:0}$ *iso*	1.1	1.1
$C_{14:0}$ *iso*	1.9	5.2
$C_{14:0}$	0.6	1.2
$C1_{5:0}$ *iso*	28.7	22.9
$C_{15:0}$ *anteiso*	6.9	10.5
$C_{15:0}$	0.7	1.5
$C_{16:0}$ *iso*	9.2	16.1
$C_{16:1}$ CIS 9	5.4	5.2
$C_{16:0}$	10.1	13.9
$C_{17:1}$ *iso* CIS 9	8.5	4.3
$C_{17:0}$ *iso*	17.4	7.5
$C_{17:0}$ *anteiso*	4.3	3.6

* Only fatty acids >1% are listed in the table.
