# Supplementary figures and images for: Rapamycin Plays a Pivotal Role in the Potent Antifungal Activity Exhibited Against Verticillium dahliae by Streptomyces iranensis OE54 and Streptomyces lacaronensis sp. nov. Isolated from Olive Roots
Source: Microorganisms. 2025 Jul 9;13(7):1622. doi: 10.3390/microorganisms13071622 (PMC12298158; doi:10.3390/microorganisms13071622)

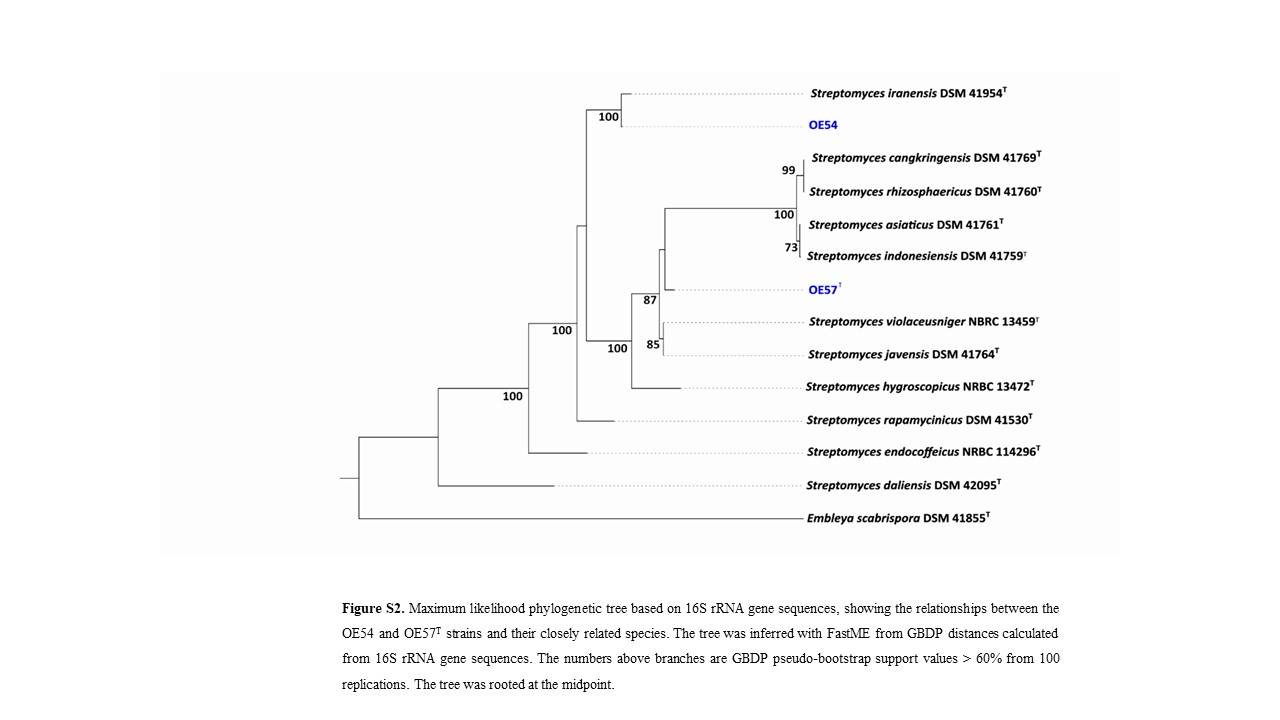

Supplement: Supplementary file 1 [file microorganisms-13-01622-s001.zip › Supplementary Figure S2.jpg]

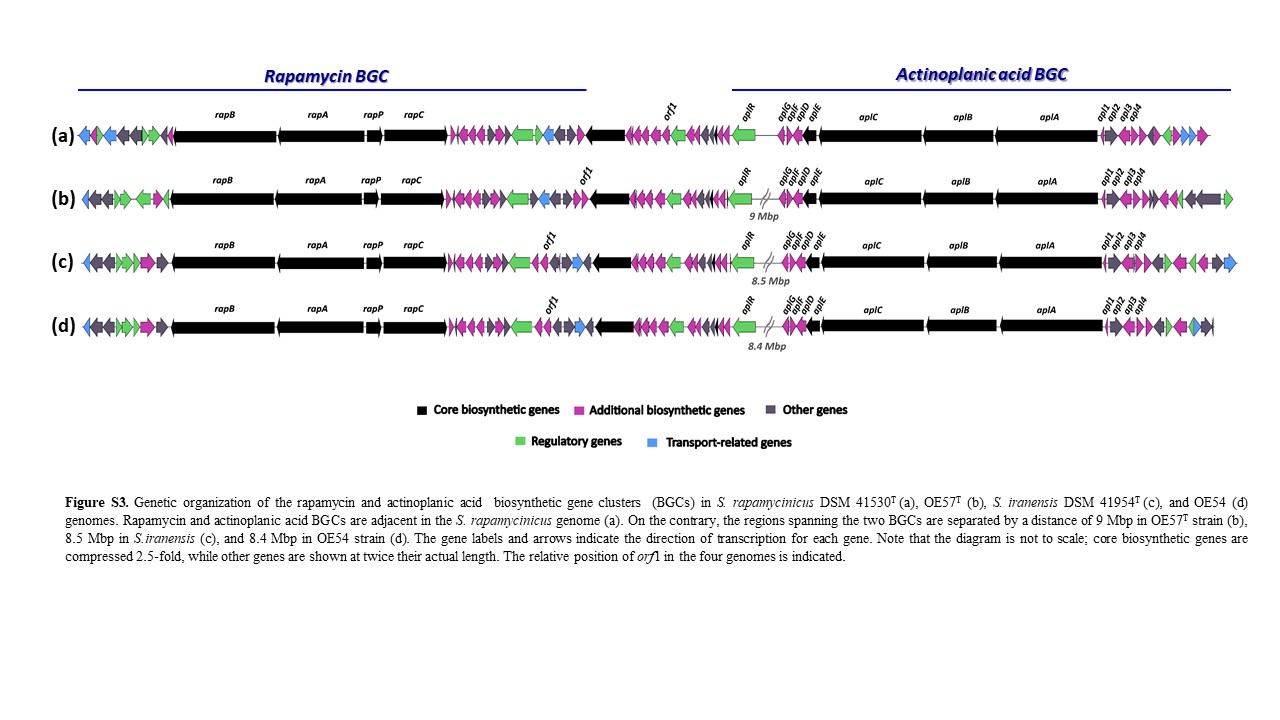

Supplement: Supplementary file 1 [file microorganisms-13-01622-s001.zip › Supplementary Figure S3.jpg]

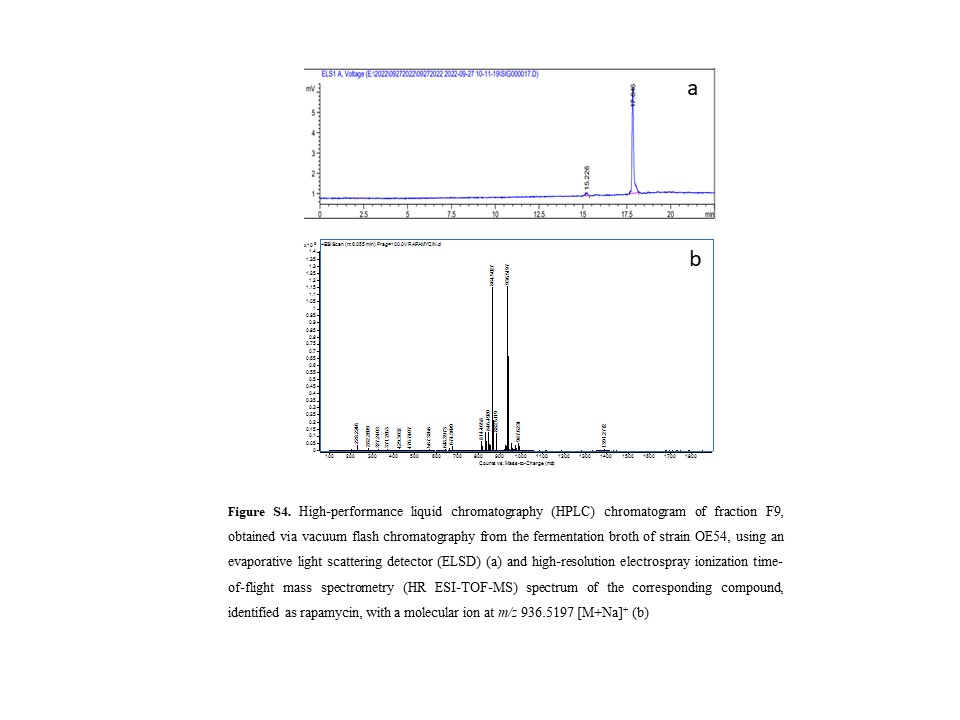

Supplement: Supplementary file 1 [file microorganisms-13-01622-s001.zip › Supplementary Figure S4.jpg]

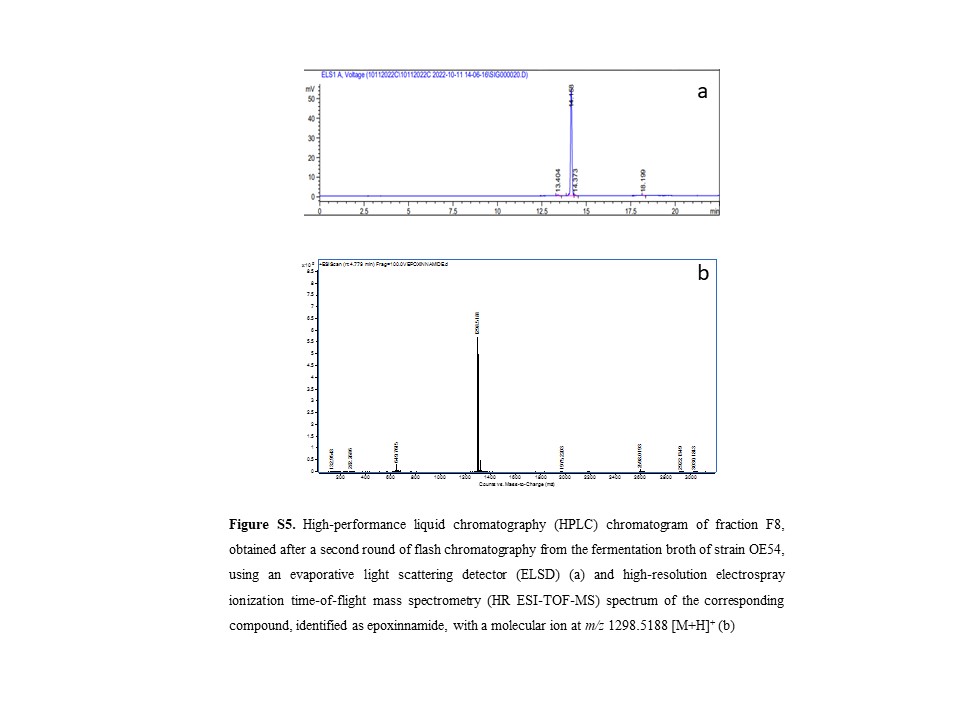

Supplement: Supplementary file 1 [file microorganisms-13-01622-s001.zip › Supplementary Figure S5.jpg]

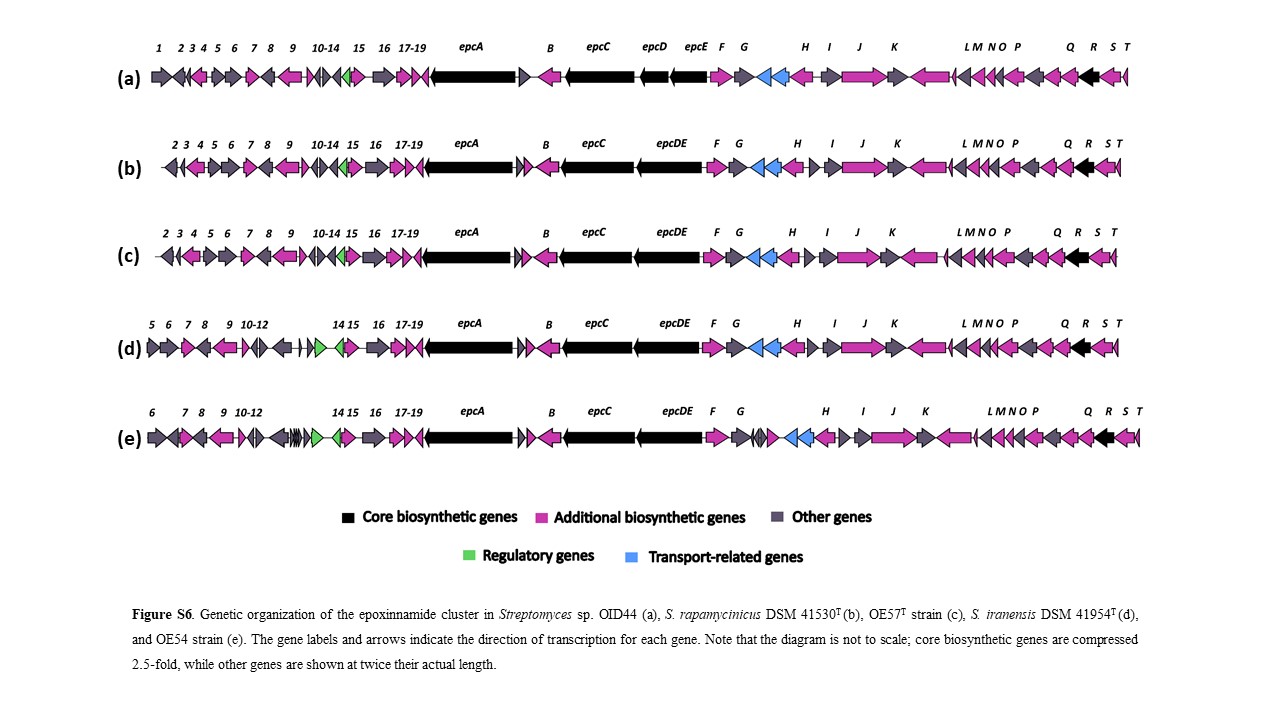

Supplement: Supplementary file 1 [file microorganisms-13-01622-s001.zip › Supplementary Figure S6.jpg]

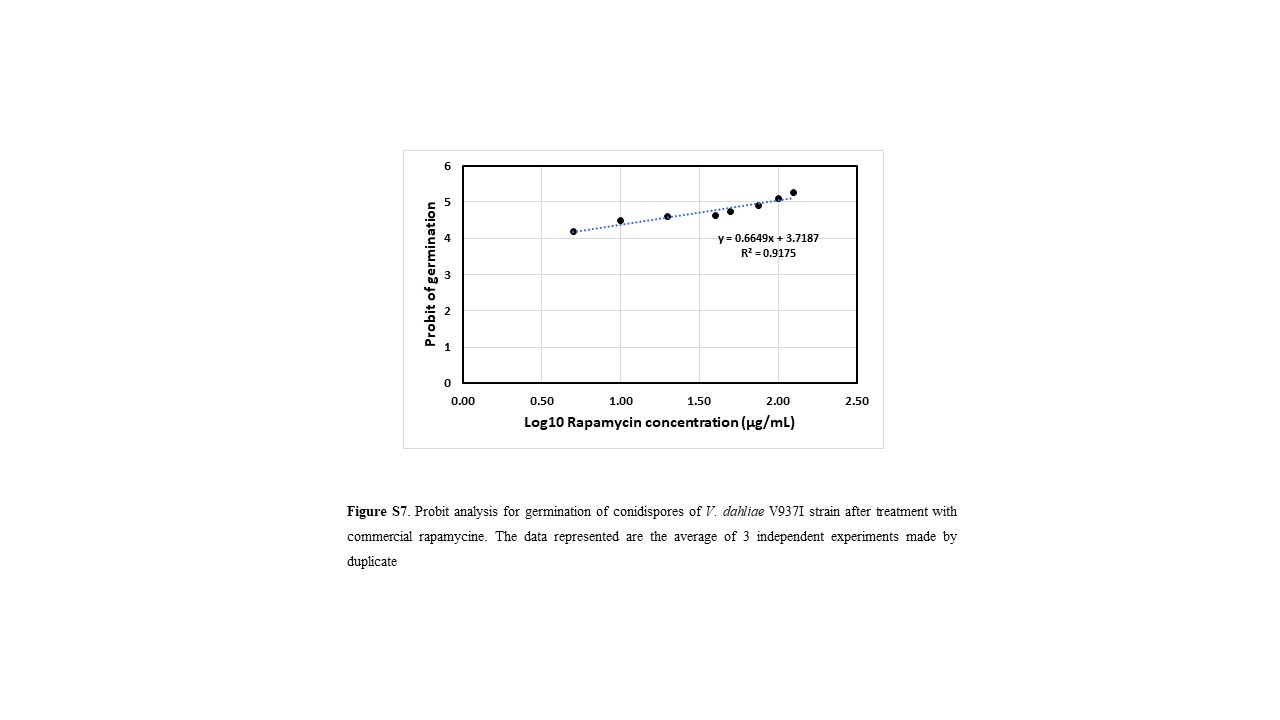

Supplement: Supplementary file 1 [file microorganisms-13-01622-s001.zip › Supplementary Figure S7.jpg]

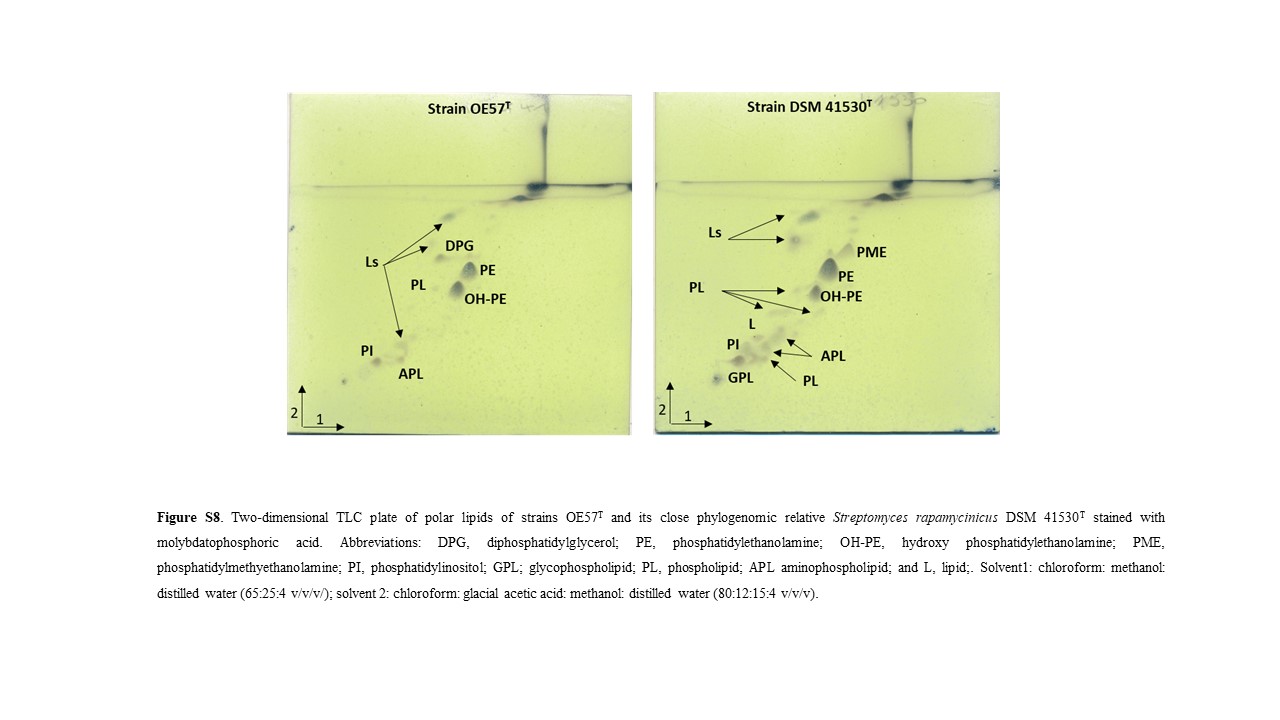

Supplement: Supplementary file 1 [file microorganisms-13-01622-s001.zip › Supplementary Figure S8.jpg]
